# Design, Synthesis, Antifungal Activity, and 3D-QSAR Study of Novel Quinoxaline-2-Oxyacetate Hydrazide

**DOI:** 10.3390/molecules29112501

**Published:** 2024-05-25

**Authors:** Peng Teng, Yufei Li, Ruoyu Fang, Yuchuan Zhu, Peng Dai, Weihua Zhang

**Affiliations:** Jiangsu Key Laboratory of Pesticide Science, College of Sciences, Nanjing Agricultural University, Nanjing 210095, China; tengpeng@njau.edu.cn (P.T.); 2019111022@njau.edu.cn (Y.L.); 2022811033@stu.njau.edu.cn (R.F.); 2021211010@stu.njau.edu.cn (Y.Z.); daipeng@njau.edu.cn (P.D.)

**Keywords:** quinoxaline, 2-oxyacetate hydrazide, antifungal activity, 3D-QSAR

## Abstract

Plant pathogenic fungi pose a major threat to global food security, ecosystem services, and human livelihoods. Effective and broad-spectrum fungicides are needed to combat these pathogens. In this study, a novel antifungal 2-oxyacetate hydrazide quinoxaline scaffold as a simple analogue was designed and synthesized. Their antifungal activities were evaluated against *Botrytis cinerea* (*B. cinerea*), *Altemaria solani* (*A. solani*), *Gibberella zeae* (*G. zeae*), *Rhizoctonia solani* (*R. solani*), *Colletotrichum orbiculare* (*C. orbiculare*), and *Alternaria alternata* (*A. alternata*). These results demonstrated that most compounds exhibited remarkable inhibitory activities and possessed better efficacy than ridylbacterin, such as compound **15** (EC_50_ = 0.87 μg/mL against *G. zeae*, EC_50_ = 1.01 μg/mL against *C. orbiculare*) and compound **1** (EC_50_ = 1.54 μg/mL against *A. alternata*, EC_50_ = 0.20 μg/mL against *R. solani*). The 3D-QSAR analysis of quinoxaline-2-oxyacetate hydrazide derivatives has provided new insights into the design and optimization of novel antifungal drug molecules based on quinoxaline.

## 1. Introduction

Crop pathogens pose a significant threat to food production, with fungicides serving as vital tools for disease control [1,2]. However, prolonged fungicide usage inevitably fosters increased pesticide resistance [3,4]. Therefore, the ongoing development of novel fungicides with distinctive pharmacodynamic frameworks and mechanisms of action is crucial to ensure sustainable agricultural development.

**Figure 1:**
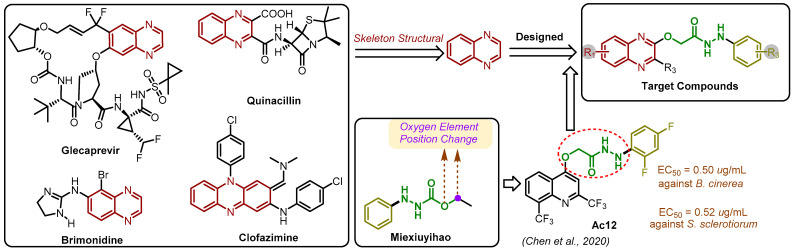
Design strategy of title compounds [31].

Natural products exhibit diverse structures, encompassing varied biological activities and unique mechanisms of action [5,6]. With excellent environmental compatibility, they serve as crucial sources of lead molecules for drug discovery [7,8]. Quinoxaline, a significant nitrogen-containing natural product, and its derivatives showcase a broad spectrum of biological activities [9,10,11], including antibacterial [12,13], antituberculosis [14], antimalarial [15], antiviral [16], anti-HIV [17], antifungal [18,19], herbicidal [20,21], and bacteriostatic [22] properties. Due to their extensive biological effects, quinoxaline derivatives hold pivotal positions in drug development research [23]. Notable examples include Glecaprevir, utilized in the treatment of chronic hepatitis C, and quinacillin, an antibiotic effective against severe bacterial infections (Figure 1).

In recent years, there has been a growing interest in the synthesis of hydrazides and their agricultural biological activities [24,25,26,27,28,29,30]. Compounds with a hydrazyl formate structure have demonstrated notable biological efficacy. For instance, the broad-spectrum fungicide Miexiuyihao (Figure 1) is a prominent example. Modifying the position of the oxygen element in the ester group has proven to be an effective strategy for enhancing biological activity. In 2021 [31], Chen et al. designed and synthesized quinoline 4-oxyacetate hydrazide derivatives, among which **Ac12** exhibited remarkable activity with EC_50_ values of 0.52 and 0.50 μg/mL against *S. sclerotiorum* and *B. cinerea*, surpassing the potency of both commercial fungicides azoxystrobin (both > 30.00 μg/mL) and 8-hydroxyquinoline (2.12 and 5.28 μg/mL). In this study, we focused on the design and synthesis of a novel series of quinoxolinylhydrazide derivatives. These compounds were systematically evaluated for their efficacy against six prominent crop pathogenic fungi through in vitro assays. Subsequently, the most promising compounds were chosen for a further investigation of their in vivo activity. Additionally, we sought to elucidate the primary mechanism of action using scanning electron microscopy (SEM). Furthermore, to gain deeper insights into the structure–activity relationship, a three-dimensional quantitative structure–activity relationship (3D-QSAR) model was established.

## 2. Results and Discussion

**Figure 2:**
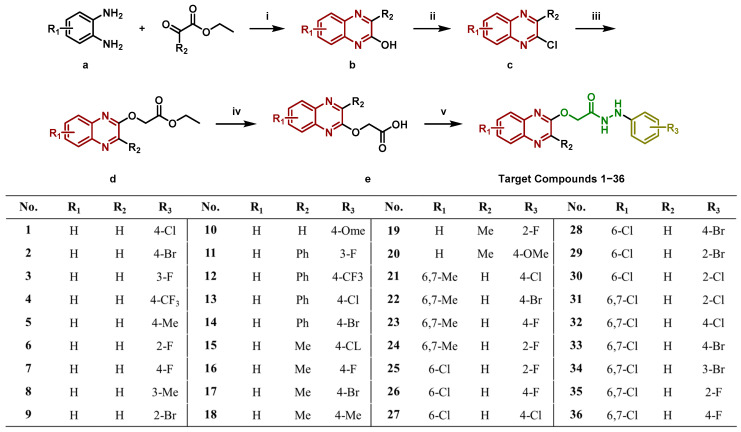
The synthetic route of the target compounds. Reagents and conditions. (i) H_2_O, SDS; (ii) POCl_3_, reflux; (iii) CsCO_3_, DMSO, 80 °C, 5 h; (iv) NaOH, H_2_O, rt; HCl, H_2_O, rt; and (v) TBTU, Et_3_N, CH_3_CN. (**a**–**e**) are reaction intermediate.

*Chemistry*. The synthetic route of the target compounds is shown in Figure 2. The structures of all compounds were identified by HRMS ^1^H NMR and ^13^C NMR spectroscopy (Supplementary Materials).

*In Vitro Antifungal Activity Screening.* The preliminary results, presented in Table 1, illustrated the significant inhibitory activity of the target compounds against six pathogenic fungi (*B. cinerea*, *A. solani*, *G. zeae*, *R. solani*, *C. orbiculare*, and *A. alternata*) at a concentration of 20.00 μg/mL. Notably, the inhibitory activity against *B. cinerea* stood out, with compounds **6**, **20**, and **24** achieving impressive inhibitory rates exceeding 90.0%, at 97.0%, 93.8%, and 93.7%, respectively, surpassing the control drug pyrimethanil at 75.1%. While the inhibitory effect against *A. solani* was slightly lower, compound **20** still exhibited notable inhibitory activity of 94.9%. Additionally, the inhibitory effect against *G. zeae* was remarkable, with compounds **1**, **2**, **7**, **15**, **16**, **18**, **26**, and **36** displaying inhibitory rates above 90.0%, at 95.8%, 93.1%, 99.7%, 92.2%, 93.6%, 91.4%, 98.9%, and 91.2%, respectively, surpassing the control drug pyrimethanil. The most effective inhibition was observed against *R. solani*, with 24 compounds exhibiting inhibitory rates above 90.0%, including 8 compounds that completely halted its growth. Furthermore, the inhibitory activity against *C. orbiculare* was noteworthy, with 6 compounds capable of completely suppressing mycelial growth. For *A. alternata*, the inhibitory activity was outstanding, with compounds **2** and **7** exhibiting perfect inhibitory rates of 100%, and compounds **1**, **3**, **6**, **16**, **26,** and **36** demonstrating inhibitory rates above 90.0%. These findings clearly surpass those of the control drug pyrimethanil. The preliminary analysis of structure–activity relationships indicated that compounds 11 through 14 exhibited a relatively low effectiveness, with notably weaker inhibitory effects against *R. solani* compared to other substituted compounds. While some compounds retained their inhibitory capacity against *C. orbiculare*, the overall effectiveness was reduced. The activity levels were generally sustained when the quinoxaline benzene ring featured 6,7-dimethyl substitutions. In contrast, chlorine substitution on the quinoxaline benzene ring led to a decrease in overall activity, yet these compounds were more effective against *R. solani*. Notably, the activity of hydrazide compounds was significantly enhanced with halogen substitutions, but not when substituted with trifluoromethyl. This variation in activity may be attributable not only to the electronic properties of the substituents but also to their size.

The EC_50_ values of the compounds with excellent antifungal activity were determined (Table 2). Compound **6** showed good activity against *B. cinerea*, with an EC_50_ value of 3.31 μg/mL, which was better than the control drug (3.39 μg/mL). Compound **20** exhibited better inhibitory activity against *A. solani* with an EC_50_ value of 4.42 μg/mL, superior to carbendazim (5.46 μg/mL). For *G. zeae*, 8 compounds (**1**, **2**, **7**, **15**, **16**, **18**, **26**, and **36**) demonstrated outstanding inhibitory activity; the EC_50_ values were all less than 2.00 μg/mL, significantly better than that of the control drug, ridylbacterin (2.20 μg/mL). Notably, compound **15** exhibited the highest activity, achieving an EC_50_ value of 0.87 μg/mL. For *C. orbiculare*, compounds **1**, **2**, **15**, **16**, **23**, **26**, **27**, and **36** showed the best inhibitory activity with EC_50_ values of 1.84, 1.32, 1.01, 1.35, 1.36, 1.61, 1.03, and 2.23 μg/mL, respectively, all better than carbendazim (2.32 μg/mL). Compounds **1** (EC_50_ = 1.54 μg/mL) and **7** (EC_50_ = 1.99 μg/mL) showed better inhibitory activity against *A. alternata*, better than the control drug ridylbacterin (EC_50_ = 2.07 μg/mL). For *R. solani*, 29 compounds showed excellent antifungal activity, with the EC_50_ value of target compounds being less than 1 μg/mL. Particularly, the EC_50_ value of compound **28** was 0.15 μg/mL, lower than the control drug pyrimethanil (EC_50_ = 0.21 μg/mL).

**Figure 3:**
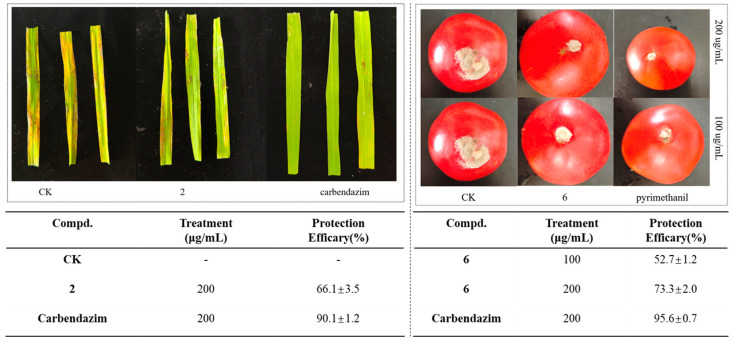
The in vivo preventive effects of compound **2** against *R. solani* and compound **6** against *B. cinerea*.

*In Vivo Antifungal Activity*. The inhibitory effect of compound **2** on *R. solani* in rice leaves was determined at a mass concentration of 200.00 μg/mL. As shown in Figure 3, the inhibition rate of compound **2** was 66.1% at this concentration. Compound **6**, which exhibited good activity, was selected for a tomato protection experiment, and its activity against *B. cinerea* was tested on tomato fruits at concentrations of 100.00 and 200.00 μg/mL. As depicted in Figure 3, compound **6** demonstrated significant inhibitory activity at both concentrations, with inhibition reaching 73.3% at 200.00 μg/mL. In vivo experiments demonstrated that compounds **2** and **6** retained some antifungal activity. They hold potential for further research and development.

**Figure 4:**
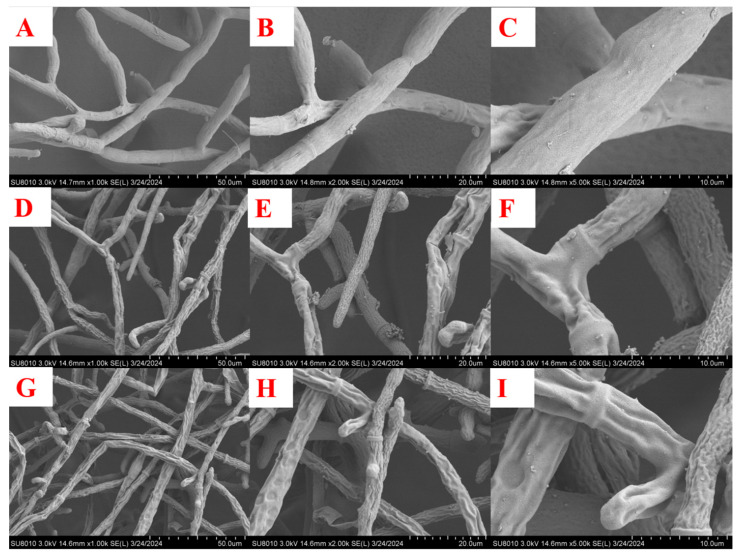
SEM images of *R. solani* hyphae treated by DMSO (**A**–**C**) and **2** (**D**–**I**).

*Scanning Electron Microscopy Observations*. Compound **2** was selected for scanning electron microscopy (SEM) to observe changes in the mycelium after treatment. DMSO served as the blank control. As depicted in Figure 4A–C, mycelia treated with DMSO exhibited a smooth, healthy state with a relatively uniform distribution. No folding, atrophy, or breakage of the mycelia were observed. In contrast, the mycelia of *R. solani* treated with compound **2** showed noticeable pathological changes. Figure 4D,G revealed that the mycelium of *R. solani* was disorderly distributed, intertwined, and overlapping. Some mycelia exhibited shrinkage, while Figure 4E,F,H,I showed evident pits and depressions, with some mycelia displaying distinctive protrusions that could be irregular stacks of metabolites. Overall, mycelia treated with compound **2** exhibited obvious lesions compared to those treated with DMSO, potentially affecting the direction and state of the mycelia growth by influencing the cell membrane structure of *R. solani*.

**Figure 5:**
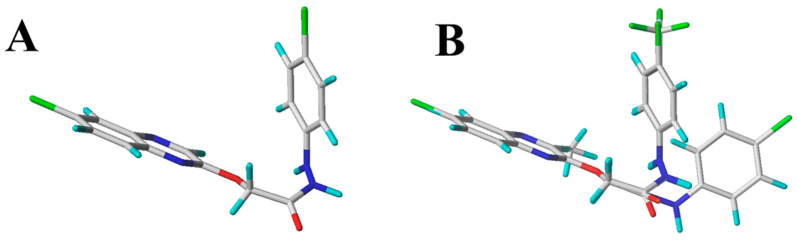
Template compound **28** (**A**) and composite renderings (**B**).

**Figure 6:**
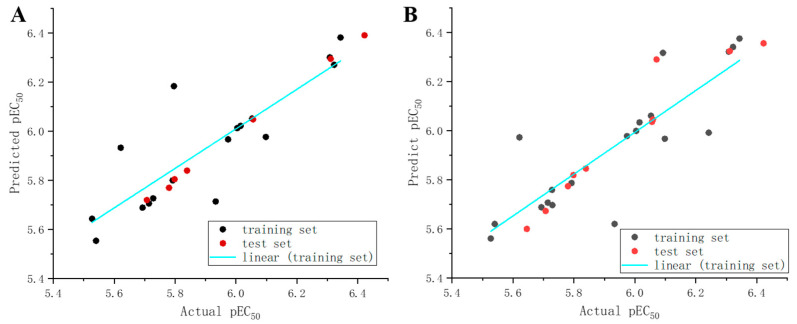
Experimental and predicted values of CoMFA (**A**) and CoMSIA (**B**) models pEC_50_.

*Study of 3D-QSAR Models Against R. solani*. To investigate the correlation between the molecular structure of quinoxolinylhydrazine derivatives and their inhibitory effectiveness against *R. solani*, effective 3D-QSAR models were developed. Then, 20 compounds (**2**–**4**, **6**, **12**, **13**, **15**–**19**, **22**, **24**, **26, 27**, **29**–**32**, and **34**) were randomly selected to be placed in the “training set” and 10 compounds (**1**, **5**, **9**, **14**, **20**, **23**, **25**, **28**, **33**, and **25**) were selected as the “test set”. These include both the comparative molecular field analysis (CoMFA) model and the comparative molecular similarity indices analysis (CoMSIA) model. Using compound **28** as the template, all compounds were superimposed. Figure 5A shows the energy-minimized structure of compound **28**, and Figure 5B displays the compound superposition diagram. Figure 6 illustrates the error and linear relationship between the pEC_50_ values predicted by the CoMFA and CoMSIA models and the experimental values.

A partial least squares (PLS) analysis was conducted to establish a correlation between the chemical structures and bioactivities of the target compounds. As presented in Table 3, both the CoMFA model (with q^2^ = 0.843 and r^2^ = 0.997) and the CoMSIA model (with q^2^ = 0.845 and r^2^ = 0.985) exhibited strong robustness and internal predictive power, fulfilling the criteria of q^2^ > 0.5 and r^2^ > 0.8 for a reliable forecasting ability. The pEC_50_ values (−log EC_50_) were chosen as the molecular activity data for each compound. Table 4 displays the activity prediction results of the CoMFA and CoMSIA models for both the “training set” and “test set” compounds, indicating a good alignment between the predicted and experimental values, with residuals falling within an acceptable error range.

**Figure 7:**
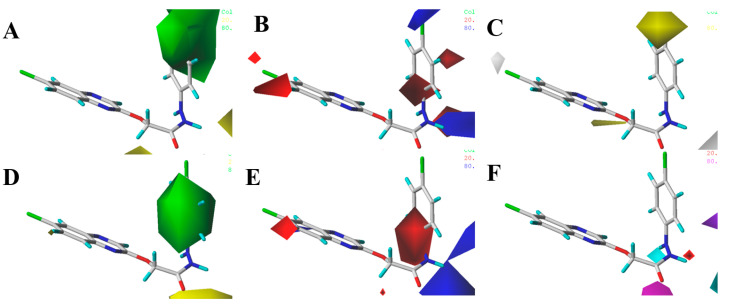
CoMFA (**A**,**B**) contour maps and CoMSIA (**C**–**F**) contour maps with compound **28** shown inside fields. Green and yellow modules indicate regions where steric bulk would increase and reduce anti-*R. solani* activity; blue and red modules indicate regions where electropositive and electronegative groups would increase anti-*R. solani* activity; yellow and grey modules indicate regions where hydrophobicity and hydrophilicity would increase anti-*R. solani* activity; magenta and red modules indicate regions where H-bond acceptor groups would increase and reduce the anti-*R. solani* activity, meanwhile, cyan and purple modules indicate regions where H-donor groups would increase and reduce anti-*R. solani* activity.

The following three conclusions can be drawn from the CoMFA and CoMSIA equipotential graphs shown in Figure 7. First, as depicted in Figure 7A,D, large green areas around compound **28** suggest that introducing large groups into phenylhy-drazine enhances anti-*R. solani* activity—for example, EC_50_ values **1**, **2**, **4** (R_1_ = H, R_2_ = H, R_3_ = 4-Cl, Br, CF_3_, EC_50_ = 0.20, 0.19, 0.17 μg/mL) > **7** (R_1_ = H, R_2_ = H, R_3_ = 4-F, EC_50_ = 0.65 μg/mL), **15** (R_1_ = H, R_2_ = Me, R_3_ = 4-Cl, EC_50_ = 0.16 μg/mL) > **16** (R_1_ = H, R_2_ = Me, R_3_ = 4-F, EC_50_ = 0.36 μg/mL), **27**, **28** (R_1_ = 6-Cl, R_2_ = H, R_3_ = 4-Cl, Br, EC_50_ = 0.18, 0.15 μg/mL) > **26** (R_1_ = 6-Cl, R_2_ = H, R_3_ = 4-F, EC_50_ = 0.55 μg/mL). Second, as illustrated in Figure 7B,E, a red region at the benzene ring site of compound **28** hydrazide indicates that electron-withdrawing groups at this site improve its antifungal efficacy—for example, EC_50_ values **1**, **2**, **7** (R_1_ = H, R_2_ = H, R_3_ = 4-Cl, Br, F, EC_50_ = 0.20, 0.19, 0.65 μg/mL) > **10** (R_1_ = H, R_2_ = H, R_3_ = 4-OMe, EC_50_ = 2.22 μg/mL), **15**, **16**, **17** (R_1_ = H, R_2_ = Me, R_3_ = 4-Cl, F, Br, EC_50_ = 0.16, 0.36, 0.32 μg/mL) >**18**, **20** (R_1_ = H, R_2_ = Me, R_3_ = 4-Me, OMe, EC_50_ = 0.52, 0.54 μg/mL); according to the in vitro activity data, the inhibition rate of **8** on *R. solani* was only 39.5%, which was also consistent with the rule. Third, also shown in Figure 7B,E, red color blocks at the quinoxaline benzene ring position suggest that electron-absorbing groups here increase the antifungal activity against *R. solani*—for example, EC_50_ values **26**, **27**, **28** (R_1_ = 6-Cl, R_2_ = H, R_3_ = 4-F, Cl, Br, EC_50_ = 0.55, 0.18, 0.15 μg/mL) > **1**, **2**, **7** (R_1_ = H, R_2_ = H, R_3_ = 4-Cl, Br, F, EC_50_ = 0.20, 0.19, 0.65 μg/mL), **28** (R_1_ = 6-Cl, R_2_ = H, R_3_ = 4-Br, EC_50_ = 0.15 μg/mL) > **22** (R_1_ = 6,7-Me, R_2_ = H, R_3_ = 4-Br, EC_50_ = 0.47 μg/mL). It can be seen from the preliminary screening data of in vitro activity that **25** and **35** could completely inhibit the growth of *R. solani*, and the inhibition rate of **24** was 92.7%. Figure 7 shows that yellow, green, red, and blue color blocks are concentrated on the benzene ring, indicating that various substituents on the benzene ring significantly influence anti-*R. solani* activity. This observation aligns with the results of activity tests. Generally, the addition of electron-withdrawing groups on the benzene ring enhances anti-*R. solani* activity, particularly when F, Cl, and Br are introduced at the fourth position, significantly boosting the activity. Chlorine substitution on the quinoxaline ring also enhanced the inhibitory activity against *R. solani*, although the improvement was modest. This corresponds with the small red color block on quinoxaline in Figure 7B,E, supporting the findings from the 3D-QSAR analysis.

## 3. Materials and Methods

*Instruments and Chemical Reagents.* The reagents and solvents utilized are commercially available and do not necessitate additional purification. ^1^H NMR and ^13^C NMR were measured by a BRUKER-400 NMR instrument (Bruker Corporation, Rheinstetten, Germany) with CDCl_3_, or DMSO-*d*_6_ as the solvent and tetramethylsilane (TMS) as the internal standard. High-resolution mass spectra (HRMS) data were collected with a Triple TOF 5600 plus LCMS spectrometer (AB Sciex, Framingham, MA, USA). The melting points (mp) were determined using the Buchi M-560 Melting Point Apparatus (BUCHI, Flawil, Switzerland).

*General Method of Synthesis.* The synthetic route of the compounds is shown in the Figure 2. The synthesis methods of intermediates *b* and *c* refer to the reported methods [18,32].

*General Synthesis method for the Intermediate d.* Intermediate *c* (5.00 mmol) and Caesium Carbonate (20.00 mmol) were added to 20.00 mL DMSO. We added ethyl glycolate (7.50 mmol) slowly. The reaction was stirred at 80 °C. The reaction was monitored using thin-layer chromatography (TLC) until the reaction was complete. We poured the mixture into cold water and extracted it by ethyl acetate. The organic phase was dried over Na_2_SO_4_. Subsequently, the solvent was evaporated to yield the crude product, which underwent purification via column chromatography.

*General Synthesis method for the Intermediate **e***. Intermediate *d* (5.00 mmol) was added to 20.00 mL 1.00 M NaOH. The reaction was stirred at room temperature and monitored using TLC until the reaction was complete. Then, we adjusted the mixture to acid with 10% HCl, and white solid precipitates formed. The intermediate *e* was obtained by filtration.

*General Synthesis method for Target Compounds* **1**–**36**. Intermediate *e* (1.00 mmol) and 2-(1*H*-Benzotriazole-1-yl)-1,1,3,3-tetramethyluronium tetrafluoroborate (TBTU, 1.20 mmol) were added to 4.00 mL CH_3_CN. Et_3_N (0.50 mL) was added. The reaction was stirred at room temperature and monitored using TLC until the reaction was complete. The solvent was evaporated to yield the crude product, which underwent purification via column chromatography.

*In Vitro Antifungal Activity Screening.* The antifungal activity of the target compounds against six pathogenic fungi was tested by mycelium growth method [33] (more details of the target compounds for the antifungal activities test procedure are shown in the Supplementary Materials), including *B. Cinerea*, *A. Solani*, *G. zeae*, *R. solani*, *C. Orbiculare*, and *A. Alternata*. The compounds dissolved in DMSO were subsequently introduced into a quantitative PDA (90.00 g glucose, 1000.00 g potato, 100.00 g agar, and 5.00 L water) medium to create a medicated plate, achieving a final concentration of 20.00 μg/mL. A plate was prepared with equal-volume DMSO as a blank control. All samples were incubated at 25 °C for 2–5 days, and each plate was assessed using the cross-streak method. Mean values and standard deviations (unit: cm) were computed, along with the inhibition rate following drug treatment. The calculation method is referred to as the reported method [34,35].

*In Vivo Antifungal Activity.* We prepared 500.00 mL of 1% Tsum 80 solution, weighed 10.00 mg of compound **2**, and dissolved it in 250.00 μL of DMSO. We used 1% Tsum 80 solution to bring the volume up to 50.00 mL, stirred well, and set it aside. In this case, the liquid concentration was 200.00 μg/mL. The blank control was prepared using 250.00 μL of DMSO and 1% Tsum 80 solution. We used the same method to prepare other required medications. In vivo antifungal activity was tested using reported methods [36,37,38].

Choose compounds exhibiting outstanding antifungal inhibition rates for in vitro EC_50_ determination. Five concentration gradients of 10.00, 5.00, 2.50, 1.25, and 0.625 μg/mL were established. We assess the inhibition rates of each concentration using the aforementioned method. We utilize the DPS data processing system to calculate the corresponding EC_50_ for each compound.

*Scanning Electron Microscopy Observations.* According to the above method, **2** plate containing drug concentration of 10.00 μg/mL was prepared, and blank control was set. *R. solani* was inoculated on the medicated plate and cultured for 36 h. The cake at the edge of the hypha was taken with a hole punch and fixed with phosphate buffer solution of glutaraldehyde. The mycelium was observed by scanning electron microscopy (Hitachi SU8010, Hitachi Co., Tokyo, Japan) with resolutions of 50.00 µm, 20.00 µm, and 10.00 µm.

*Research of 3D-QSAR.* Sybyl X-2.0 (Tripos, El Cerrito, CA, USA) was used to build 3D-QSAR models. The minimization of energy for the target molecular conformations was carried out utilizing the Tripos force field, along with the Gasteiger–Hückel method (with 10,000 iterations and 0.005 kcal mol-1 Å-1 convergence gradient), ensuring precision and thoroughness in the energy minimization. The characteristics of the CoMFA and CoMSIA models’ fields were determined through a thorough analysis employing partial least squares (PLS) regression.

## 4. Conclusions

In summary, a series of quinoxolinylhydrazide derivatives were designed and synthesized. Most of the compounds exhibited notable antifungal activity, with compound **1** showing superior inhibitory effects against six pathogenic fungi. The activity against *R. solani* was particularly impressive, with 29 compounds exhibiting EC_50_ values below 1.00 μg/mL, especially compound **28**, which had an EC_50_ of 0.15 μg/mL. In vivo antifungal activity experiments confirmed that compounds **2** and **6** maintained effectiveness within living organisms. Scanning electron microscopy results suggested that compound **2** was a potential inhibitor of *R. solani*. Reliable 3D-QSAR models targeting *R. solani* were successfully developed. The results of these experiments will help identify new potential fungicides.

## Figures and Tables

**Table 1:** Inhibitory activities of target compounds **1**–**36** against phytopathogenic fungi *^a^*.

Compd.	*B. cinrea*	*A. solani*	*G. zeae*	*R. solani*	*C. orbiculare*	*A. alternata*
**1**	86.1 ± 1.4	89.5 ± 1.2	95.8 ± 0.8	91.9 ± 0.9	100	96.4 ± 0.5
**2**	75.9 ± 0.5	48.1 ± 1.3	93.1 ± 0.6	94.4 ± 0.8	97.7 ± 0.5	100
**3**	88.8 ± 1.6	79.3 ± 0.7	59.7 ± 2.2	97.2 ± 0.7	100	91.2 ± 0.7
**4**	74.4 ± 1.6	66.4 ± 2.4	65.0 ± 1.2	77.4 ± 1.3	87.3 ± 1.1	85.0 ± 1.5
**5**	39.6 ± 3.0	40.7 ± 0.7	85.3 ± 0.6	96.4 ± 0.7	81.9 ± 0.6	60.9 ± 0.5
**6**	97.0 ± 0.6	80.6 ± 0	26.3 ± 0.7	70.2 ± 0.8	100	95.3 ± 2
**7**	80.8 ± 1.7	85.0 ± 0.7	99.7 ± 0.6	100	51.3 ± 1.0	100
**8**	10.2 ± 0.5	28.8 ± 1.0	<10	39.5 ± 0.8	<10	<10
**9**	85.6 ± 1.4	73.7 ± 1.2	29.2 ± 1.3	97.2 ± 0.7	56.2 ± 1.1	46.9 ± 0.5
**10**	21.9 ± 1.1	47.9 ± 0.7	31.9 ± 2.0	81.9 ± 2.1	50 ± 0.6	41.1 ± 0.7
**11**	14.7 ± 1.6	58.6 ± 1.0	12.8 ± 1.2	81.0 ± 0.7	83.5 ± 0.5	60.4 ± 0.7
**12**	52.2 ± 1.0	56.5 ± 1.9	25.6 ± 1.2	73.9 ± 2.2	50.1 ± 0.7	72.0 ± 0.6
**13**	56.7 ± 1.0	42.1 ± 0.7	25.8 ± 1.9	75.0 ± 0.8	35.6 ± 0.6	73.6 ± 0.8
**14**	28.0 ± 1.2	60.7 ± 0.7	23.9 ± 0.8	79.6 ± 1.2	34.4 ± 2.6	77.1 ± 0.7
**15**	80.6 ± 1.7	6.4 ± 0.7	92.2 ± 1.2	98.0 ± 0.7	91.7 ± 0.6	63.2 ± 0.7
**16**	70.6 ± 1.1	62.1 ± 0.7	93.6 ± 0.6	94.4 ± 0.8	95.6 ± 0.6	97.9 ± 0.7
**17**	74.6 ± 1.7	66.4 ± 0.7	84.7 ± 1.1	96.4 ± 0.7	60.1 ± 2.9	48.1 ± 0.7
**18**	81.6 ± 1.4	65.1 ± 0.8	91.4 ± 0.6	98.0 ± 1.3	94.8 ± 0.7	87.8 ± 2.4
**19**	20.4 ± 0.7	33.6 ± 0.7	42.2 ± 2.3	73.4 ± 0.8	18.8 ± 0.7	23.1 ± 0.7
**20**	93.8 ± 0.7	94.9 ± 1.4	48.1 ± 4.3	98.4 ± 0.7	100	63.8 ± 3.4
**21**	55.5 ± 2.0	35.0 ± 0.7	45.0 ± 1.0	73.4 ± 0.8	47.3 ± 0.5	33.1 ± 0.5
**22**	60.7 ± 2.4	76.4 ± 0.7	30.8 ± 0.8	97.2 ± 0.7	34.6 ± 0.6	39.6 ± 0.7
**23**	75.4 ± 0.7	69.1 ± 0.7	80.8 ± 1.6	97.6 ± 0.8	100	32.4 ± 0.7
**24**	93.7 ± 0.6	84.9 ± 1.2	25.8 ± 1.3	92.7 ± 0.7	67.2 ± 3.4	74.3 ± 0.6
**25**	41.5 ± 1.1	19.3 ± 0.7	15.8 ± 2.5	100	49.4 ± 0.6	50.7 ± 0.7
**26**	76.6 ± 1.1	62.1 ± 0.7	98.9 ± 0.8	100	100	91.9 ± 0.5
**27**	69.9 ± 2.6	46.4 ± 0.7	89.7 ± 2.2	95.2 ± 0.9	90.6 ± 0.6	56.3 ± 0.7
**28**	44.5 ± 2.0	39.3 ± 0.7	42.5 ± 1.6	78.0 ± 2.2	79.4 ± 0.6	45.8 ± 0.8
**29**	<10	20.0 ± 1.0	<10	100	14 ± 0.5	<10
**30**	61.7 ± 0.7	20.7 ± 0.7	33.1 ± 0.6	100	39.4 ± 0.6	54.9 ± 0.7
**31**	<10	23.7 ± 4.4	27.5 ± 4.4	96.8 ± 1.1	18.2 ± 1.1	22.2 ± 2.3
**32**	46.6 ± 1.1	54.6 ± 1.1	78.9 ± 2.6	83.9 ± 0.9	70.6 ± 1.4	60.2 ± 2.6
**33**	55.2 ± 3.2	53.3 ± 1.4	68.9 ± 1.9	92.5 ± 6.3	70.4 ± 1.3	48.4 ± 1.8
**34**	50.5 ± 1.5	70.7 ± 0.6	85.5 ± 1.1	100	65.9 ± 1.6	45.1 ± 1.4
**35**	14.1 ± 2.0	41.1 ± 0.8	24.8 ± 1.1	100	39.6 ± 1.7	29.3 ± 1.6
**36**	57.8 ± 3.3	86.1 ± 1.2	91.2 ± 0.5	100	100	92.0 ± 1.9
pyrimethanil	75.1 ± 1.0	43.1 ± 0.7	32.6 ± 1.8	89.4 ± 0.7	15.2 ± 1.1	27.2 ± 1.5

*^a^* Average of three replicates.

**Table 2:** Antifungal EC_50_ values of target compounds against phytopathogenic fungi *^a^*.

Pathogen	Compd.	EC_50_	Pathogen	Compd.	EC_50_
*B. Cinerea*	**6**	3.31 ± 0.18	*R. solani*	**1**	0.20 ± 0.07
	**20**	4.36 ± 0.10		**2**	0.19 ± 0.04
	**26**	4.90 ± 0.05		**3**	0.58 ± 0.03
	**pyrimethanil**	3.39 ± 0.22		**4**	0.17 ± 0.11
*A. solani*	**20**	4.42 ± 0.09		**5**	0.26 ± 0.07
	**carbendazim**	5.46 ± 0.14		**6**	0.58 ± 0.16
*G. zeae*	**1**	0.94 ± 0.03		**7**	0.65 ± 0.08
	**2**	1.22 ± 0.06		**9**	0.84 ± 0.20
	**7**	1.21 ± 0.02		**10**	2.22 ± 0.06
	**15**	0.87 ± 0.03		**11**	1.41 ± 0.11
	**16**	1.17 ± 0.08		**12**	1.05 ± 0.04
	**18**	1.77 ± 0.04		**13**	0.20 ± 0.01
	**28**	1.27 ± 0.06		**14**	0.39 ± 0.08
	**38**	1.54 ± 0.09		**15**	0.16 ± 0.04
	**pyrimethanil**	2.20 ± 0.11		**16**	0.36 ± 0.12
*C. orbiculare*	**1**	1.84 ± 0.07		**17**	0.32 ± 0.23
	**2**	1.32 ± 0.06		**18**	0.52 ± 0.03
	**3**	3.35 ± 0.22		**19**	0.33 ± 0.11
	**6**	8.39 ± 0.15		**20**	0.54 ± 0.14
	**15**	1.01 ± 0.11		**21**	0.15 ± 0.09
	**16**	1.35 ± 0.07		**22**	0.47 ± 0.04
	**18**	3.84 ± 0.10		**23**	0.54 ± 0.14
	**20**	3.86 ± 0.13		**24**	0.66 ± 0.11
	**23**	1.36 ± 0.14		**25**	0.68 ± 0.05
	**26**	1.61 ± 0.12		**26**	0.55 ± 0.05
	**27**	1.03 ± 0.11		**27**	0.18 ± 0.04
	**36**	2.23 ± 0.08		**28**	0.15 ± 0.08
	**carbendazim**	2.32 ± 0.11		**29**	1.21 ± 0.11
*A. alternata*	**1**	1.54 ± 0.12		**30**	1.05 ± 0.09
	**2**	10.75 ± 0.05		**31**	0.80 ± 0.11
	**3**	7.35 ± 0.21		**32**	0.23 ± 0.05
	**6**	12.09 ± 0.05		**33**	0.38 ± 0.07
	**7**	1.99 ± 0.08		**34**	0.36 ± 0.05
	**16**	4.82 ± 0.25		**35**	0.55 ± 0.23
	**28**	2.85 ± 0.06		**36**	0.50 ± 0.15
	**pyrimethanil**	2.07 ± 0.15		**pyrimethanil**	0.21 ± 0.10

*^a^* Average of three replicates.

**Table 3:** Statistical parameters of CoMFA and CoMSIA models.

Statistical Parameter	CoMFA	CoMSIA	Validation Criteria
*q* ^2 *a*^	0.843	0.845	>0.5
*r* ^2 *b*^	0.997	0.985	>0.8
s *^c^*	0.025	0.038	
F *^d^*	144.141	125.981	
ONC *^e^*	14	7	

*^a^* Cross-validated correlation. *^b^* Non-cross-validated correlation. *^c^* Standard error of estimate. *^d^* F-test value. *^e^* Optimum number of components.

**Table 4:** Actual and predicted pEC_50_ values of CoMFA and CoMSIA *^a^*.

Compd.	EC_50_	pEC_50_	CoMFA		CoMSIA	
			Pred pEC_50_ *^b^*	Residual	Pred pEC_50_ *^b^*	Residual
**1** *	0.20 ± 0.07	6.311	6.295	0.016	6.324	−0.013
**2**	0.19 ± 0.04	6.343	6.382	−0.039	6.375	−0.032
**3**	0.58 ± 0.03	5.728	5.727	0.001	5.759	−0.031
**4**	0.17 ± 0.11	6.322	6.271	0.051	6.341	−0.019
**5** *	0.26 ± 0.07	6.071	6.376	−0.305	6.29	−0.219
**6**	0.58 ± 0.16	5.729	6.193	−0.464	5.697	0.032
**9** *	2.22 ± 0.06	5.645	6.209	−0.564	5.6	0.045
**12**	1.05 ± 0.04	5.621	5.933	−0.312	5.973	−0.352
**13**	0.20 ± 0.01	6.015	6.022	−0.007	6.034	−0.019
**14** *	0.39 ± 0.08	6.056	6.049	0.007	6.036	0.02
**15**	0.12 ± 0.04	6.098	5.977	0.121	5.967	0.131
**16**	0.36 ± 0.12	5.974	5.967	0.007	5.978	−0.004
**17**	0.32 ± 0.23	6.004	6.013	−0.009	5.999	0.005
**18**	0.52 ± 0.03	5.792	5.800	−0.008	5.787	0.005
**19**	0.33 ± 0.11	6.053	6.052	0.001	6.061	−0.008
**20** *	0.54 ± 0.14	5.78	5.77	0.01	5.774	0.006
**22**	0.47 ± 0.04	5.933	5.714	0.219	5.62	0.313
**23** *	0.54 ± 0.14	5.798	5.805	−0.007	5.819	−0.021
**24**	0.66 ± 0.11	5.714	5.706	0.008	5.707	0.007
**25** *	0.68 ± 0.05	5.707	5.72	−0.013	5.673	0.034
**26**	0.55 ± 0.05	5.796	6.184	−0.388	6.253	−0.457
**27**	0.18 ± 0.04	6.308	6.301	0.007	6.322	−0.014
**28** *	0.15 ± 0.08	6.422	6.391	0.031	6.356	0.066
**29**	1.21 ± 0.11	5.527	5.644	−0.117	5.561	−0.034
**30**	1.05 ± 0.09	5.540	5.554	−0.014	5.62	−0.08
**31**	0.80 ± 0.11	5.693	5.689	0.004	5.688	0.005
**32**	0.23 ± 0.05	6.242	6.048	0.194	5.992	0.25
**33** *	0.38 ± 0.07	6.059	6.33	−0.271	6.045	0.014
**34**	0.36 ± 0.05	6.092	6.33	−0.238	6.317	−0.225
**35** *	0.55 ± 0.23	5.839	5.84	−0.001	5.846	−0.007

*^a^* Average of three replicates. *^b^* pEC_50_ = −log (EC_50_); the unit of EC_50_ value needs to be converted into mol/L. * Sample of the testing set.

## Data Availability

The data are contained within the article and Supplementary Materials.

## Supplementary Materials

The following supporting information can be downloaded at: https://www.mdpi.com/article/10.3390/molecules29112501/s1, Table S1. Antifungal EC_50_ values of target compounds against phytopathogenic fungia; Scheme S1. Keto-enol tautomerization of compound **3**.
