# Supplementary material for: Design, Synthesis, Antifungal Activity, and 3D-QSAR Study of Novel Quinoxaline-2-Oxyacetate Hydrazide
Source: Molecules. 2024 May 25;29(11):2501. doi: 10.3390/molecules29112501 (PMC11173898; doi:10.3390/molecules29112501)
Supplement: Supplementary file 1 [file molecules-29-02501-s001.zip › molecules-3010072-supplementary.pdf]

Design, Synthesis, Antifungal Activity and 3D-QSAR Study of Novel Quinoxaline-2-Oxyacetate Hydrazide

Peng Teng, YufeiLi, Ruoyu Fang, Yuchuan Zhu,Peng Dai and Weihua Zhang *

Jiangsu Key Laboratory of Pesticide Science, College of Sciences, Nanjing Agricultural University,

Nanjing 210095, China; tengpeng@njau.edu.cn (P.T.); 2019111022@njau.edu.cn (Y.L.); 2022811033@stu.njau.edu.cn

(R.F.); 2021211010@stu.njau.edu.cn (Y.Z.); daipeng@njau.edu.cn (P.D.)

*Correspondence: zhangweihua@njau.edu.cn

1. Fungicidal activity against six pathogenic fungi

The mycelial growth rate method was used to test the inhibitory effects of target molecules on various pathogens including *Rhizoctonia solani, Botrytis cinerea, Gibberella zeae, Alternaria solani, Colletotrichum orbiculare, and Alternaria alternata*.

Preparation of Potato Dextrose Agar (PDA) Medium:

Peel 200 g of potatoes and slice them. Boil the slices in approximately 1000 mL of water for about 30 minutes. When the potatoes are soft but not mushy, filter the mixture through two layers of cheesecloth into a graduated cylinder to remove residues and adjust the volume to 1000 mL with water. Add 18 g of agar to the filtrate and heat until dissolved. Then add 20 g of glucose, stir until evenly distributed, and dispense into Erlenmeyer flasks. Sterilize the medium at 121 °C and 1 Mpa for 20 minutes.

Preparation of Medication-Containing Medium:

Accurately weigh 10 mg of the compound to be tested, dissolve in 2 mL of DMSO to prepare a 5 mg/mL solution. Pipette 0.1 mL of this solution into 50 mL of sterilized PDA medium (resulting in a DMSO concentration of approximately 0.2%), and shake well to make a medium with a concentration of 10 μg/mL. Evenly pour this into three sterile 9 cm diameter Petri dishes. Use a medium prepared with an equal amount of DMSO (0.1 mL) as a solvent control. Other concentration stock solutions are diluted sequentially with DMSO.

Inoculation and Activity Measurement:

Remove the preserved fungal strains from the refrigerator at 4 °C, and activate them twice consecutively on fresh sterile PDA medium before use. Use a punch (internal diameter 0.5 cm) to take plugs from the edges of actively growing colonies. Inoculate the center of the medium plate (mycelium facing up) with these plugs using an inoculation needle. Incubate at 25 °C in an incubator. When the colony on the solvent control medium reaches 2/3 of the plate diameter (6.5 cm), measure its diameter. Measure the diameter of each colony twice using the cross method, and calculate the average (unit: cm). Set up three replicates for each concentration and the three controls. Calculate the growth inhibition rate after treatment with the following formula:

$$\text{Inhibition ratio (\%)} = \frac{\text{Solvent control colony diameter} - \text{Treat colony diameter}}{\text{Solvent control colony diameter} - 0.5} \times 100\%$$

2. Detailed EC_{50} data

Table S1. Antifungal EC_{50} values of target compounds against phytopathogenic fungi[a]

Pathogen	Compd.	EC_{50}	Regression equation	confidence	R^2

				**interval**	
B. cinerea	**6**	3.31±0.18	Y=1.4213X+4.2600	2.64-4.17	0.9864
	**20**	4.36±0.10	Y=2.1695X+3.6125	3.64-5.22	0.9901
	**24**	4.90±0.05	Y=1.6243X+3.8792	4.67-5.14	0.9991
	**pyrimethanil**	3.39±0.22	Y=1.4467X+4.2330	2.57-4.47	0.9759
A. solani	**20**	4.42±0.09	Y=1.6504X+3.9351	3.59-5.44	0.9871
	**carbendazim**	5.46±0.14	Y=1.5356X+3.8677	3.69-8.09	0.9638
G. zeae	**1**	0.94±0.03	Y=1.3200X+5.0329	0.84-1.06	0.9973
	**2**	1.22±0.06	Y=1.0556X+4.9096	0.96-1.54	0.9858
	**7**	1.21±0.02	Y=1.4149X+4.8820	1.14-1.29	0.9990
	**15**	0.87±0.03	Y=1.1326X+5.0682	0.75-1.00	0.9962
	**16**	1.17±0.08	Y=1.1706X+4.9220	0.88-1.55	0.9802
	**18**	1.77±0.04	Y=1.1055X+4.7271	1.55-2.01	0.9940
	**26**	1.27±0.06	Y=1.3608X+4.8597	1.06-1.52	0.9910
	**36**	1.54±0.09	Y=1.6035X+4.7012	1.24-1.90	0.9856
	**pyrimethanil**	2.20±0.11	Y=1.5239X+4.4782	1.81-2.67	0.9885
C. orbiculare	**1**	1.84±0.07	Y=1.7837X+4.5261	1.60-2.13	0.9923
	**2**	1.32±0.06	Y=1.7873X+4.7862	1.15-1.51	0.9947
	**3**	3.35±0.22	Y=4.3689X+2.7081	2.98-3.76	0.9941
	**6**	8.39±0.15	Y=2.4742X+2.7147	7.39-9.51	0.9965
	**15**	1.01±0.11	Y=1.6068X+4.9917	0.75-1.37	0.9805
	**16**	1.35±0.07	Y=1.9492X+4.7452	1.17-1.56	0.9942
	**18**	3.84±0.10	Y=1.8065X+3.9448	3.13-4.70	0.9862
	**20**	3.86±0.13	Y=1.6690X+4.0211	2.92-5.09	0.9784
	**23**	1.36±0.14	Y=2.2602X+4.6971	1.07-1.74	0.9831
	**26**	1.61±0.12	Y=1.8228X+4.6218	1.27-2.04	0.9817
	**27**	1.03±0.11	Y=2.0614X+4.9739	0.81-1.31	0.9873
	**36**	2.23±0.08	Y=1.7332X+4.3979	1.90-2.60	0.9901
	**carbendazim**	2.32±0.11	Y=2.3102X+4.1532	1.65-4.60	0.9815

A. alternata	**1**	1.54±0.12	Y=1.4515X+4.7261	1.14-2.10	0.9706
	**2**	10.75±0.05	Y=0.5297X+4.4537	8.49-13.61	0.9921
	**3**	7.35±0.21	Y=1.2065X+3.9548	5.31-10.17	0.9719
	**6**	12.09±0.05	Y=1.1770X+3.7257	9.38-15.60	0.9928
	**7**	1.99±0.08	Y=1.3167X+4.6078	1.75-2.25	0.994
	**16**	4.82±0.25	Y=1.7193X+3.8252	2.71-8.59	0.9179
	**26**	2.85±0.06	Y=1.5203X+4.3076	2.48-3.28	0.9923
	**pyrimethanil**	2.07±0.15	Y=0.6205X+4.7564	1.65-2.41	0.9732
R. solani	**1**	0.20±0.07	Y=0.6613X+5.6581	0.05-0.32	0.9877
	**2**	0.19±0.04	Y=0.5412X+5.5261	0.04-0.28	0.9683
	**3**	0.58±0.03	Y=1.5976X+5.3742	0.55-0.61	0.9996
	**4**	0.17±0.11	Y=0.5198X+5.3960	0.05-0.58	0.9361
	**5**	0.26±0.07	Y=0.5937X+5.3459	0.15-0.46	0.9801
	**6**	0.58±0.16	Y=1.9939X+5.4688	0.47-0.72	0.9934
	**7**	0.65±0.08	Y=1.6990X+5.3205	0.48-0.87	0.9864
	**9**	0.84±0.20	Y=2.1694X+5.1610	0.69-1.03	0.9909
	**10**	2.22±0.06	Y=1.0040X+4.6530	1.84-2.66	0.9950
	**11**	1.41±0.11	Y=1.5876X+4.7663	1.06-1.86	0.9970
	**12**	1.05±0.04	Y=0.8236X+4.9824	0.90-1.22	0.9948
	**13**	0.20±0.01	Y=0.5295X+5.5077	0.12-0.30	0.9971
	**14**	0.39±0.08	Y=1.0120X+5.4099	0.21-0.73	0.9681
	**15**	0.16±0.04	Y=0.6309X+5.5750	0.06-0.23	0.9845
	**16**	0.36±0.12	Y=0.7640X+5.3363	0.18-0.71	0.9640
	**17**	0.32±0.23	Y=1.0204X+5.5011	0.14-0.74	0.9524
	**18**	0.52±0.03	Y=0.8002X+5.2274	0.45-0.60	0.9989
	**19**	0.33±0.11	Y=0.6628X+5.3168	0.15-0.74	0.9739
	**20**	0.54±0.14	Y=2.0090X+5.5352	0.44-0.66	0.9948
	**21**	0.15±0.09	Y=0.7324X+5.5987	0.07-0.34	0.9846
	**22**	0.47±0.04	Y=1.5391X+5.5091	0.08-2.81	0.9910

**23**	0.54±0.14	Y=2.0090X+5.5352	0.15-0.74	0.9948
**24**	0.66±0.11	Y=1.7653X+5.3223	0.54-0.80	0.9942
**25**	0.68±0.05	Y=1.2728X+5.2139	0.55-0.84	0.9932
**26**	0.55±0.05	Y=1.1614X+5.2986	0.43-0.72	0.9918
**27**	0.18±0.04	Y=0.6490X+5.4865	0.12-0.26	0.9936
**28**	0.15±0.08	Y=0.5940X+5.4830	0.07-0.33	0.9746
**29**	1.21±0.11	Y=2.3761X+4.8060	0.99-1.47	0.9898
**33**	1.05±0.09	Y=1.5241X+4.9708	0.79-1.38	0.9773
**31**	0.80±0.11	Y=2.0342X+5.1939	0.71-0.91	0.9968
**32**	0.23±0.05	Y=0.8068X+5.5196	0.16-0.32	0.9932
**33**	0.38±0.07	Y=1.4781X+5.5311	0.28-2.81	0.9910
**34**	0.36±0.05	Y=0.7679X+5.3446	0.27-0.47	0.9935
**35**	0.55±0.23	Y=1.7925X+5.4638	0.38-0.79	0.9831
**36**	0.50±0.15	Y=1.2170X+5.3678	0.32-0.78	0.9784
**pyrimethanil**	0.21±0.10	Y=0.7552X+5.5126	0.10-0.43	0.9736

[a]Average of three replicates.

3. Characterization Data of Products 1-36

1 *N'*-(4-chlorophenyl)-2-(quinoxalin-2-yloxy)acetohydrazide

Yellow solid, yield: 73%, m.p. 197.4-198.8 °C. **^{1}H NMR** (400 MHz, DMSO-*d*$_6$) δ 10.15 (d, J = 2.5 Hz, 1H), 8.72 (s, 1H), 8.05 (d, J = 2.6 Hz, 2H), 7.97 – 7.76 (m, 2H), 7.75 – 7.61 (m, 1H), 7.11 (d, J = 8.5 Hz, 2H), 6.79 (d, J = 8.6 Hz, 2H), 5.10 (s, 2H). **^{13}C NMR** (100 MHz, DMSO-*d*$_6$) δ 167.82, 156.58, 148.59, 140.44, 139.64, 139.13, 131.06, 129.20, 128.85, 127.61, 127.25, 122.41, 114.24, 64.22. **HR-MS** (ESI): *m/z* calcd for $C_{16}H_{14}ClN_4O_2^+$ ([M+H]$^+$) 329.0800, found 329.0795.

2 *N'*-(4-bromophenyl)-2-(quinoxalin-2-yloxy)acetohydrazide

White solid, yield: 74%, m.p. 191.5-193.0 °C. **^{1}H NMR** (400 MHz, DMSO-*d*$_6$) δ 10.15 (s, 1H), 8.72 (s, 1H), 8.06 (dd, J = 10.3, 2.0 Hz, 2H), 7.92 – 7.79 (m, 2H), 7.68 (ddd, J = 8.3, 6.8, 1.6 Hz, 1H), 7.23 (d, J = 8.8 Hz, 2H), 6.76 (d, J = 8.8 Hz, 2H), 5.10 (s, 2H). **^{13}C NMR** (100 MHz, DMSO-*d*$_6$) δ 167.81, 156.58, 149.00, 140.44, 139.63, 139.13, 131.70, 131.07, 129.21, 127.62, 127.25, 114.75, 109.91, 64.21. **HR-MS** (ESI): *m/z* calcd for $C_{16}H_{14}BrN_4O_2^+$ ([M+H]$^+$) 373.0295, found 373.0296.

3 *N'*-(3-fluorophenyl)-2-(quinoxalin-2-yloxy)acetohydrazide

White solid, yield: 58%, m.p. 188.5-190.1 °C. **^{1}H NMR** (400 MHz, DMSO-*d*$_6$) δ 10.15 (s, 1H), 8.72 (s, 1H), 8.16 (d, J= 2.3 Hz, 1H), 8.05 (dd, J= 8.2, 1.5 Hz, 1H), 7.88 (dd, J= 8.4, 1.4 Hz, 1H), 7.81 (td, J= 6.9, 2.6 Hz, 1H), 7.68 (ddd, J= 8.4, 6.8, 1.5 Hz, 1H), 7.09 (td, J= 8.1, 6.7 Hz, 1H), 6.60 (dd, J= 8.2, 2.1 Hz, 1H), 6.52 (dt, J= 11.6, 2.3 Hz, 1H), 6.45 (td, J = 8.5, 2.5 Hz, 1H), 5.10 (s, 2H). **^{13}C NMR** (101 MHz, DMSO-*d*$_6$) δ 167.77, 162.45, 156.63, 151.84 (d, J = 10.3 Hz), 140.49, 139.61, 139.11, 130.99, 130.64 (d, J = 9.9 Hz), 129.17, 127.65, 127.30, 108.74, 105.12, 104.91, 99.26, 99.01, 64.16. **HR-MS** (ESI): *m/z* calcd for $C_{16}H_{14}FN_4O_2^+$ ([M+H]$^+$) 313.1095, found 313.1102.

4 2-(quinoxalin-2-yloxy)-*N'*-(4-(trifluoromethyl)phenyl)acetohydrazide

White solid, yield: 36%, m.p. 201.4-203.0 °C. **^{1}H NMR** (400 MHz, DMSO-*d*$_6$) δ 10.33 (s, 1H), 8.73 (s, 1H), 8.52 (s, 1H), 8.06 (d, J = 8.4 Hz, 1H), 7.89 (dd, J = 23.6, 7.8 Hz, 2H), 7.70 (t, J = 7.7 Hz, 1H), 7.39 (d, J = 8.4 Hz, 2H), 6.88 (d, J = 8.3 Hz, 2H), 5.12 (s, 2H). **^{13}C NMR** (100 MHz, DMSO-*d*$_6$) δ 167.77, 156.62, 150.69, 140.48, 139.62, 139.12, 138.39, 131.13, 129.22, 127.70, 127.29, 126.47, 122.73, 111.99, 64.11. **HR-MS** (ESI): *m/z* calcd for $C_{17}H_{14}F_3N_4O_2^+$ ([M+H]$^+$) 363.1063, found 363.1070.

5 2-(quinoxalin-2-yloxy)-*N'*-(p-tolyl)acetohydrazide

White solid, yield: 52%, m.p. 187.6-188.8 °C. **^{1}H NMR** (400 MHz, DMSO-d_6) δ 10.03 (s, J = 2.9 Hz, 1H), 8.70 (s, 1H), 8.06 (dd, J = 8.2, 1.4 Hz, 1H), 7.98 – 7.79 (m, 2H), 7.73 – 7.54 (m, 2H), 6.89 (d, J = 8.0 Hz, 2H), 6.68 (d, J = 8.4 Hz, 2H), 5.07 (s, 2H), 2.15 (s, 3H). **^{13}C NMR** (100 MHz, DMSO-d_6) δ 167.63, 156.62, 147.31, 140.50, 139.67, 139.11, 131.04, 129.48, 129.19, 127.62, 127.58, 127.28, 112.95, 64.19, 20.61. **HR-MS** (ESI): *m/z* calcd for $C_{17}H_{17}N_4O_2^+$ ([M+H]$^+$) 309.1346, found 309.1344.

6 *N'*-(2-fluorophenyl)-2-(quinoxalin-2-yloxy)acetohydrazide

Yellow solid, yield: 76%, m.p. 197.4-198.8 °C. **^{1}H NMR** (400 MHz, DMSO-d_6) δ 10.17 (s, 1H), 8.72 (s, 1H), 8.05 (dd, J = 8.2, 1.4 Hz, 1H), 8.02 – 7.73 (m, 3H), 7.69 (ddd, J = 8.4, 6.9, 1.6 Hz, 1H), 7.07 (d, J = 1.3 Hz, 1H), 6.99 – 6.80 (m, 2H), 6.69 (ddd, J = 5.8, 4.3, 2.2 Hz, 1H), 5.10 (s, 2H). **^{13}C NMR** (100 MHz, DMSO-d_6) δ 167.74, 156.61, 151.74, 149.36, 140.49, 139.63, 139.12, 137.10 (d, J = 10.4 Hz), 131.10, 129.21, 127.66, 127.24, 124.79, 119.10, 115.25 (d, J = 17.6 Hz), 114.19, 64.16. **HR-MS** (ESI): *m/z* calcd for $C_{16}H_{14}FN_4O_2^+$ ([M+H]$^+$) 313.1095, found 313.1102.

7 *N'*-(4-fluorophenyl)-2-(quinoxalin-2-yloxy)acetohydrazide

Yellow solid, yield: 77%, m.p. 191.2-192.0 °C. **^{1}H NMR** (400 MHz, DMSO-d_6) δ 10.12 (d, J = 2.8 Hz, 1H), 8.71 (s, 1H), 8.05 (dd, J = 8.2, 1.4 Hz, 1H), 8.02 – 7.76 (m, 3H), 7.68 (ddd, J = 8.3, 6.7, 1.7 Hz, 1H), 6.92 (t, J = 8.9 Hz, 2H), 6.88 – 6.51 (m, 2H), 5.08 (s, 2H). **^{13}C NMR** (100 MHz, DMSO-d_6) δ 167.71, 156.62, 146.12, 140.50, 139.63, 139.10, 131.13, 129.20, 127.65, 127.27, 115.59, 115.37, 113.88 (d, J = 7.5 Hz), 64.18. **HR-MS** (ESI): *m/z* calcd for $C_{16}H_{14}FN_4O_2^+$ ([M+H]$^+$) 313.1095, found 313.1103.

8 2-(quinoxalin-2-yloxy)-*N'*-(m-tolyl)acetohydrazide

Yellow solid, yield: 73%, m.p. 183.9-185.3 °C. **^{1}H NMR** (400 MHz, DMSO-d_6) δ 8.70 (s, 1H), 8.02 (d, J = 8.1 Hz, 1H), 7.91 (d, J = 8.4 Hz, 1H), 7.85 – 7.72 (m, 2H), 7.72 – 7.59 (m, 2H), 7.45 – 6.74 (m, 2H), 5.01 (s, 2H), 3.04 (s, 3H). **^{13}C NMR** (100 MHz, DMSO-d_6) δ 170.00, 156.59, 140.03, 139.66, 139.05, 131.08, 129.17, 127.58, 127.36, 126.83, 124.49, 119.32, 110.48, 63.06, 8.88. **HR-MS** (ESI): *m/z* calcd for $C_{17}H_{17}N_4O_2^+$ ([M+H]$^+$) 309.1346, found 309.1340.

9 *N'*-(2-bromophenyl)-2-(quinoxalin-2-yloxy)acetohydrazide

Yellow solid, yield: 85%, m.p. 195.3-196.9 °C. **^{1}H NMR** (400 MHz, DMSO-d_6) δ 10.30

(s, 1H), 8.73 (s, 1H), 8.06 (dd, *J* = 8.3, 1.4 Hz, 1H), 7.90 (dd, *J* = 8.3, 1.5 Hz, 1H), 7.84 (ddd, *J* = 8.2, 6.8, 1.5 Hz, 1H), 7.69 (ddd, *J* = 8.2, 6.8, 1.5 Hz, 1H), 7.41 (dd, *J* = 7.8, 1.4 Hz, 1H), 7.36 (s, 1H), 7.08 (td, *J* = 7.8, 7.3, 1.4 Hz, 1H), 6.91 (dd, *J* = 8.2, 1.5 Hz, 1H), 6.66 (td, *J* = 7.6, 1.6 Hz, 1H), 5.11 (s, 2H). **^{13}C NMR** (100 MHz, DMSO-*d*$_6$) *δ* 167.64, 156.59, 145.91, 140.48, 139.61, 139.13, 132.78, 131.13, 129.23, 128.59, 127.69, 127.25, 120.64, 113.83, 107.30, 64.19. **HR-MS** (ESI): *m/z* calcd for $C_{16}H_{14}BrN_4O_2^+$ ([M+H]$^+$) 373.0295, found 373.0301.

10 *N'*-(4-methoxyphenyl)-2-(quinoxalin-2-yloxy)acetohydrazide

White solid, yield: 46%, m.p. 180.1-182.0 °C. **^{1}H NMR** (400 MHz, DMSO-*d*$_6$) *δ* 10.11 (d, *J* = 3.1 Hz, 1H), 8.71 (d, *J* = 4.7 Hz, 1H), 8.18 – 7.98 (m, 1H), 7.97 – 7.76 (m, 3H), 7.69 (ddd, *J* = 8.3, 6.7, 1.7 Hz, 1H), 7.54 (d, *J* = 3.1 Hz, 1H), 6.95 (d, *J* = 1.9 Hz, 1H), 6.86 – 6.33 (m, 4H), 5.07 (s, 2H), 3.63 (s, 3H). **^{13}C NMR** (100 MHz, DMSO-*d*$_6$) *δ* 167.57, 156.64, 153.03, 143.44, 140.51, 139.66, 139.09, 131.05, 129.19, 127.63, 127.32, 114.52, 114.14, 64.17, 55.67. **HR-MS** (ESI): *m/z* calcd for $C_{17}H_{17}N_4O_3^+$ ([M+H]$^+$) 325.1295, found 325.1301.

11 *N'*-(3-fluorophenyl)-2-((3-phenylquinoxalin-2-yl)oxy)acetohydrazide

White solid, yield: 79%, m.p. 186.1-187.6 °C. **^{1}H NMR** (400 MHz, DMSO-*d*$_6$) *δ* 10.28 (s, 1H), 8.28 – 8.06 (m, 4H), 8.00 – 7.65 (m, 3H), 7.55 (d, *J* = 5.3 Hz, 3H), 7.10 (q, *J* = 7.7 Hz, 1H), 6.75 – 6.32 (m, 3H), 5.16 (s, 2H). **^{13}C NMR** (100 MHz, DMSO-*d*$_6$) *δ* 167.47, 164.10, 162.08, 155.43, 148.18 (d, *J* = 7.9 Hz), 142.60, 141.29, 140.21, 134.94, 129.95, 129.86, 129.71, 129.62, 129.13, 129.03, 127.95, 126.73, 101.42 (d, *J* = 19.8 Hz), 64.50. **HR-MS** (ESI): *m/z* calcd for $C_{22}H_{18}FN_4O_2^+$ ([M+H]$^+$) 388.1336, found 388.1331.

12 *N'*-(4-chlorophenyl)-2-((3-phenylquinoxalin-2-yl)oxy)acetohydrazide

Yellow solid, yield: 74%, m.p. 203.1-204.5 °C. **^{1}H NMR** (400 MHz, DMSO-*d*$_6$) *δ* 10.22 (s, 1H), 8.30 (d, *J* = 6.7 Hz, 2H), 8.11 (s, 1H), 7.93 (d, *J* = 7.9 Hz, 1H), 7.66 (d, *J* = 8.0 Hz, 1H), 7.53 (d, *J* = 6.5 Hz, 4H), 7.43 (t, *J* = 7.6 Hz, 1H), 7.19 (d, *J* = 8.3 Hz, 2H), 6.79 (d, *J* = 8.4 Hz, 2H), 5.14 (s, 2H)..**^{13}C NMR** (100 MHz, DMSO-*d*$_6$) *δ* 168.03, 154.94, 148.94, 146.67, 140.86, 139.20, 135.99, 130.78, 130.28, 130.17, 130.07, 129.19, 128.58, 127.94, 126.79, 122.27, 113.37, 64.50. **HR-MS** (ESI): *m/z* calcd for $C_{23}H_{17}F_3N_4O_2^+$ ([M+H]$^+$) 439.1376, found 439.1384.

13 *N'*-(4-chlorophenyl)-2-((3-phenylquinoxalin-2-yl)oxy)acetohydrazide

Yellow solid, yield: 83%, m.p. 195.9-197.1 °C. **^{1}H NMR** (400 MHz, DMSO-d_6) δ 10.17 (d, J = 2.4 Hz, 1H), 8.42 – 8.07 (m, 4H), 8.07 – 7.80 (m, 1H), 7.71 (s, 1H), 7.56 (dd, J = 5.1, 2.0 Hz, 3H), 7.17 – 7.04 (m, 2H), 6.94 – 6.41 (m, 2H), 5.16 (s, 2H). **^{13}C NMR** (100 MHz, DMSO-d_6) δ 167.95, 154.95, 148.65, 146.69, 139.21, 135.99, 130.87, 130.30, 130.17, 129.20, 128.84, 128.59, 127.97, 126.79, 122.25, 114.18, 64.52. **HR-MS** (ESI): *m/z* calcd for $C_{22}H_{18}ClN_4O_2^+$ ([M+H]$^+$) 405.1113, found 405.1105.

14 *N'*-(4-bromophenyl)-2-((3-phenylquinoxalin-2-yl)oxy)acetohydrazide

Yellow solid, yield: 87%, m.p. 184.5-185.7 °C. **^{1}H NMR** (400 MHz, DMSO-d_6) δ 10.16 (s, 1H), 8.73 – 8.09 (m, 3H), 8.07 – 7.69 (m, 3H), 7.70 – 7.43 (m, 4H), 7.23 (d, J = 8.3 Hz, 2H), 6.77 (d, J = 8.4 Hz, 2H), 5.15 (s, 2H). **^{13}C NMR** (100 MHz, DMSO-d_6) δ 167.93, 154.94, 149.04, 139.20, 135.97, 131.67, 130.87, 130.30, 130.16, 129.20, 128.59, 127.98, 126.79, 114.69, 64.50. **HR-MS** (ESI): *m/z* calcd for $C_{22}H_{18}BrN_4O_2^+$ ([M+H]$^+$) 449.0602, found 449.0606.

15 *N'*-(4-chlorophenyl)-2-((3-methylquinoxalin-2-yl)oxy)acetohydrazide

Yellow solid, yield: 65%, m.p. 207.6-208.9 °C. **^{1}H NMR** (400 MHz, DMSO-d_6) δ 10.11 (d, J = 2.3 Hz, 1H), 8.05 (d, J = 2.4 Hz, 1H), 7.95 (d, J = 8.3 Hz, 1H), 7.84 (d, J = 8.2 Hz, 1H), 7.76 (t, J = 7.6 Hz, 1H), 7.63 (t, J = 7.5 Hz, 1H), 7.08 (d, J = 8.4 Hz, 2H), 6.77 (d, J = 8.4 Hz, 2H), 5.08 (s, 2H), 2.64 (s, 3H). **^{13}C NMR** (100 MHz, DMSO-d_6) δ 167.83, 155.63, 148.66 (d, J = 11.8 Hz), 139.15, 138.86, 129.80, 128.77, 128.31, 127.42, 126.85, 122.14, 114.16, 64.50, 20.74. **HR-MS** (ESI): *m/z* calcd for $C_{17}H_{16}ClN_4O_2^+$ ([M+H]$^+$) 343.0956, found 343.0962.

16 *N'*-(4-fluorophenyl)-2-((3-methylquinoxalin-2-yl)oxy)acetohydrazide

Yellow solid, yield: 77%, m.p. 200.1-201.3 °C. **^{1}H NMR** (400 MHz, DMSO-d_6) δ 10.15 (d, J = 2.5 Hz, 1H), 8.72 (s, 1H), 8.05 (d, J = 2.6 Hz, 2H), 7.97 – 7.76 (m, 2H), 7.75 – 7.61 (m, 1H), 7.11 (d, J = 8.5 Hz, 2H), 6.79 (d, J = 8.6 Hz, 2H), 5.10 (s, 2H). **^{13}C NMR** (100 MHz, DMSO-d_6) δ 167.82, 157.38, 155.65, 148.75, 146.14, 139.01 (d, J = 30.8 Hz), 129.81, 128.31, 127.41, 126.87, 115.44 (d, J = 22.6 Hz), 113.87 (d, J = 7.7 Hz), 64.52, 20.74. **HR-MS** (ESI): *m/z* calcd for $C_{17}H_{16}FN_4O_2^+$ ([M+H]$^+$) 327.1252, found 327.1257.

17 *N'*-(4-bromophenyl)-2-((3-methylquinoxalin-2-yl)oxy)acetohydrazide

White solid, yield: 71%, m.p. 219.2-220.2 °C. **^{1}H NMR** (400 MHz, DMSO-d_6) δ 10.04

(d, J = 2.4 Hz, 1H), 8.03 (d, J = 2.4 Hz, 1H), 8.00 – 7.95 (m, 1H), 7.85 (dd, J = 8.2, 1.4 Hz, 1H), 7.80 – 7.71 (m, 1H), 7.64 (td, J = 7.6, 6.9, 1.5 Hz, 1H), 7.20 (d, J = 8.7 Hz, 2H), 6.91 – 5.99 (m, 2H), 5.08 (s, 2H), 2.65 (s, 3H). **^{13}C NMR** (100 MHz, DMSO-d_6) δ 167.84, 155.62, 149.00, 148.75, 139.15, 138.88, 131.64, 129.80, 128.33, 127.43, 126.86, 114.69, 109.73, 64.52, 20.75. **HR-MS** (ESI): *m/z* calcd for $C_{17}H_{16}BrN_4O_2^+$ ([M+H]$^+$) 387.0451, found 387.0455.

18 2-((3-methylquinoxalin-2-yl)oxy)-*N'*-(p-tolyl)acetohydrazide

Yellow solid, yield: 74%, m.p. 215.3-216.9 °C. **^{1}H NMR** (400 MHz, DMSO-d_6) δ *10.28* (d, J = 2.0 Hz, 1H), 8.59 (s, 1H), 8.50 (d, J = 2.0 Hz, 1H), 7.79 (s, 1H), 7.65 (s, 1H), 7.39 (d, J = 8.5 Hz, 2H), 6.90 (d, J = 8.5 Hz, 2H), 5.07 (s, 2H), 2.45 (s, 3H), 2.40 (s, 3H). **^{13}C NMR** (100 MHz, DMSO-d_6) δ 167.92, 156.30, 152.83, 141.02, 138.96, 138.14, 137.93, 137.22, 128.37, 126.77, 126.58, 126.41, 118.52, 112.09, 64.00, 20.12, 19.85. **HR-MS** (ESI): *m/z* calcd for $C_{18}H_{19}N_4O_2^+$ ([M+H]$^+$) 323.1503, found 323.1508.

19 *N'*-(2-fluorophenyl)-2-((3-methylquinoxalin-2-yl)oxy)acetohydrazide

Yellow solid, yield: 67%, m.p. 204.5-205.9 °C. **^{1}H NMR** (400 MHz, DMSO-d_6) δ 10.08 (d, J = 2.1 Hz, 1H), 7.97 (dd, J = 8.2, 1.5 Hz, 1H), 7.88 – 7.80 (m, 2H), 7.76 (ddd, J = 8.4, 7.0, 1.5 Hz, 1H), 7.64 (ddd, J = 8.4, 7.0, 1.5 Hz, 1H), 7.05 (ddd, J = 12.1, 8.0, 1.3 Hz, 1H), 6.92 (td, J = 8.3, 1.8 Hz, 1H), 6.88 – 6.82 (m, 1H), 6.68 (tdd, J = 7.4, 4.7, 1.7 Hz, 1H), 5.10 (s, 2H), 2.66 (s, 3H). **^{13}C NMR** (100 MHz, DMSO-d_6) δ 167.85, 155.62, 148.74, 139.02 (d, J = 27.2 Hz), 137.10 (d, J = 10.8 Hz), 129.76, 128.32, 127.41, 126.84, 124.73, 119.08 (d, J = 6.7 Hz), 115.23 (d, J = 17.3 Hz), 114.21, 64.50, 20.73. **HR-MS** (ESI): *m/z* calcd for $C_{17}H_{16}FN_4O_2^+$ ([M+H]$^+$) 327.1252, found 327.1258.

20 *N'*-(4-methoxyphenyl)-2-((3-methylquinoxalin-2-yl)oxy)acetohydrazide

Yellow solid, yield: 56%, m.p. 189.2-190.3 °C. **^{1}H NMR** (400 MHz, DMSO-d_6) δ 9.98 (d, J = 3.1 Hz, 1H), 8.18 – 7.33 (m, 6H), 6.84 – 6.12 (m, 7H), 5.06 (s, 2H), 3.62 (s, 3H), 2.65 (s, 3H). **^{13}C NMR** (100 MHz, DMSO-d_6) δ 167.69, 153.00, 129.70, 128.29, 127.38, 126.92, 114.50, 114.13, 64.52, 55.66, 20.73. **HR-MS** (ESI): *m/z* calcd for $C_{18}H_{19}N_4O_2^+$ ([M+H]$^+$) 339.1452, found 339.1455.

21 *N'*-(4-chlorophenyl)-2-((6,7-*d*imethylquinoxalin-2-yl)oxy)acetohydrazide

Yellow solid, yield: 76%, m.p. 198.0-199.0 °C. **^{1}H NMR** (400 MHz, DMSO-d_6) δ 10.17 (d, J = 2.4 Hz, 1H), 8.57 (s, 1H), 8.04 (d, J = 2.4 Hz, 1H), 7.78 (s, 1H), 7.62 (s, 1H),

7.09 (d, *J* = 8.4 Hz, 2H), 6.78 (d, *J* = 8.4 Hz, 2H), 5.03 (s, 2H), 2.45 (s, 3H), 2.40 (s, 3H). **^{13}C NMR** (100 MHz, DMSO-*d*$_6$) *δ* 167.82, 156.31, 148.62, 141.04, 138.98, 138.03 (d, *J* = 25.1 Hz), 137.21, 128.78, 128.35, 126.56, 122.19, 114.23, 63.99, 20.25, 19.90. **HR-MS** (ESI): *m/z* calcd for $C_{18}H_{18}ClN_4O_2^+$ ([M+H]$^+$) 357.1113, found 357.1110.

22 *N'*-(4-bromophenyl)-2-((6,7-*d*imethylquinoxalin-2-yl)oxy)acetohydrazide

Yellow solid, yield: 79%, m.p. 208.1-209.4 °C. **^{1}H NMR** (400 MHz, DMSO-*d*$_6$) *δ* 10.19 (d, *J* = 2.4 Hz, 1H), 8.58 (s, 1H), 8.07 (d, *J* = 2.4 Hz, 1H), 7.78 (s, 1H), 7.62 (s, 1H), 7.21 (d, *J* = 8.8 Hz, 2H), 6.75 (d, *J* = 8.8 Hz, 2H), 5.04 (s, 2H), 2.45 (s, 3H), 2.40 (s, 3H). **^{13}C NMR** (100 MHz, DMSO-*d*$_6$) *δ* 167.82, 156.30, 149.03, 141.02, 138.97, 138.15, 137.90, 137.20, 131.63, 128.36, 126.57, 114.75, 109.70, 63.99, 20.25, 19.91. **HR-MS** (ESI): *m/z* calcd for $C_{18}H_{18}BrN_4O_2^+$ ([M+H]$^+$) 401.0608, found 401.0610.

23 2-((6,7-*d*imethylquinoxalin-2-yl)oxy)-*N'*-(4-fluorophenyl)acetohydrazide

Yellow solid, yield: 87%, m.p. 194.8-196.5 °C. **^{1}H NMR** (400 MHz, DMSO- *d*6) *δ* 10.18 (d, *J* = 2.8 Hz, 1H), 8.57 (s, 1H), 7.97 – 7.76 (m, 2H), 7.62 (s, 1H), 7.08 – 6.35 (m, 4H), 5.03 (s, 2H), 2.45 (s, 3H), 2.40 (s, 3H). **^{13}C NMR** (100 MHz, DMSO- *d*6) *δ* 167.80, 156.32, 146.16, 141.06, 138.98, 138.16, 137.88, 137.19, 128.33, 126.54, 115.40 (d, *J* = 22.2 Hz), 113.92 (d, *J* = 7.7 Hz), 64.00, 20.26, 19.89. **HR-MS** (ESI): *m/z* calcd for $C_{18}H_{18}FN_4O_2^+$ ([M+H]$^+$) 341.1408, found 341.1408.

24 2-((6,7-*d*imethylquinoxalin-2-yl)oxy)-*N'*-(2-fluorophenyl)acetohydrazide

Yellow solid, yield: 41%, m.p. 201.2-202.0 °C. **^{1}H NMR** (400 MHz, DMSO- *d*6) *δ* 10.18 (d, *J* = 2.1 Hz, 1H), 8.58 (s, 1H), 7.63 (s, 1H), 7.04 (ddd, *J* = 12.1, 8.1, 1.3 Hz, 1H), 7.00 – 6.79 (m, 2H), 6.70 (td, *J* = 5.2, 2.5 Hz, 1H), 5.05 (s, 2H), 2.45 (s, 3H), 2.40 (s, 3H). **^{13}C NMR** (100 MHz, DMSO- *d*6) *δ* 167.86, 156.30, 151.79, 149.40, 141.04, 138.97, 138.16, 137.91, 137.19, 128.35, 126.54, 124.70, 119.09, 115.13, 114.24, 64.00, 20.31, 19.90. **HR-MS** (ESI): *m/z* calcd for $C_{18}H_{17}FN_4O_2^+$ ([M+H]$^+$) 341.1408, found 341.1011.

25 2-((6-chloroquinoxalin-2-yl)oxy)-*N'*-(2-fluorophenyl)acetohydrazide

Yellow solid, yield: 80%, m.p. 214.5-215.5 °C. **^{1}H NMR** (400 MHz, DMSO- *d*6) *δ* 10.22 (s, 1H), 8.74 (s, 1H), 8.09 (s, 1H), 7.97 – 7.67 (m, 3H), 7.04 (dd, *J* = 12.1, 8.0 Hz, 1H), 6.90 (d, *J* = 6.3 Hz, 2H), 6.81 – 6.45 (m, 1H), 5.10 (s, 2H). **^{13}C NMR** (100 MHz, DMSO- *d*6) *δ* 167.56, 156.86, 149.35, 141.74, 139.35, 138.38, 137.11, 131.67(d,

J = 15.1 Hz), 128.94, 128.02, 124.81, 119.17, 115.15, 114.13, 64.29. **HR-MS** (ESI): *m/z* calcd for $C_{16}H_{13}ClFN_4O_2^+$ ([M+H]$^+$) 347.0706, found 347.0707.

26 2-((6-chloroquinoxalin-2-yl)oxy)-*N'*-(4-fluorophenyl)acetohydrazide

Yellow solid, yield: 74%, m.p. 210.2-211.6 °C. **^{1}H NMR** (400 MHz, DMSO- *d*6) *δ* 10.15 (d, *J* = 2.5 Hz, 1H), 8.72 (s, 1H), 8.05 (d, *J* = 2.6 Hz, 2H), 7.97 – 7.76 (m, 2H), 7.75 – 7.61 (m, 1H), 7.11 (d, *J* = 8.5 Hz, 2H), 6.79 (d, *J* = 8.6 Hz, 2H), 5.10 (s, 2H). **^{13}C NMR** (100 MHz, DMSO- *d*6) *δ* 167.52, 156.89, 155.09, 146.11, 141.75, 139.34, 138.40, 131.57 (d, *J* = 15.1 Hz), 128.97, 128.01, 115.48 (d, *J* = 22.6 Hz), 113.84 (d, *J* = 7.8 Hz), 64.31. **HR-MS** (ESI): *m/z* calcd for $C_{16}H_{13}ClFN_4O_2^+$ ([M+H]$^+$) 347.0706, found 347.0709.

27 *N'*-(4-chlorophenyl)-2-((6-chloroquinoxalin-2-yl)oxy)acetohydrazide

Yellow solid, yield: 63%, m.p. 197.6-199.9 °C. **^{1}H NMR** (400 MHz, DMSO- *d*6) *δ* 10.18 (s, 1H), 8.73 (s, 1H), 8.07 (d, *J* = 18.3 Hz, 2H), 7.86 (s, 2H), 7.11 (d, *J* = 8.3 Hz, 2H), 6.76 (d, *J* = 8.4 Hz, 2H), 5.08 (s, 2H). **^{13}C NMR** (100 MHz, DMSO- *d*6) *δ* 167.54, 156.88, 148.55, 141.76, 139.36, 131.68, 131.50, 128.98, 128.84, 128.04, 122.28, 114.13, 64.30. **HR-MS** (ESI): *m/z* calcd for $C_{16}H_{13}Cl_2N_4O_2^+$ ([M+H]$^+$) 363.0410, found 363.0420.

28 *N'*-(4-bromophenyl)-2-((6-chloroquinoxalin-2-yl)oxy)acetohydrazide

Yellow solid, yield: 69%., m.p. 204.4-205.6 °C. **^{1}H NMR** (400 MHz, DMSO- *d*6) *δ* 10.16 (d, *J* = 2.2 Hz, 1H), 8.74 (s, 1H), 8.08 (dd, *J* = 19.0, 2.2 Hz, 2H), 7.98 – 7.81 (m, 2H), 7.23 (d, *J* = 8.4 Hz, 2H), 6.71 (d, *J* = 8.4 Hz, 2H), 5.08 (s, 2H). **^{13}C NMR** (100 MHz, DMSO- *d*6) *δ* 167.52, 156.88, 148.95, 141.77, 139.37, 138.39, 131.67, 131.51, 128.99, 128.05, 114.64, 109.78, 64.30. **HR-MS** (ESI): *m/z* calcd for $C_{16}H_{13}ClBrN_4O_2^+$ ([M+H]$^+$) 406.9905, found 406.9900.

29 *N'*-(2-bromophenyl)-2-((6-chloroquinoxalin-2-yl)oxy)acetohydrazide

Yellow solid, yield: 61%, m.p. 202.1-203.5 °C. **^{1}H NMR** (400 MHz, DMSO- *d*6) *δ* 10.35 (s, 1H), 8.75 (s, 1H), 8.10 (d, *J* = 2.1 Hz, 1H), 7.99 – 7.70 (m, 2H), 7.50 – 7.30 (m, 2H), 7.21 – 6.99 (m, 1H), 6.88 (dd, *J* = 8.1, 1.5 Hz, 1H), 6.66 (td, *J* = 7.6, 1.5 Hz, 1H), 5.11 (s, 2H). **^{13}C NMR** (100 MHz, DMSO- *d*6) *δ* 167.46, 156.86, 145.88, 141.76, 139.38, 138.38, 132.78, 131.70, 131.50, 128.97, 128.62, 128.04, 120.65, 113.78, 107.29, 64.32. **HR-MS** (ESI): *m/z* calcd for $C_{16}H_{13}ClBrN_4O_2^+$ ([M+H]$^+$) 406.9905, found

406.9901.

30 *N'*-(2-chlorophenyl)-2-((6-chloroquinoxalin-2-yl)oxy)acetohydrazide

Yellow solid, yield: 63%, m.p. 198.9-200.0 °C. **^{1}H NMR** (400 MHz, DMSO- *d*6) *δ* 10.38 (s, 1H), 8.75 (s, 1H), 8.10 (d, *J* = 2.1 Hz, 1H), 7.92 – 7.69 (m, 2H), 7.57 (s, 1H), 7.24 (d, *J* = 7.8 Hz, 1H), 7.06 (t, *J* = 7.7 Hz, 1H), 6.89 (d, *J* = 8.2 Hz, 1H), 6.70 (t, *J* = 7.6 Hz, 1H), 5.11 (s, 2H). **^{13}C NMR** (100 MHz, DMSO-d_6) *δ* 167.50, 156.88, 144.92, 141.77, 139.37, 138.39, 131.68, 131.50, 129.57, 128.98, 128.03, 119.92, 117.52, 113.51, 64.30. **HR-MS** (ESI): *m/z* calcd for $C_{16}H_{13}Cl_2N_4O_2^+$ ($[M+H]^+$) 363.0410, found 363.0415.

31 *N'*-(2-chlorophenyl)-2-((6,7-*d*ichloroquinoxalin-2-yl)oxy)acetohydrazide

Yellow solid, yield: 61%, m.p. 216.8-218.9°C. **^{1}H NMR** (400 MHz, DMSO-d_6) *δ* 10.39 (s, 1H), 8.74 (d, *J* = 17.2 Hz, 1H), 8.27 (d, *J* = 19.4 Hz, 1H), 8.14 – 7.68 (m, 1H), 7.56 (s, 1H), 7.31 (dd, *J* = 45.4, 7.8 Hz, 1H), 7.20 – 6.64 (m, 3H), 5.15 (d, *J* = 23.6 Hz, 2H). **^{13}C NMR** (100 MHz, DMSO- *d*6) *δ* 167.29, 144.92, 142.30, 137.97, 134.98, 133.60, 130.06, 129.98, 129.60, 128.04, 127.82, 119.98, 117.59, 113.54, 64.48. **HR-MS** (ESI): *m/z* calcd for $C_{16}H_{12}Cl_3N_4O_2^+$ ($[M+H]^+$) 397.0200, found 397.0202.

32 *N'*-(4-chlorophenyl)-2-((6,7-*d*ichloroquinoxalin-2-yl)oxy)acetohydrazide

Yellow solid, yield: 71%, m.p. 219.7-220.5 °C. **^{1}H NMR** (400 MHz, DMSO-d_6)δ 10.36 – 9.86 (m, 1H), 8.71 (d, *J* = 10.4 Hz, 1H), 8.20 (d, *J* = 26.9 Hz, 2H), 8.02 (d, *J* = 13.2 Hz, 2H), 7.89 (d, *J* = 21.3 Hz, 1H), 7.32 (d, *J* = 17.6 Hz, 1H), 7.10 (d, *J* = 8.3 Hz, 2H), 6.76 (d, *J* = 8.4 Hz, 2H), 5.13 (d, *J* = 38.6 Hz, 2H). **^{13}C NMR** (100 MHz, DMSO-d_6) *δ* 167.30, 157.30, 153.70, 148.52, 142.23, 138.85, 137.92, 133.60, 129.99, 128.79, 127.98, 122.35, 114.21, 64.46. **HR-MS** (ESI): *m/z* calcd for $C_{16}H_{12}Cl_3N_4O_2^+$ ($[M+H]^+$) 397.0200, found 397.0202.

33 *N'*-(4-bromophenyl)-2-((6,7-*d*ichloroquinoxalin-2-yl)oxy)acetohydrazide

Yellow solid, yield: 74%, m.p. 213.7-215.3 °C. **^{1}H NMR** (400 MHz, DMSO-d_6) *δ* 10.19 (s, 1H), 8.74 (d, *J* = 1.9 Hz, 1H), 8.57 – 8.18 (m, 1H), 8.10 – 8.02 (m, 2H), 7.21 (s, 1H), 6.72 (d, *J* = 8.5 Hz, 2H), 5.13 (d, *J* = 36.3 Hz, 2H). **^{13}C NMR** (100 MHz, DMSO-d_6) *δ* 167.31, 157.32, 148.95, 142.25, 133.61, 131.64, 130.01, 128.01, 115.14, 114.73, 109.84, 64.45. **HR-MS** (ESI): *m/z* calcd for $C_{16}H_{12}Cl_2BrN_4O_2^+$ ($[M+H]^+$) 440.9515, found 440.9520.

34 *N'*-(3-bromophenyl)-2-((6,7-*d*ichloroquinoxalin-2-yl)oxy)acetohydrazide

Yellow solid, yield: 87%, m.p. 205.1-206.8 °C. **^{1}H NMR** (400 MHz, DMSO-d_6) δ 10.19 (d, J = 2.1 Hz, 1H), 8.76 (s, 1H), 8.30 (s, 1H), 8.15 (d, J = 2.1 Hz, 1H), 8.07 (s, 1H), 7.02 (t, J = 8.0 Hz, 1H), 6.89 – 6.14 (m, 3H), 5.10 (s, 2H). **^{13}C NMR** (100 MHz, DMSO-d_6) δ 167.39, 157.41, 151.29, 142.35, 138.87, 138.00, 133.62, 130.99, 130.03, 128.32, 122.50, 121.35, 114.42, 111.90, 64.46. **HR-MS** (ESI): *m/z* calcd for $C_{16}H_{12}Cl_2BrN_4O_2^+$ ([M+H]$^+$) 440.9515, found 440.9521.

35 2-((6,7-*d*ichloroquinoxalin-2-yl)oxy)-*N'*-(2-fluorophenyl)acetohydrazide

Yellow solid, yield: 77%, m.p. 204.3-206.0 °C. **^{1}H NMR** (400 MHz, DMSO-d_6) δ 10.22 (d, J = 2.0 Hz, 1H), 8.76 (s, 1H), 8.26 (d, J = 20.5 Hz, 1H), 8.04 (s, 1H), 7.82 (s, 1H), 7.21 – 7.04 (m, 1H), 6.89 (dd, J = 5.9, 2.8 Hz, 1H), 6.71 (ddd, J = 8.8, 5.8, 3.0 Hz, 1H), 5.10 (s, 2H). **^{13}C NMR** (100 MHz, DMSO-d_6) δ 167.35, 157.35, 142.28, 138.89, 137.01, 133.59, 130.01 (d, J = 7.0 Hz), 128.02, 124.62, 119.19, 115.38, 115.20, 114.16, 64.47. **HR-MS** (ESI): *m/z* calcd for $C_{16}H_{12}FCl_2N_4O_2^+$ ([M+H]$^+$) 381.0316, found 381.0320.

36 2-((6,7-*d*ichloroquinoxalin-2-yl)oxy)-*N'*-(4-fluorophenyl)acetohydrazide

Yellow solid, yield: 71%, m.p. 215.8-217.6 °C. **^{1}H NMR** (400 MHz, DMSO-d_6) δ 10.22 (d, J = 2.8 Hz, 1H), 8.74 (s, 1H), 8.26 (s, 1H), 8.10 – 7.60 (m, 2H), 7.04 – 6.35 (m, 4H), 5.08 (s, 2H). **^{13}C NMR** (100 MHz, DMSO-d_6) δ 167.28, 157.35, 146.09, 142.27, 138.89, 137.92, 133.57, 129.98 (d, J = 6.2 Hz), 128.00, 115.52, 115.30, 113.97, 113.89, 64.47. **HR-MS** (ESI): *m/z* calcd for $C_{16}H_{12}FCl_2N_4O_2^+$ ([M+H]$^+$) 381.0316, found 381.0323.

4. NMR spectra of target compounds

In addition, it was found in the literature that the structure of these hydrazides undergoes isomerization (Scheme 2). From the ^{1}H NMR of compound 3, two singlets were found at 9.56 ppm and 8.35 ppm, belonging to the OH and =NNH hydrogen, respectively. The proportion of enols in all target compounds is 10-18%.

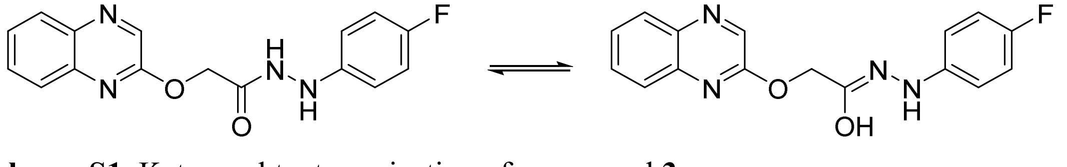

Scheme S1. Keto-enol tautomerization of compound **3**.

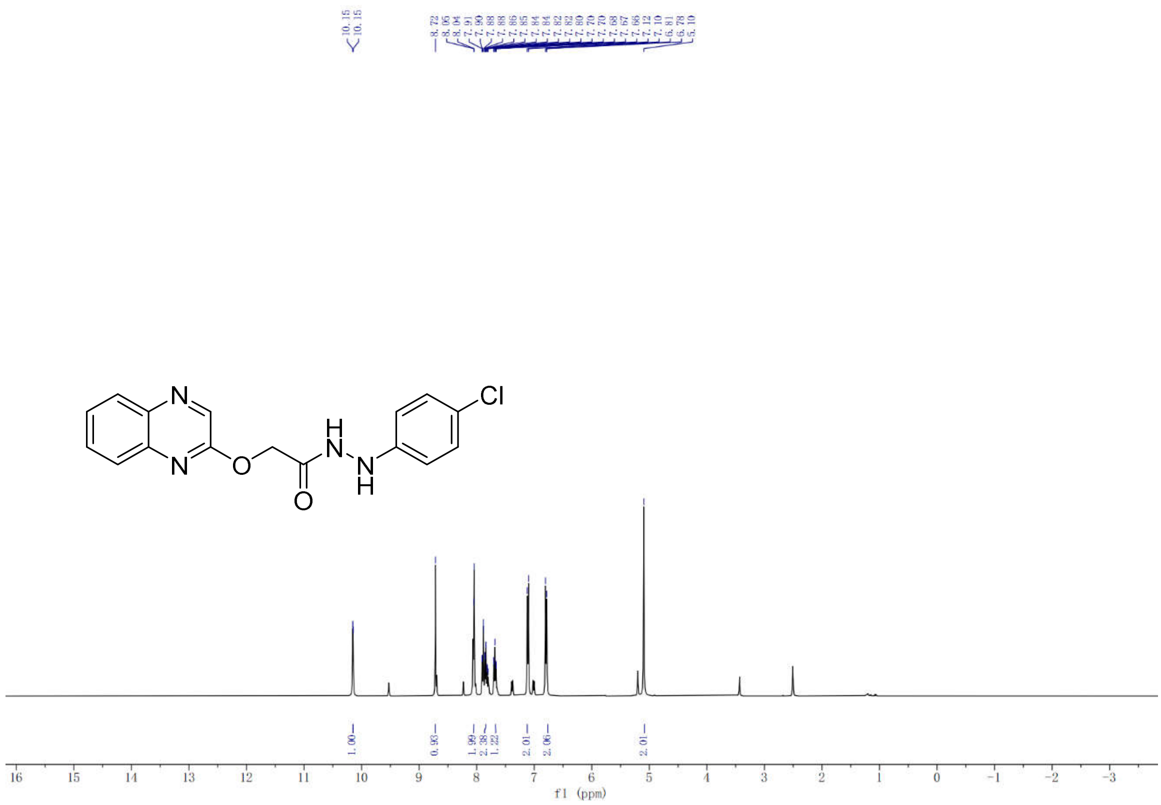

10.15
10.15
8.72
8.05
8.04
7.91
7.90
7.88
7.88
7.86
7.85
7.84
7.84
7.82
7.82
7.80
7.70
7.70
7.68
7.67
7.66
7.12
7.10
6.81
6.78
5.10
Cl
N
N
O
H
N
N
H
O
1.00
0.93
1.99
2.38
1.22
2.01
2.06
2.01
f1 (ppm)

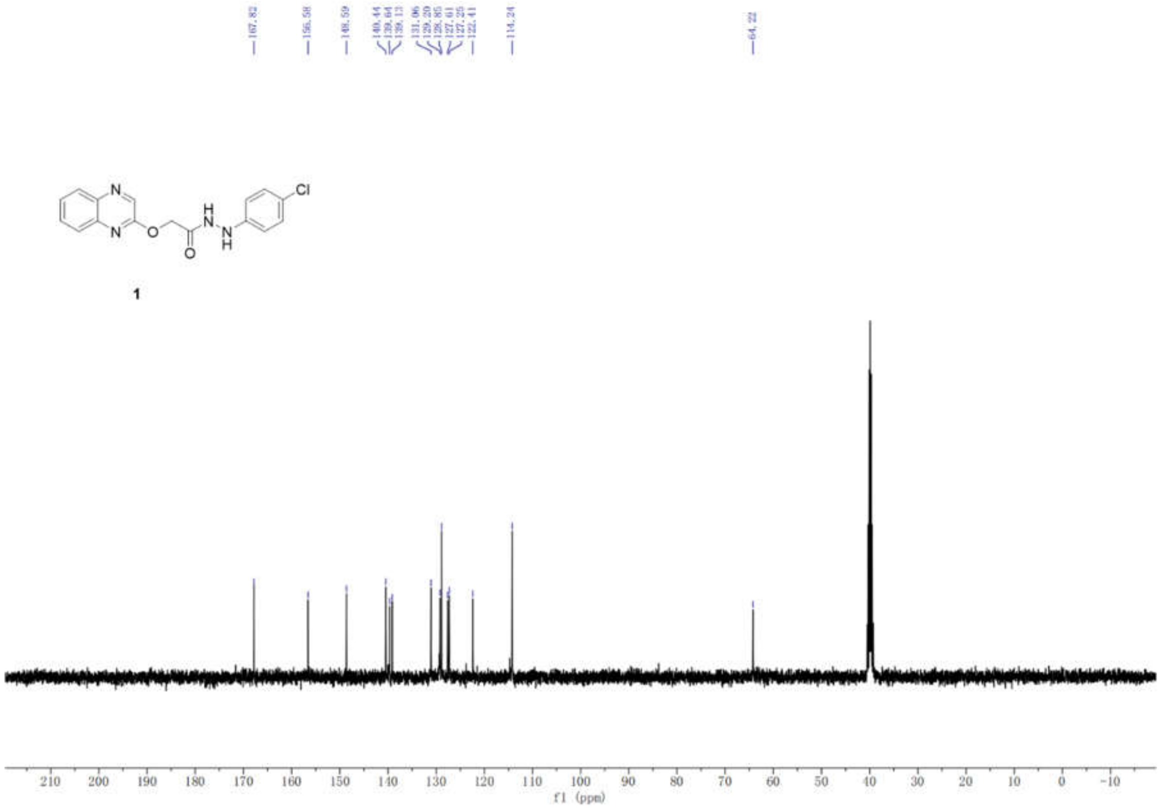

156.58
148.59
140.44
139.64
139.13
131.06
129.20
128.85
127.61
127.25
122.41
114.24
64.22
Cl
1
f1 (ppm)

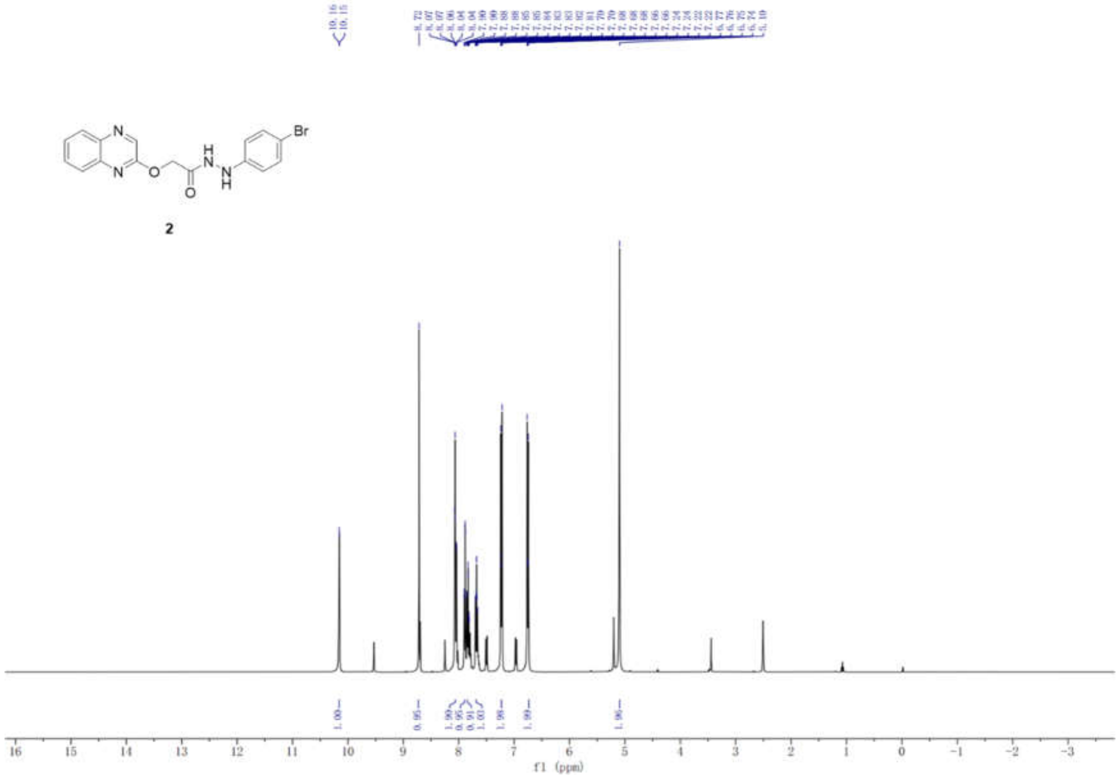
Br
2
f1 (ppm)

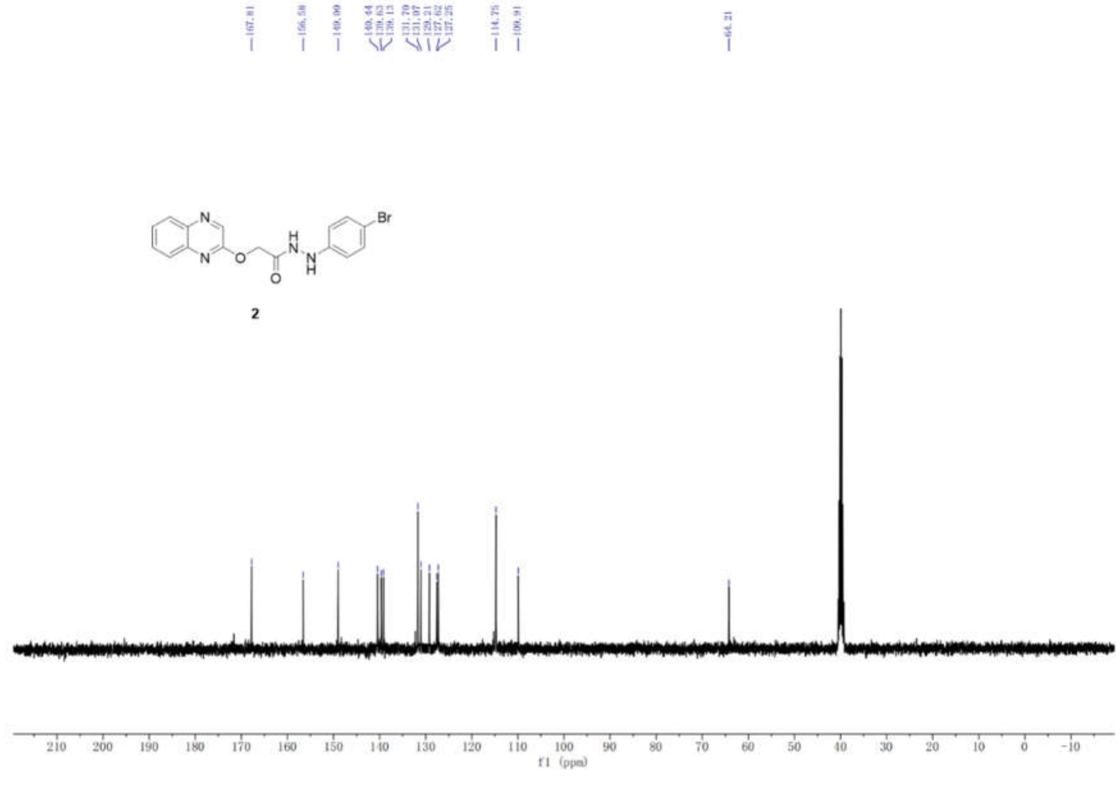
Br
2
f1 (ppm)

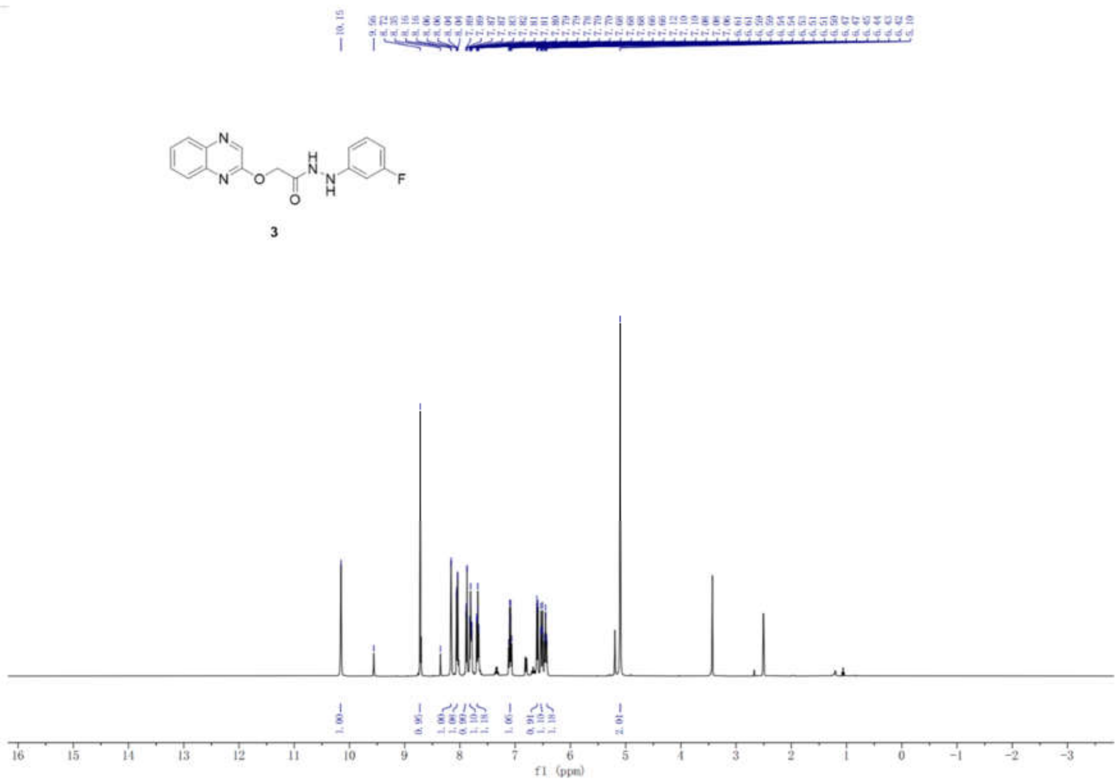
3
1.00
0.96
1.00
1.08
0.99
1.10
1.18
1.05
0.91
1.10
1.18
2.01
f1 (ppm)

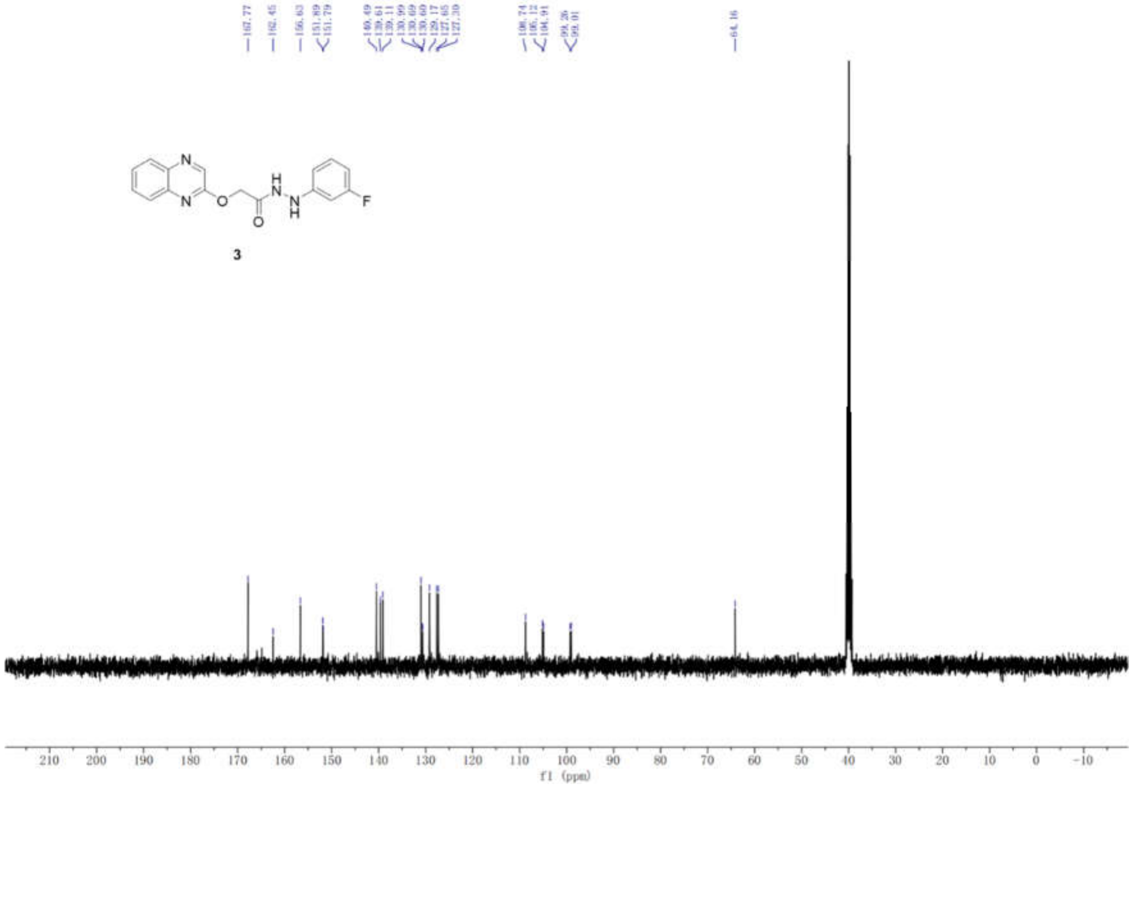
167.77
162.45
156.63
151.89
151.79
140.49
139.61
139.11
130.99
130.69
130.60
129.17
127.65
127.30
108.74
105.12
104.91
99.26
99.01
64.16
3
f1 (ppm)

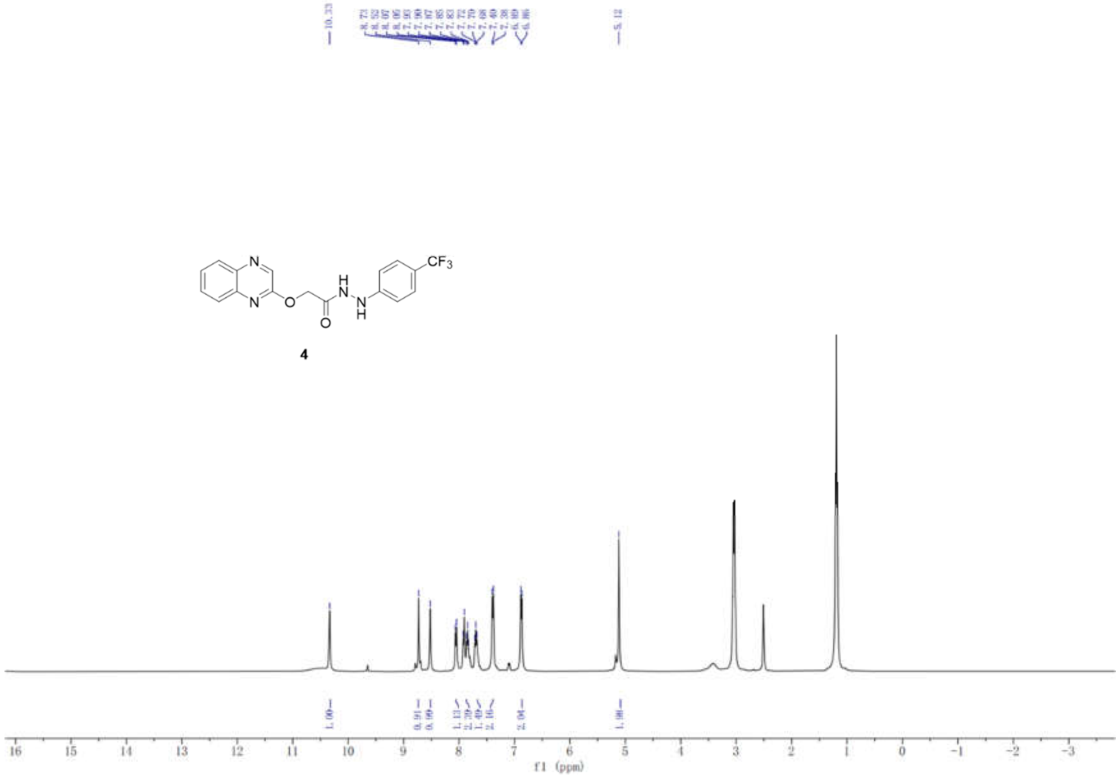
10.33
8.73 8.52 8.07 8.06 7.93 7.90 7.87 7.85 7.83 7.72 7.70 7.68 7.40 7.38 6.89 6.86
5.12
CF3
4
1.00 0.91 0.99 1.13 2.39 1.49 2.16 2.04 1.98
f1 (ppm)

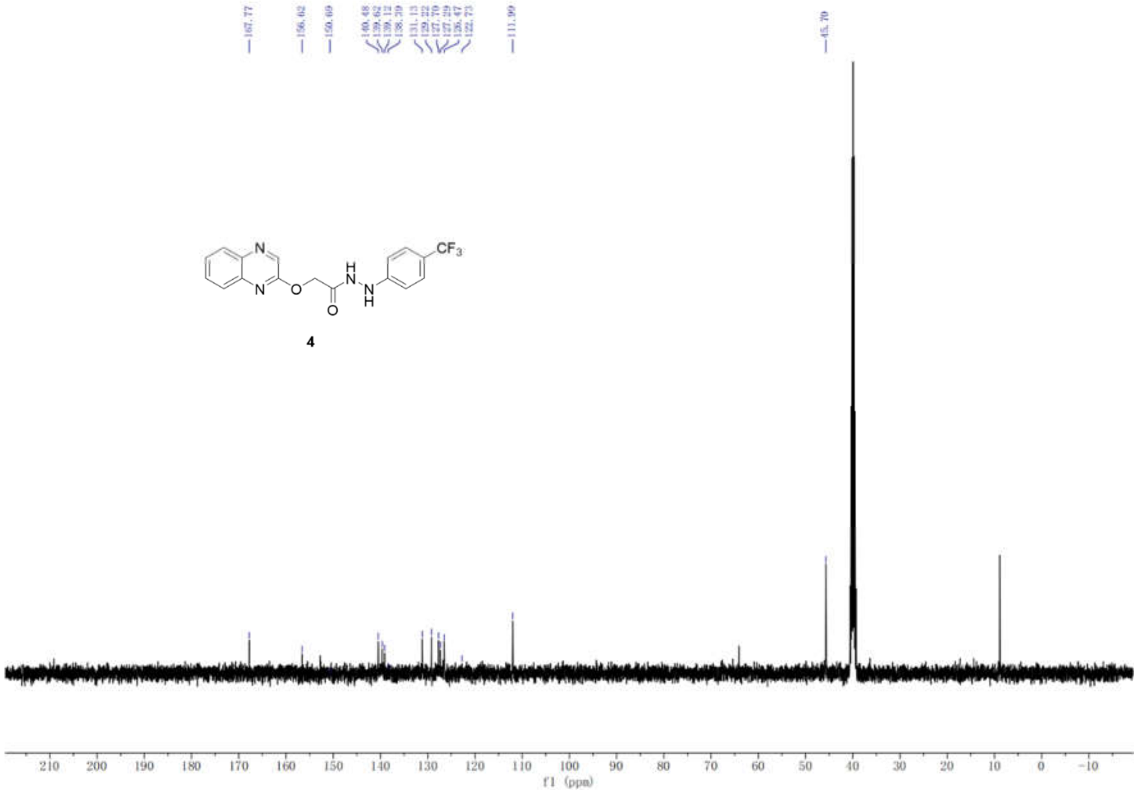
167.77
156.62
150.69
140.48 139.62 139.12 138.39 131.13 129.22 127.70 127.29 126.47 122.73
111.99
45.70
CF3
4
f1 (ppm)

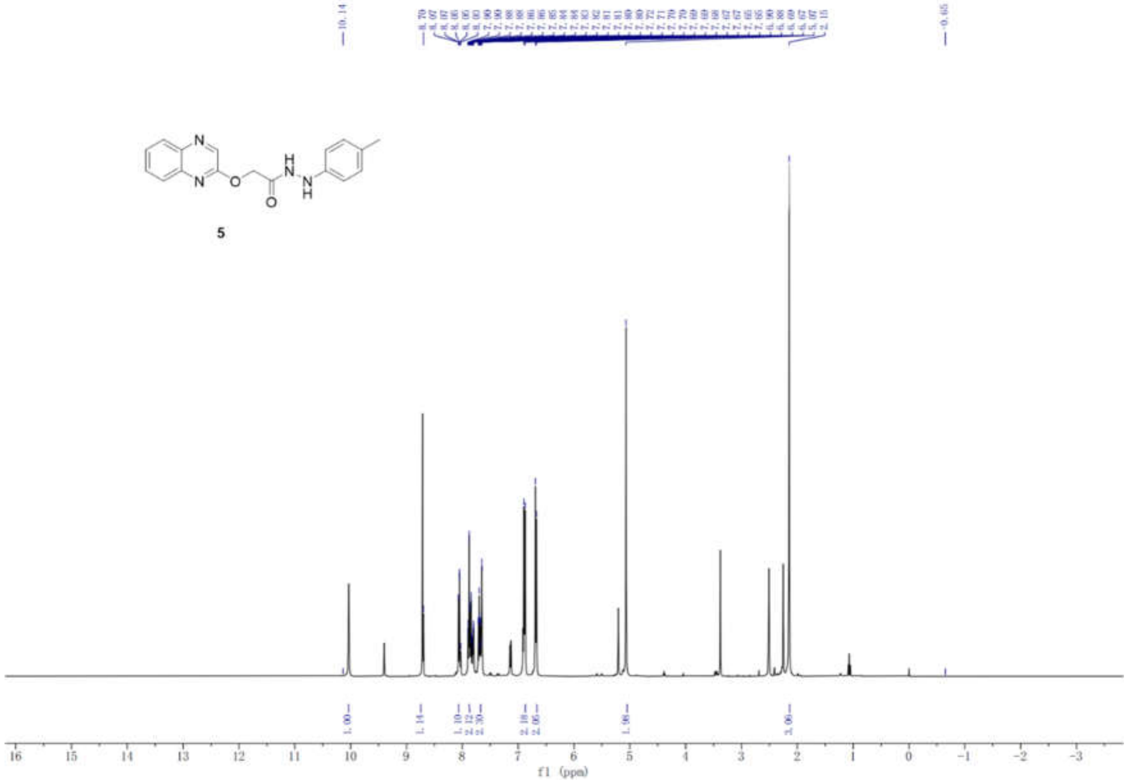

5

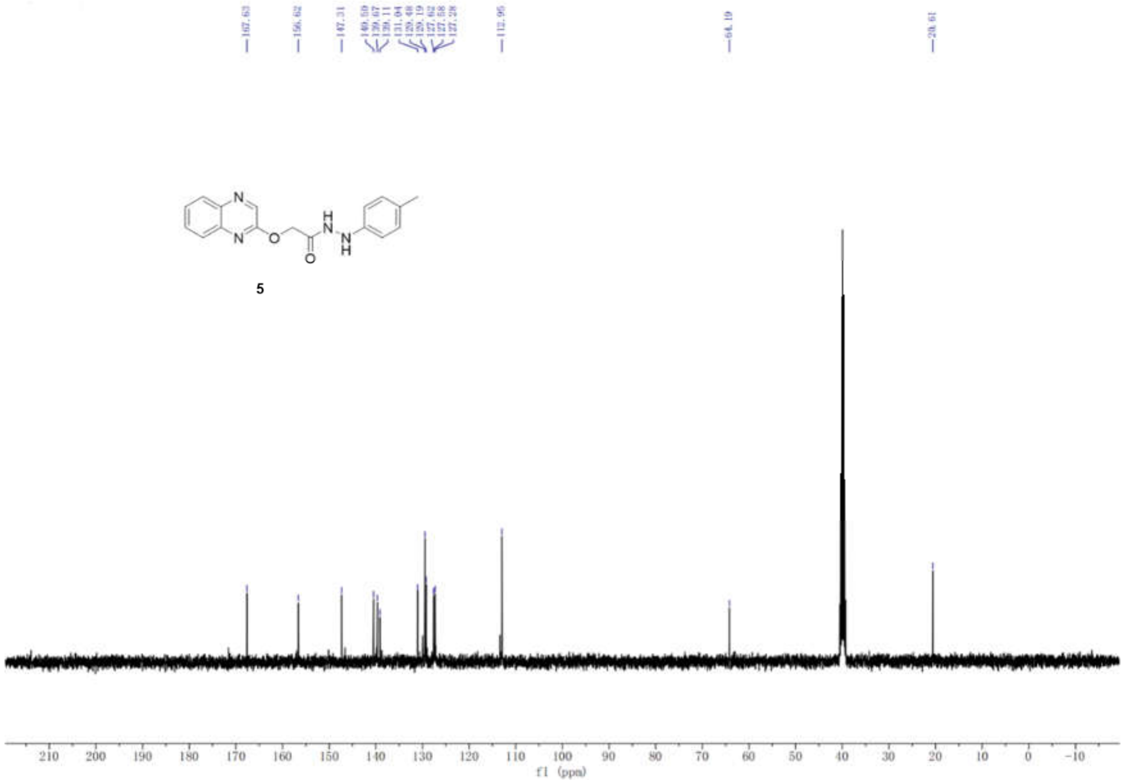

5

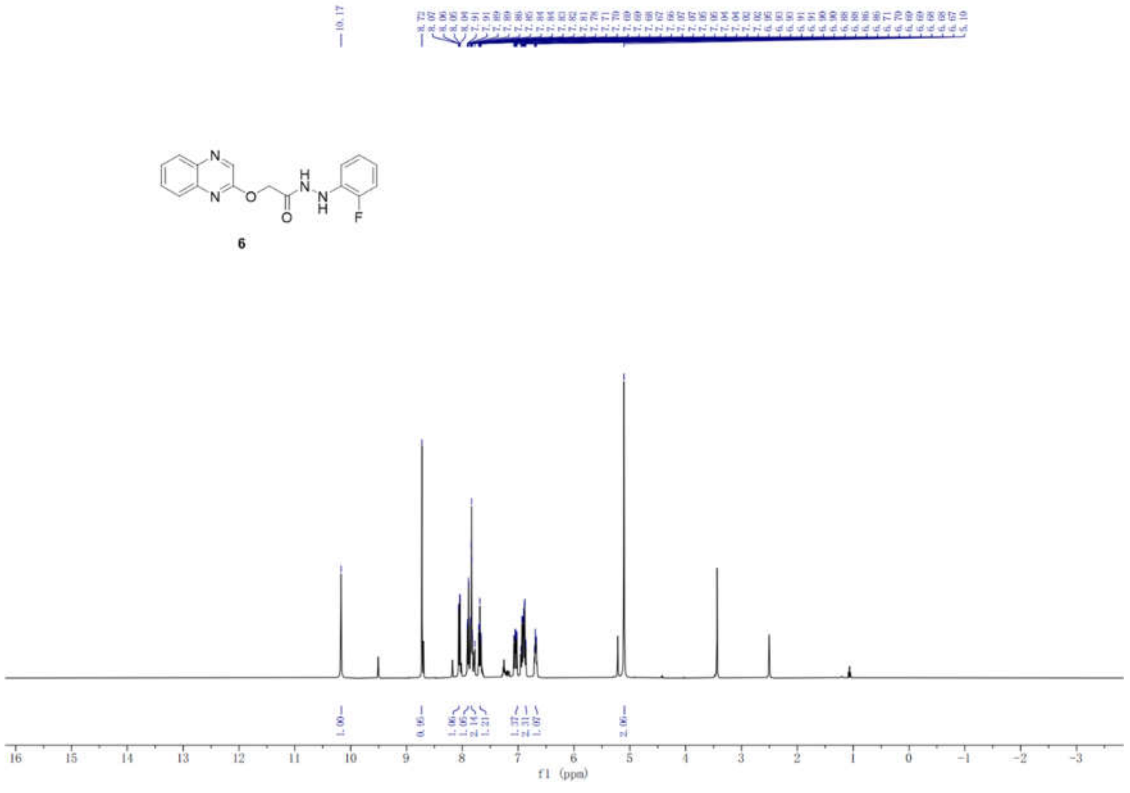

6

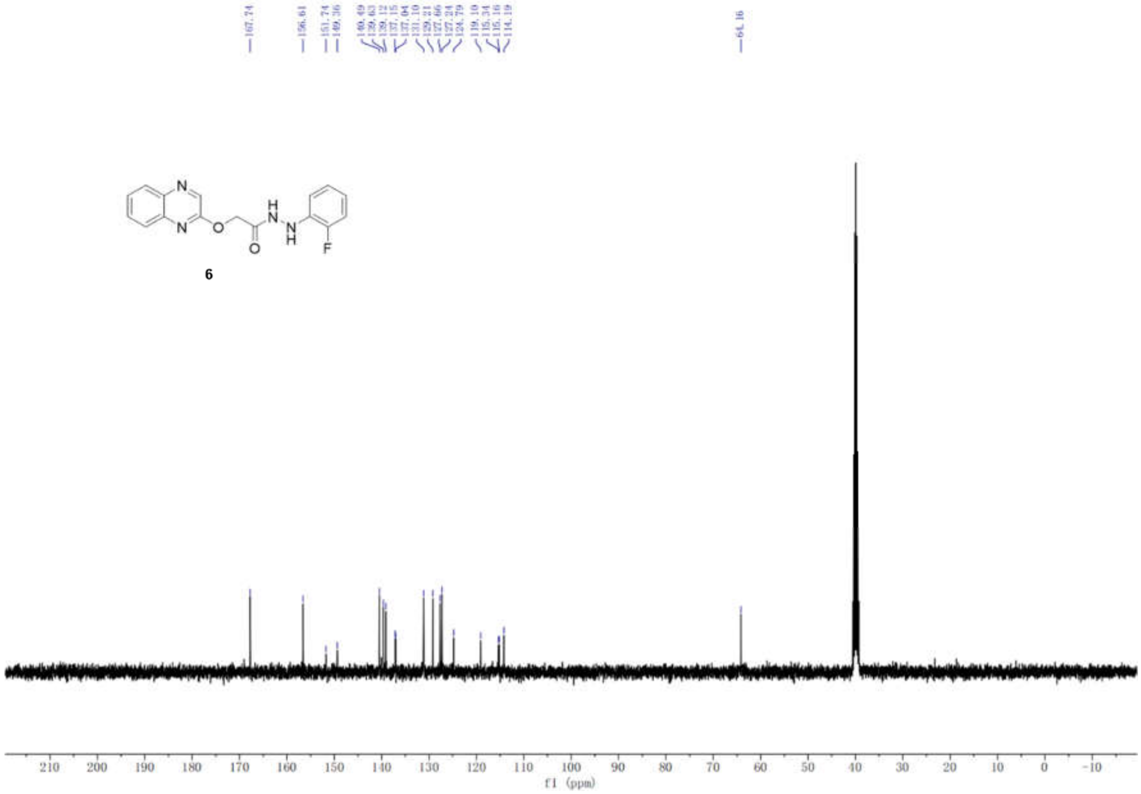

6

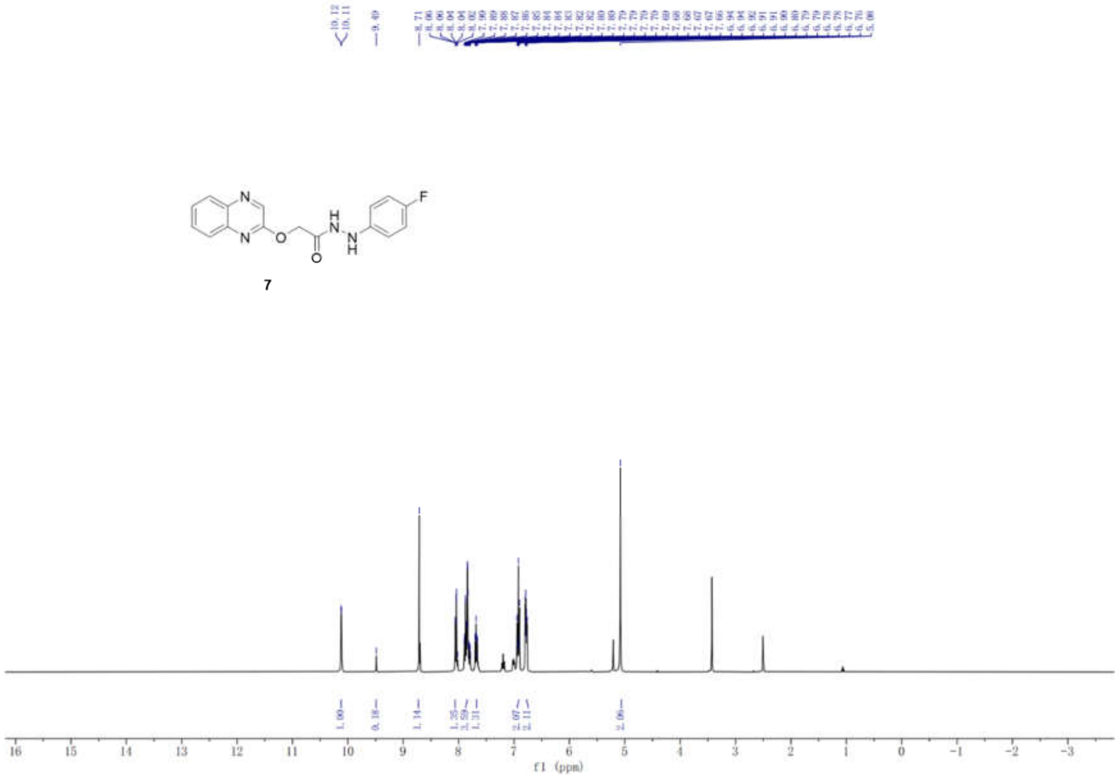

7

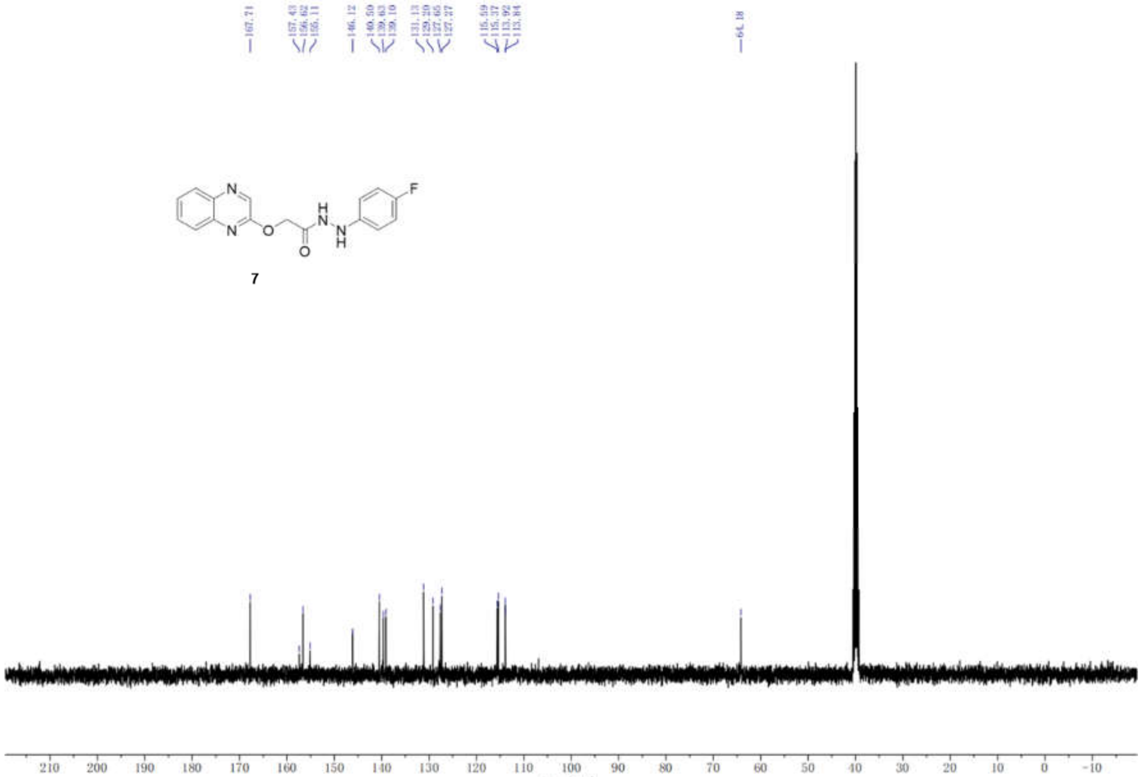

7

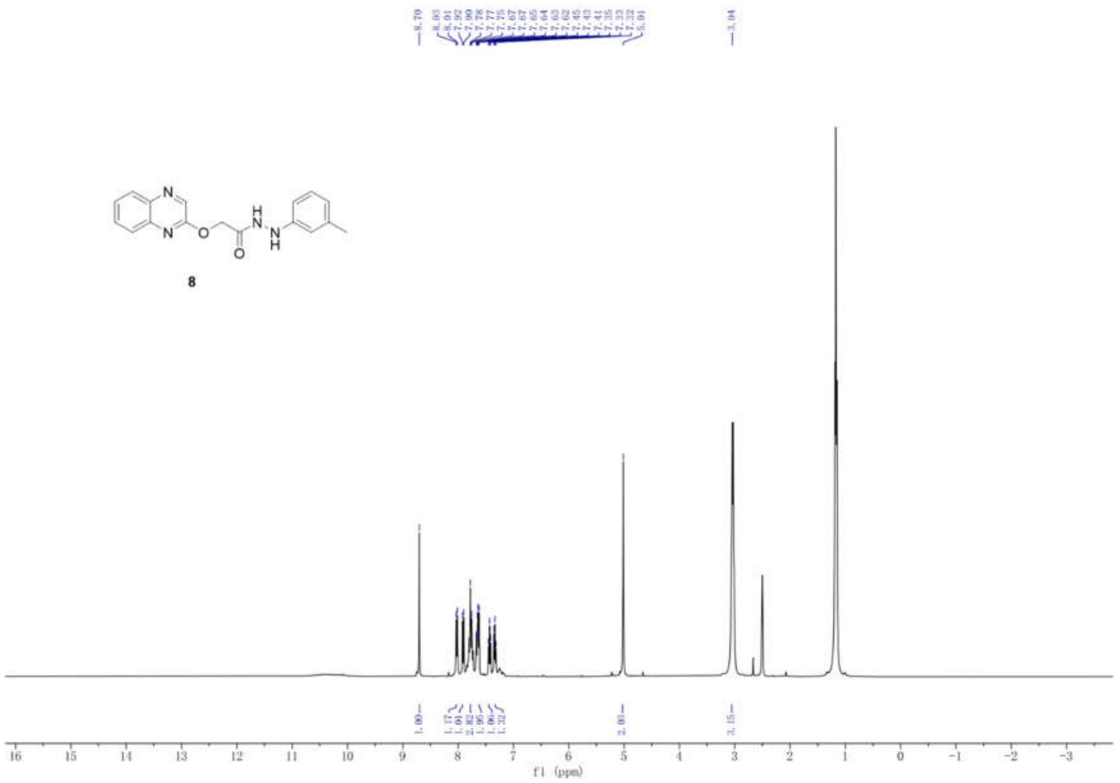
8
f1 (ppm)

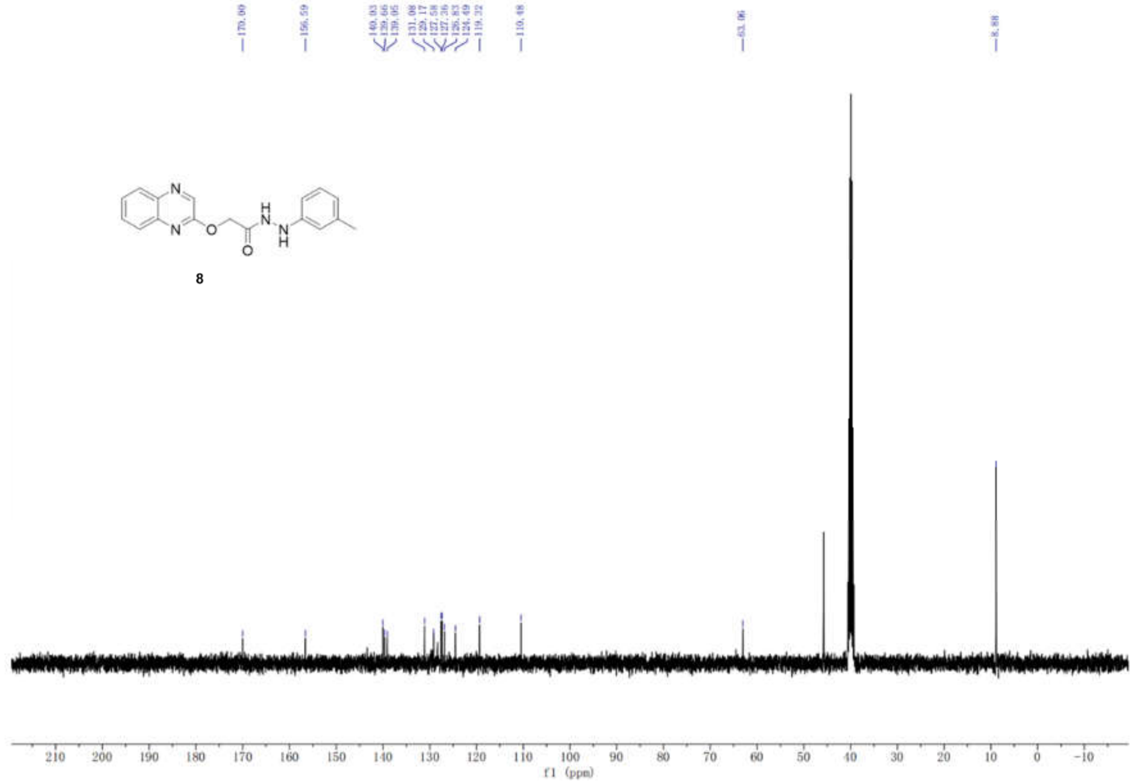
8
f1 (ppm)

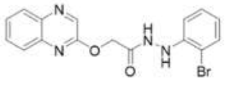

9

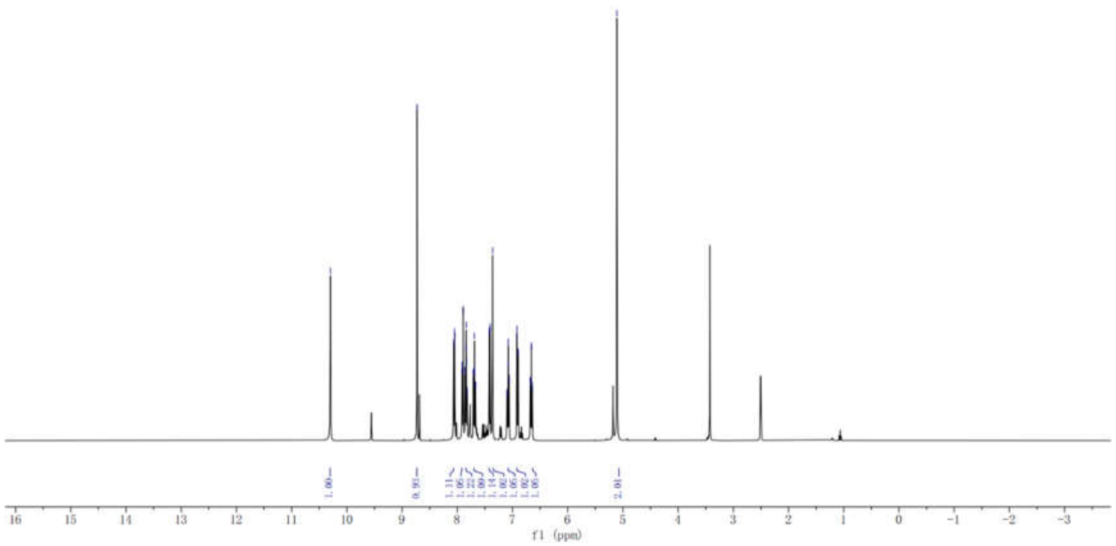

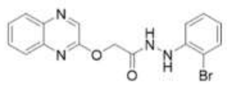

9

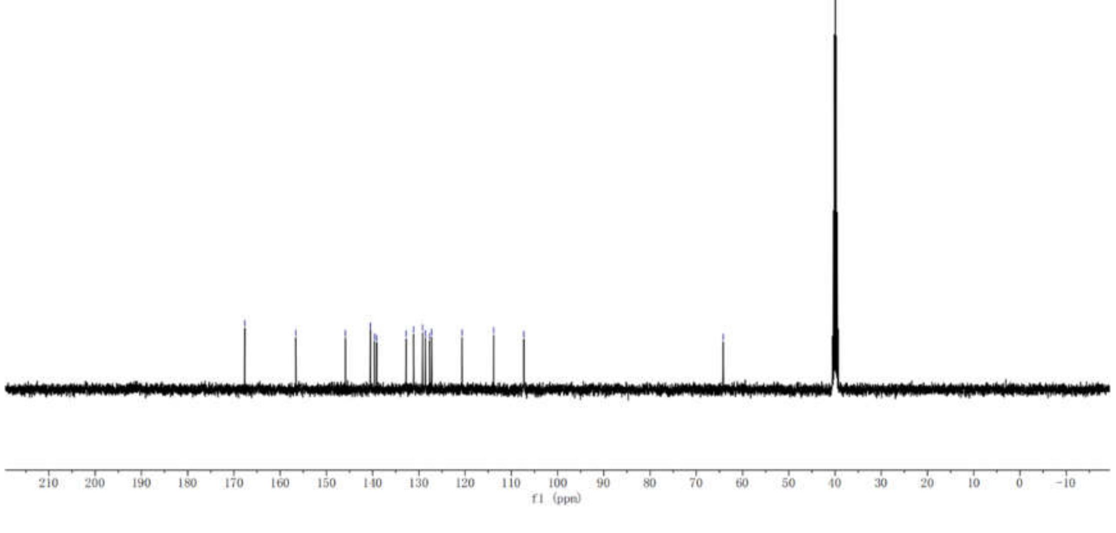

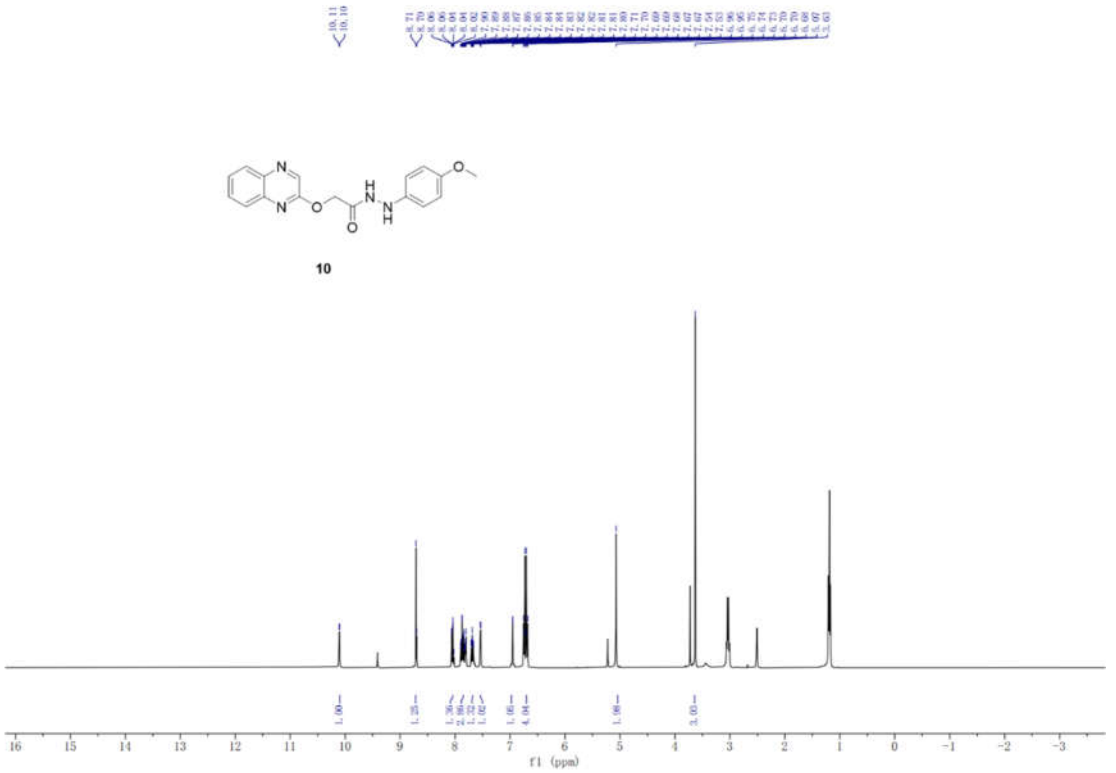
10
f1 (ppm)

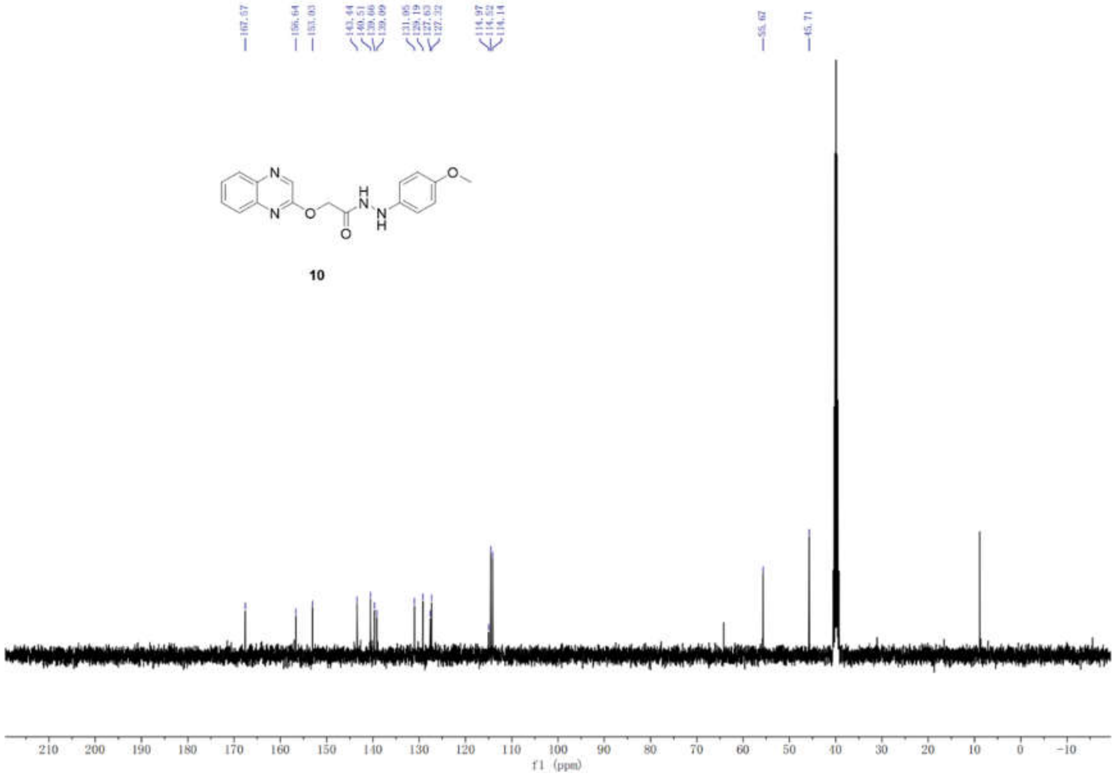
10
f1 (ppm)

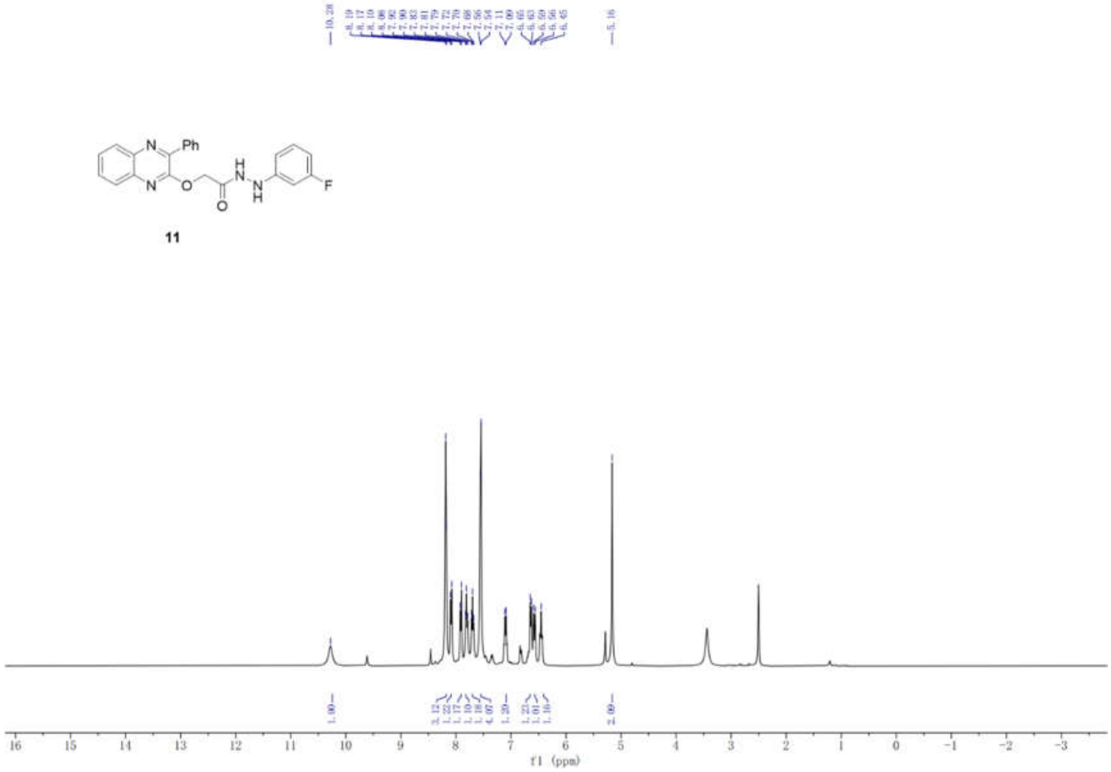

11

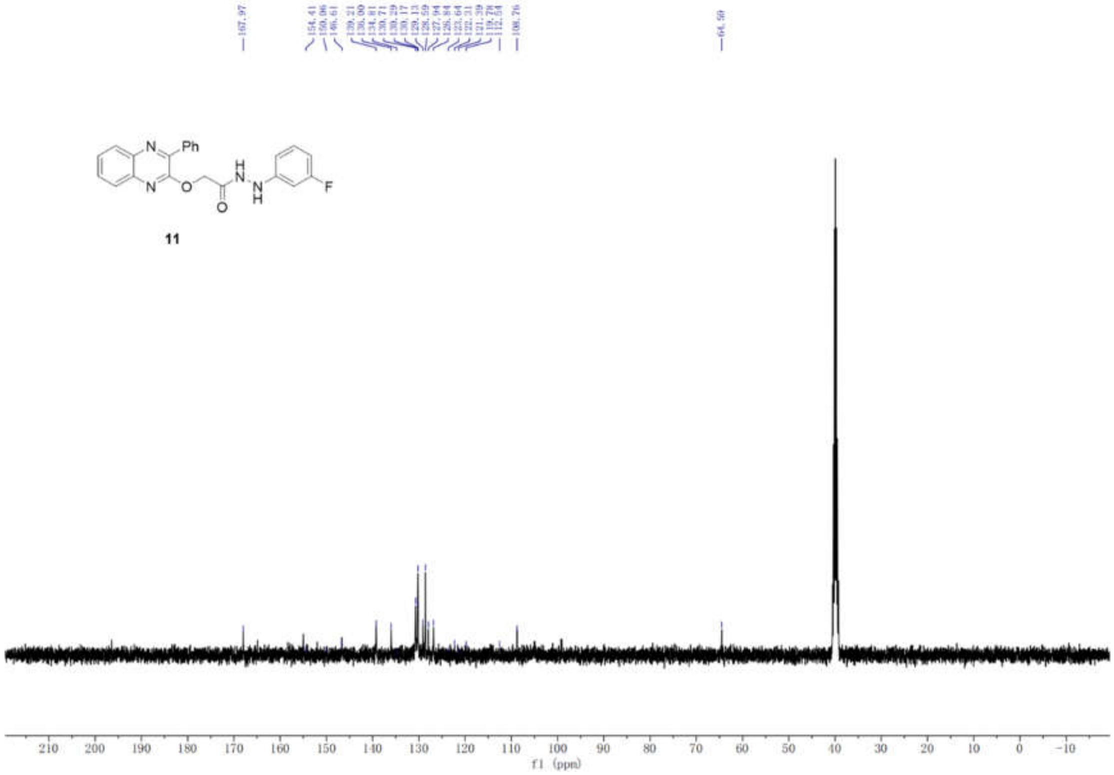

11

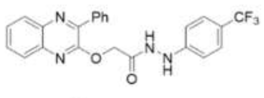

12

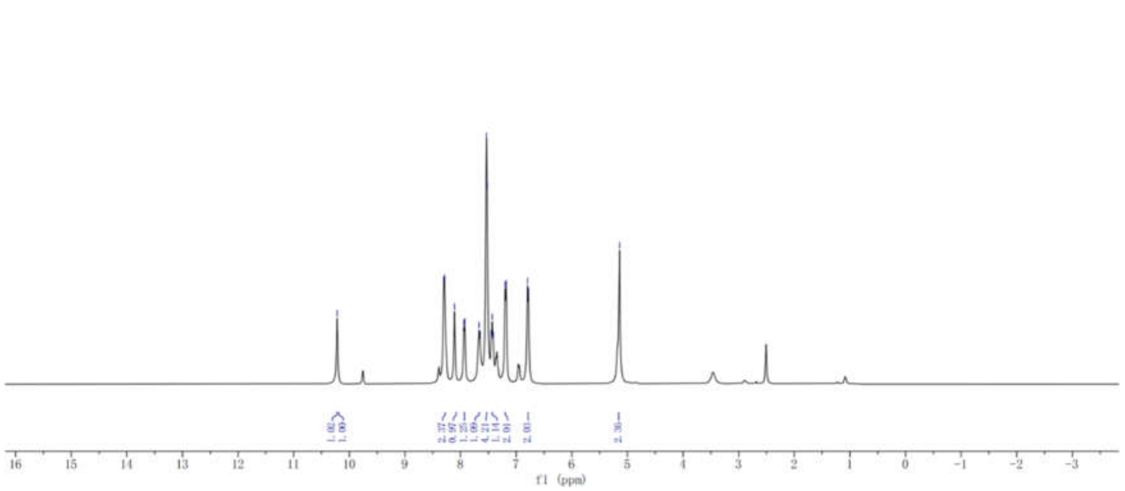
10.22
8.30
8.29
8.11
7.94
7.92
7.67
7.65
7.54
7.52
7.45
7.43
7.41
7.20
7.18
6.80
6.78
5.14
1.02
1.09
2.37
0.97
2.25
1.09
4.21
1.14
2.04
2.00
2.36
16 15 14 13 12 11 10 9 8 7 6 5 4 3 2 1 0 -1 -2 -3
f1 (ppm)

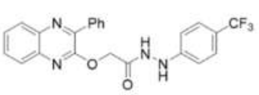

12

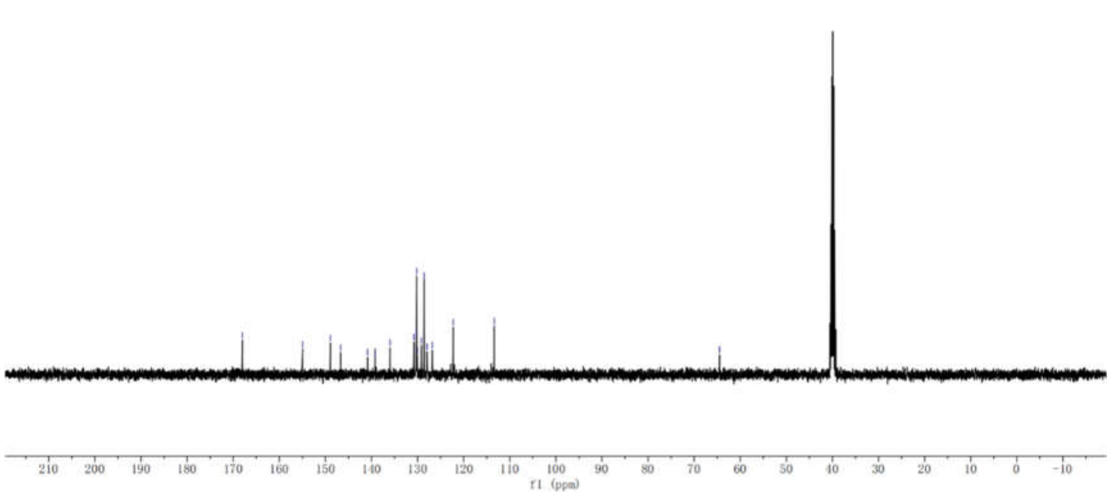
168.03
154.94
148.94
146.67
140.86
139.20
135.99
130.78
130.28
130.17
128.97
128.19
128.58
127.94
126.79
122.27
113.37
64.50
210 200 190 180 170 160 150 140 130 120 110 100 90 80 70 60 50 40 30 20 10 0 -10
f1 (ppm)

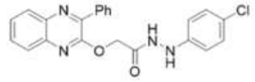

13

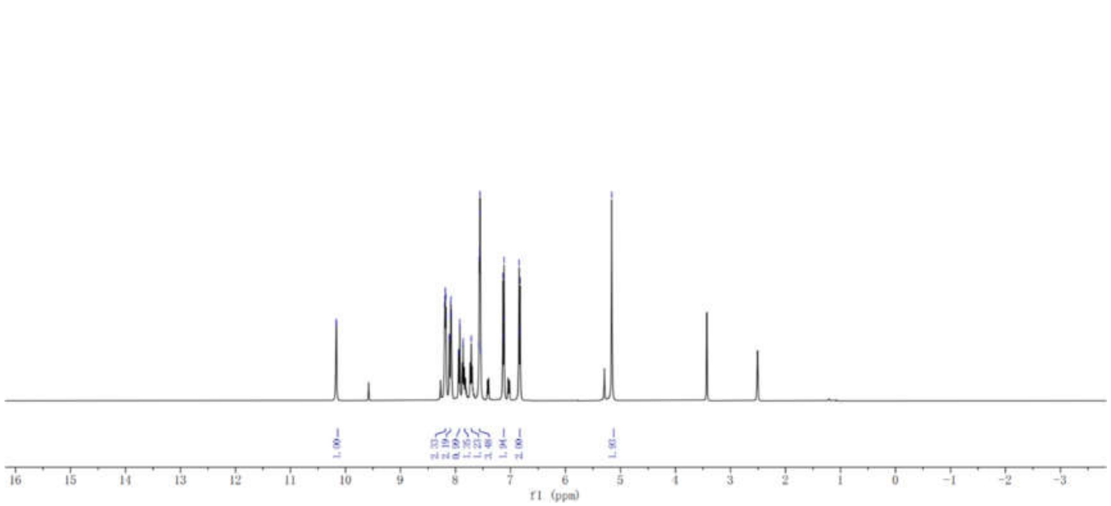

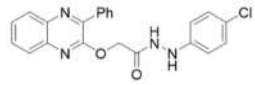

13

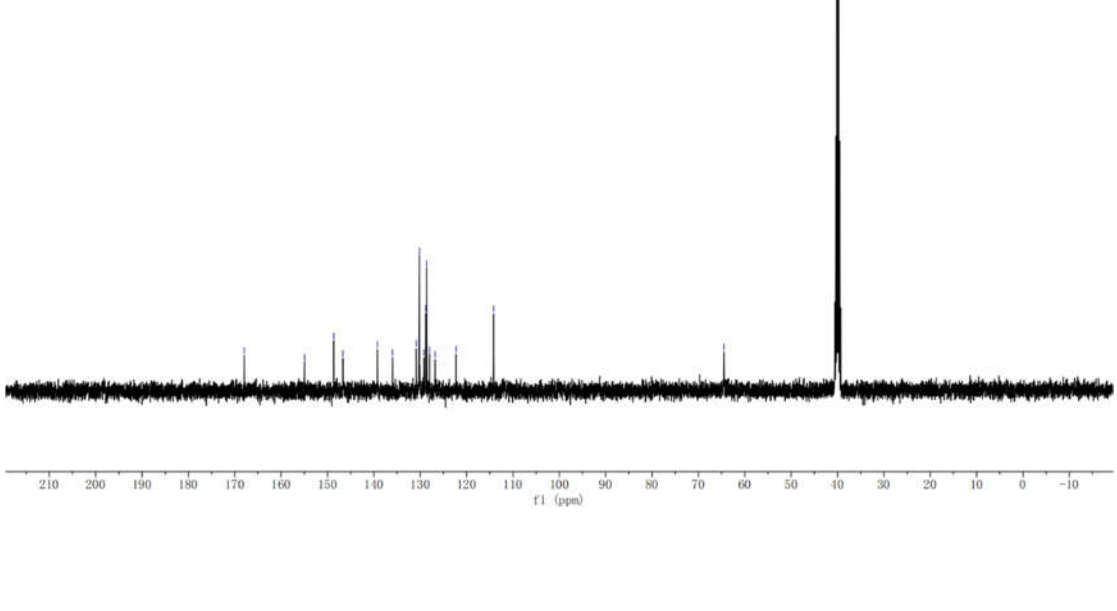

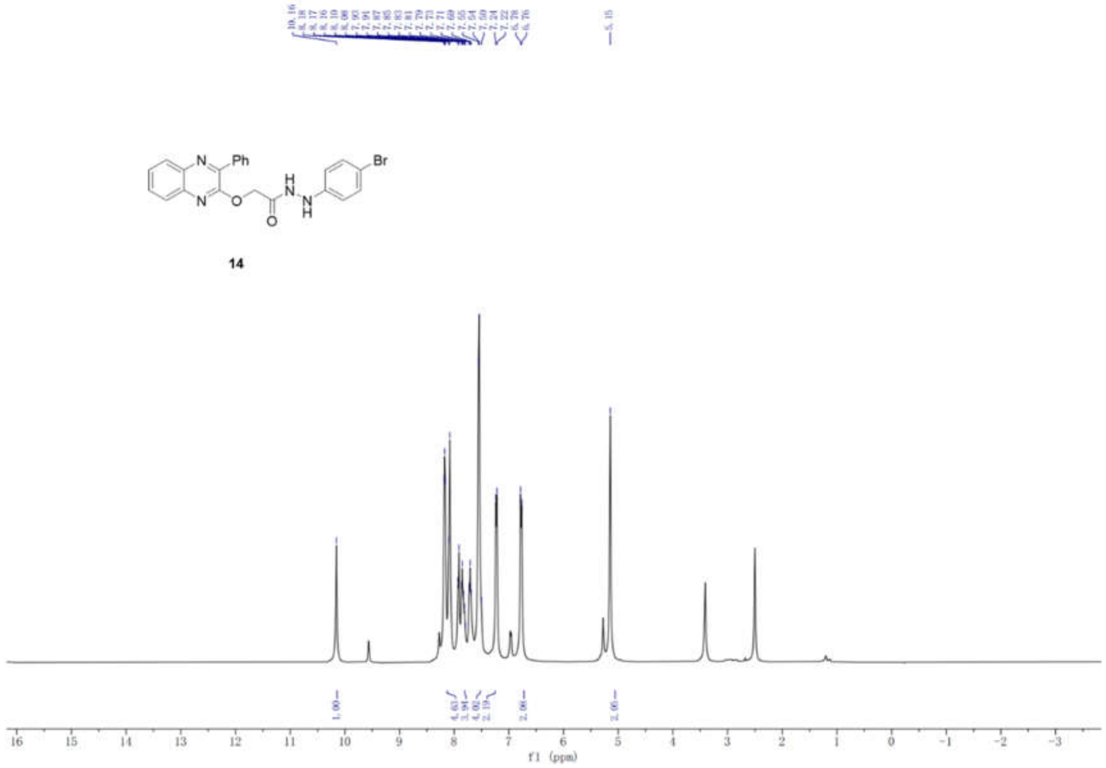

14

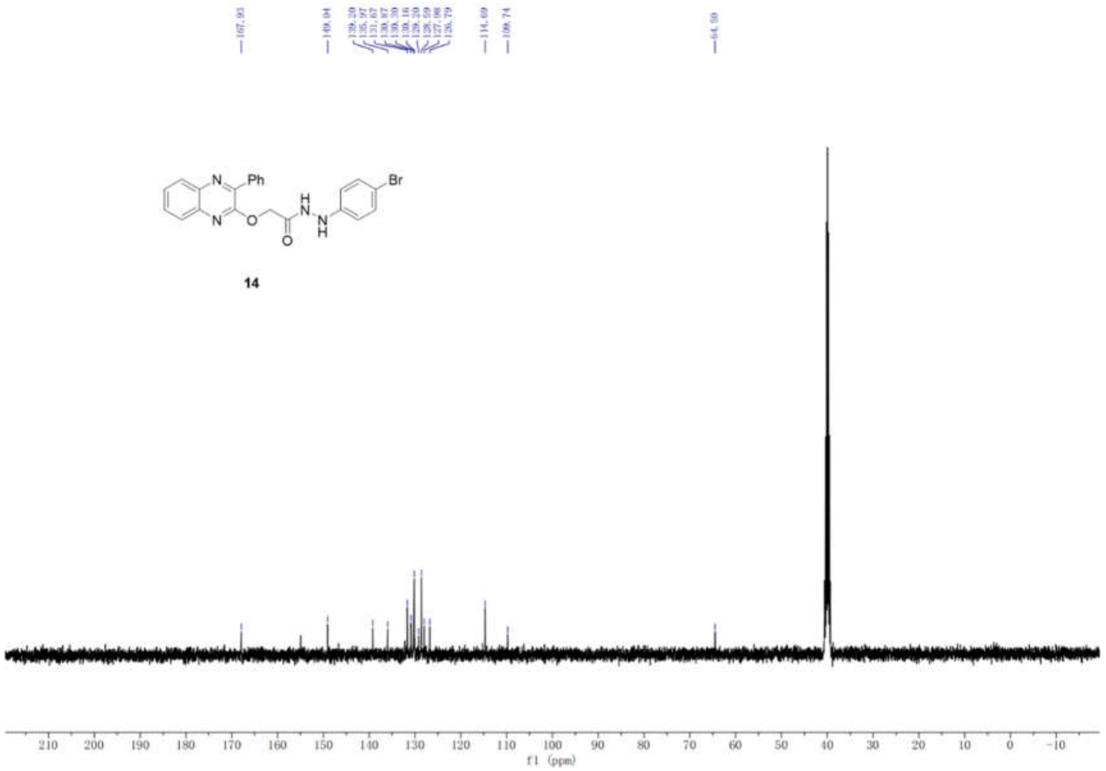

14

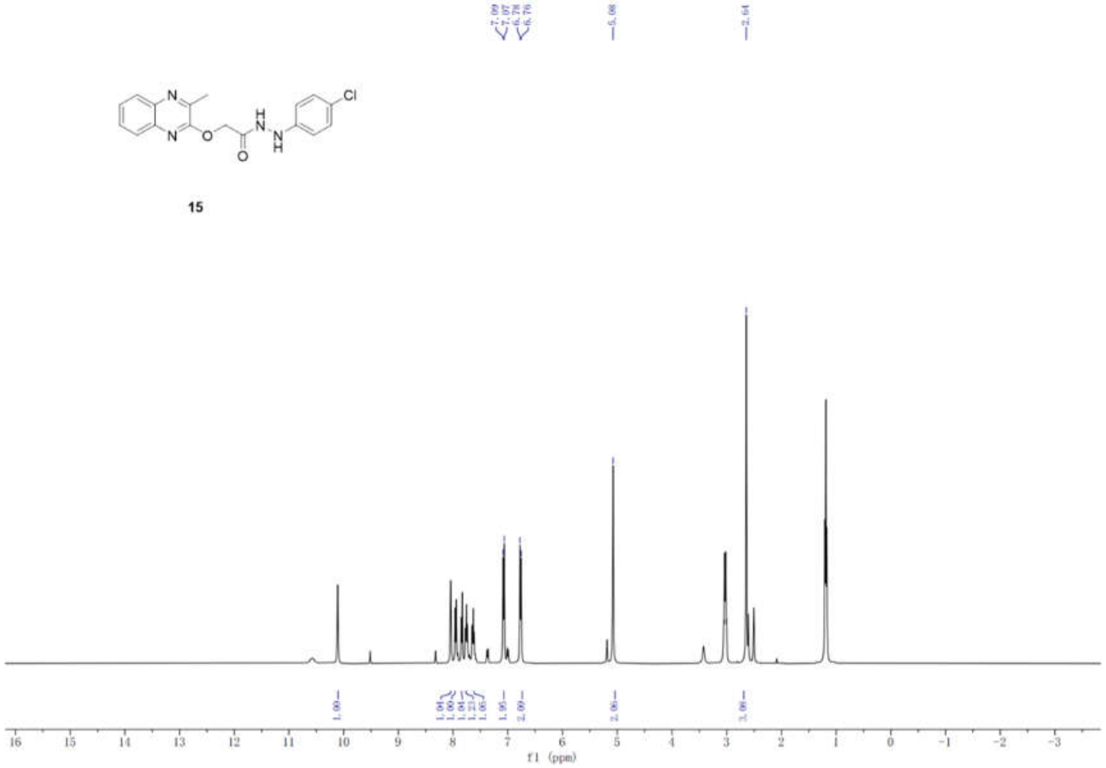
Cl
N
H
O
7.09
7.07
6.78
6.76
5.08
2.64
1.00
1.95
2.09
2.06
3.08
f1 (ppm)

15

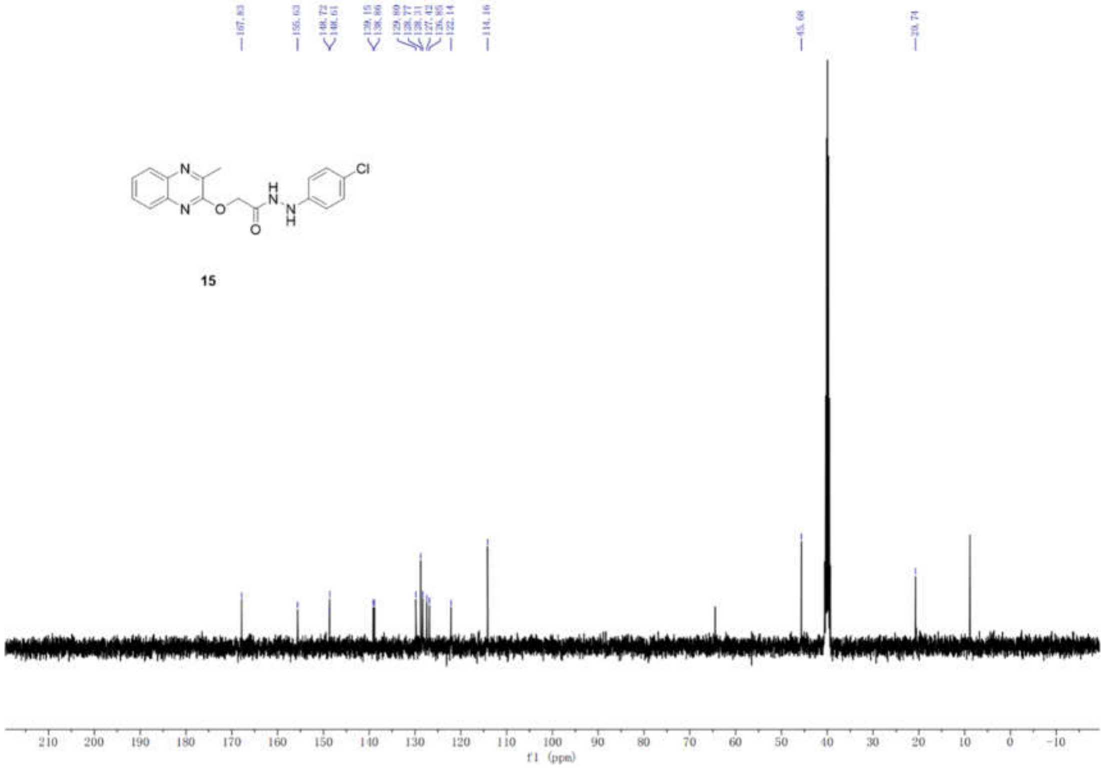
Cl
N
H
O
167.83
155.63
148.72
148.61
139.15
138.86
129.80
128.77
128.31
127.42
126.85
122.14
114.16
45.68
20.74
f1 (ppm)

15

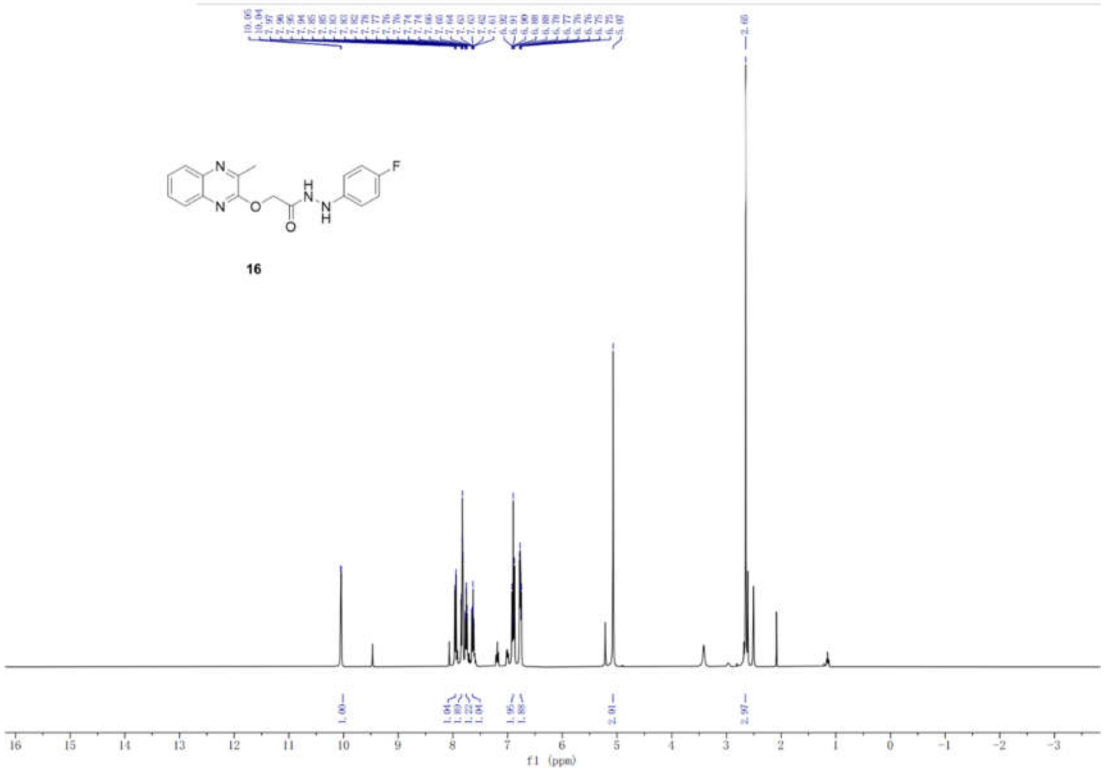

2.65
16
1.00
1.04
1.89
1.22
1.04
1.95
1.88
2.01
2.97
f1 (ppm)

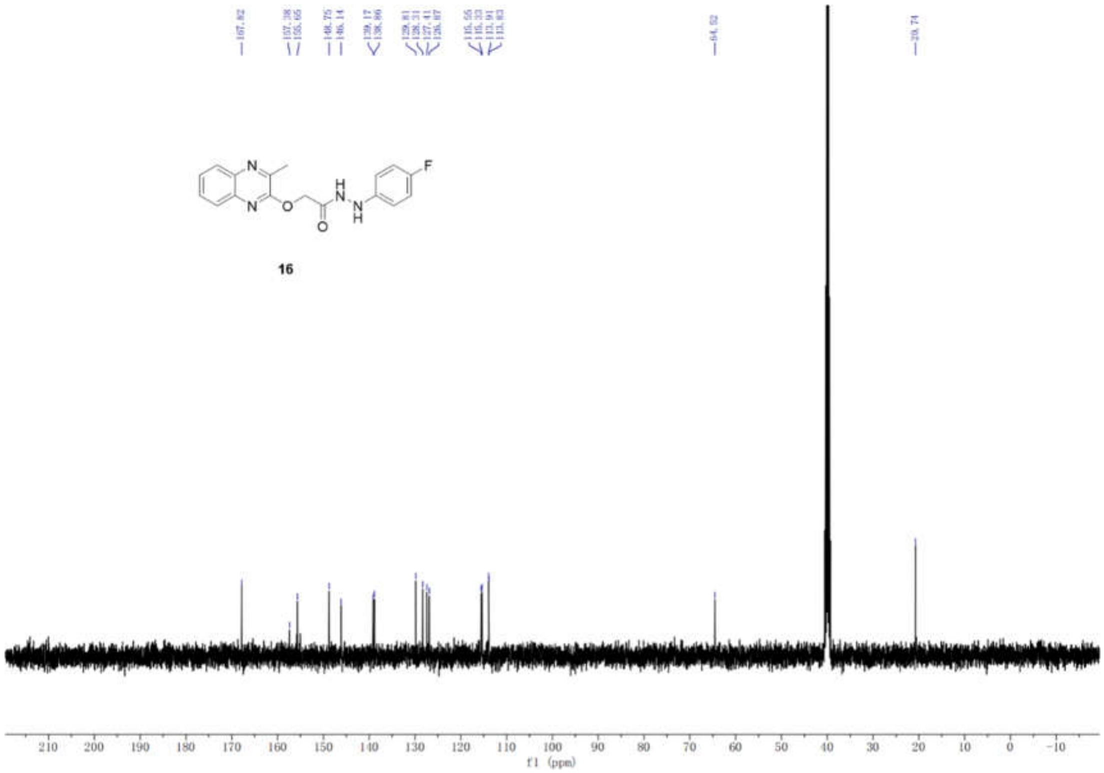

167.82
157.38
155.65
148.75
146.14
139.17
138.86
129.81
128.31
127.41
126.87
115.55
115.33
113.91
113.83
64.52
20.74
16
f1 (ppm)

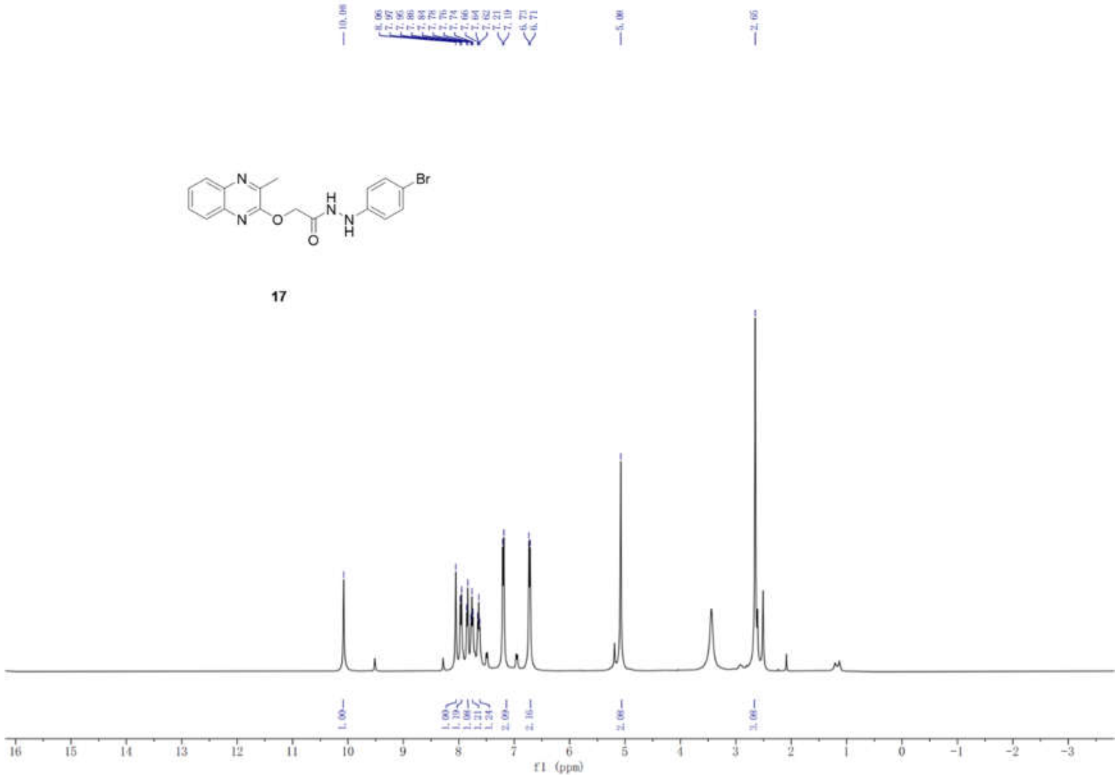
17
f1 (ppm)

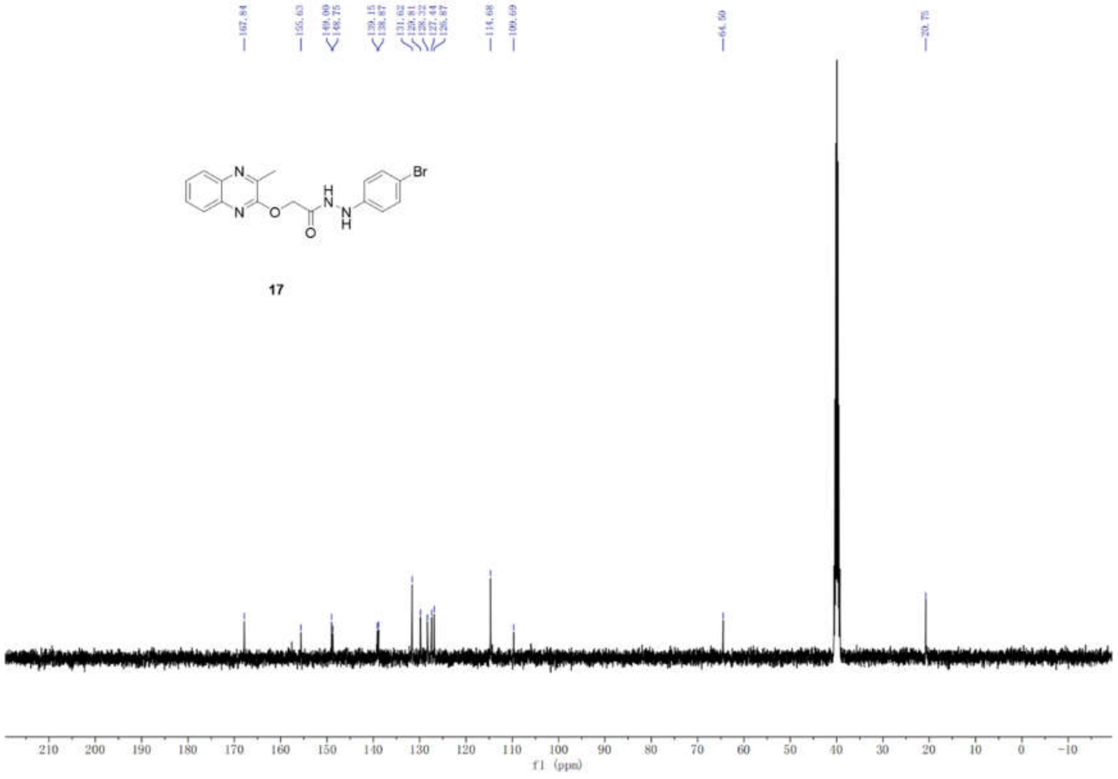
17
f1 (ppm)

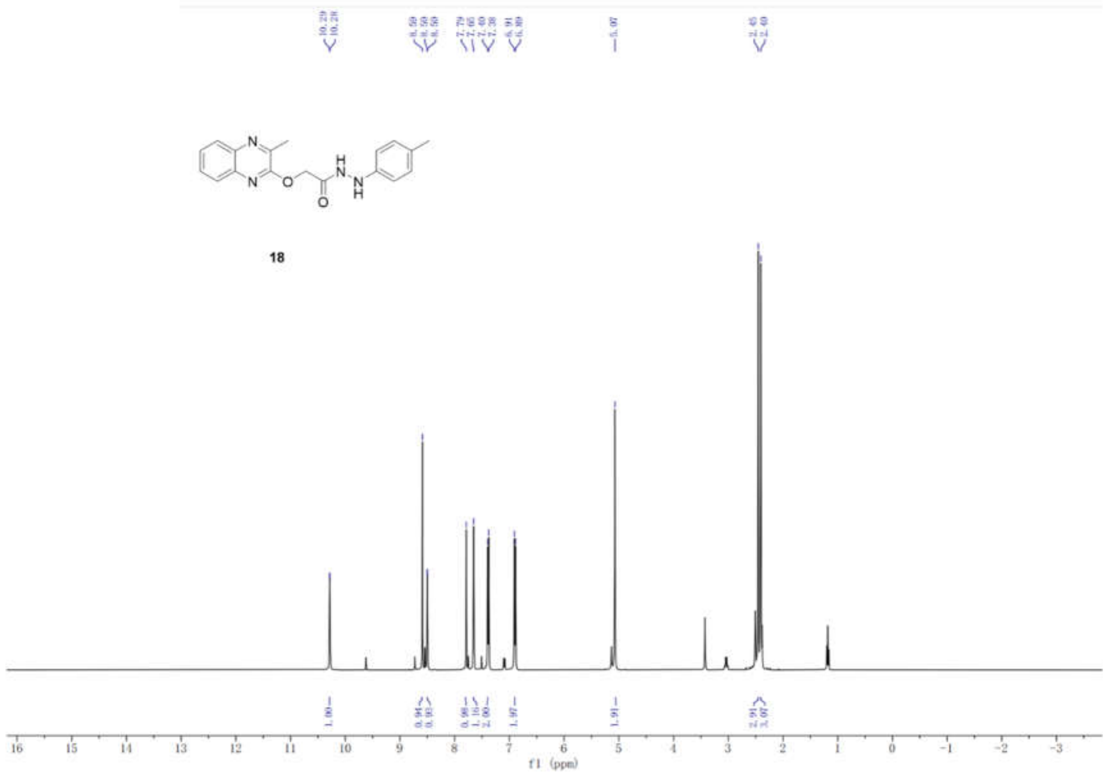
18
f1 (ppm)

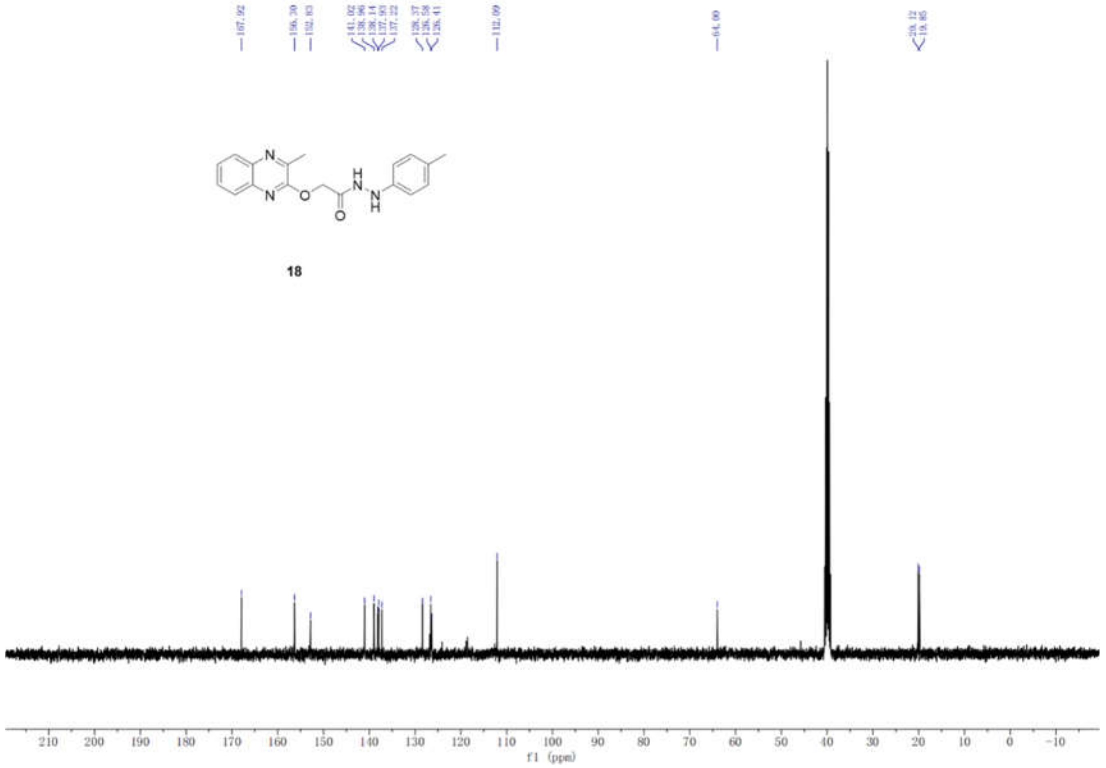
18
f1 (ppm)

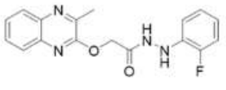

19

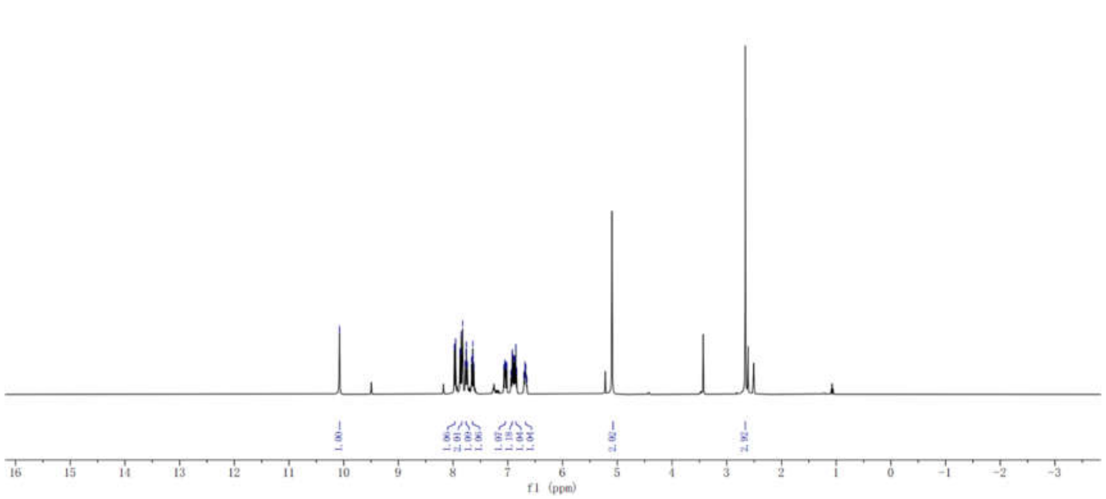

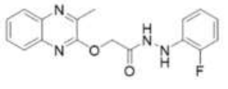

19

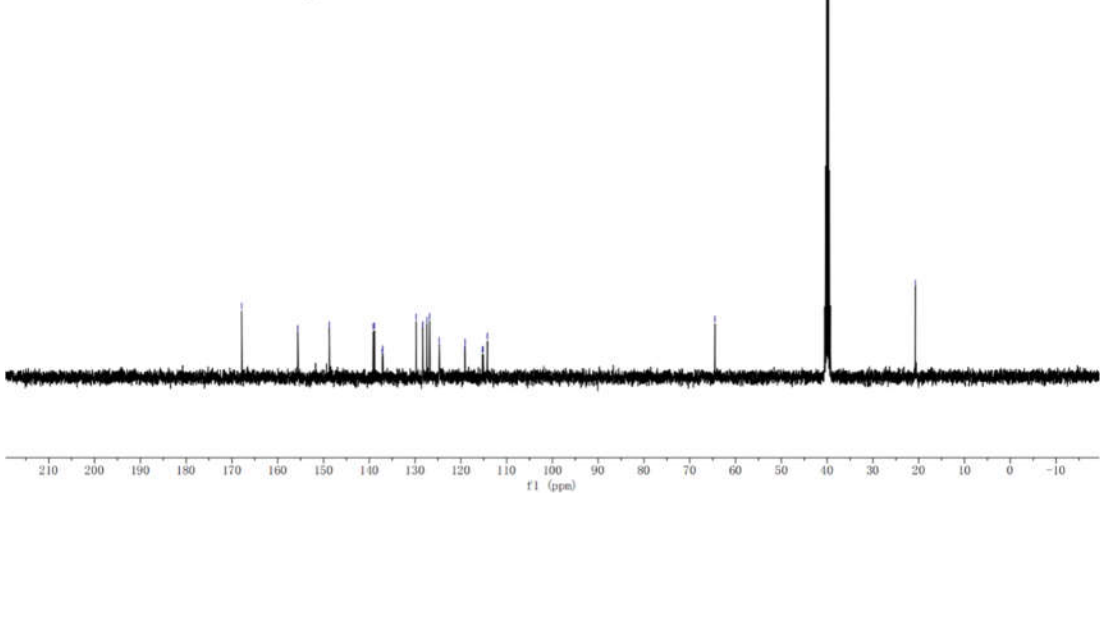

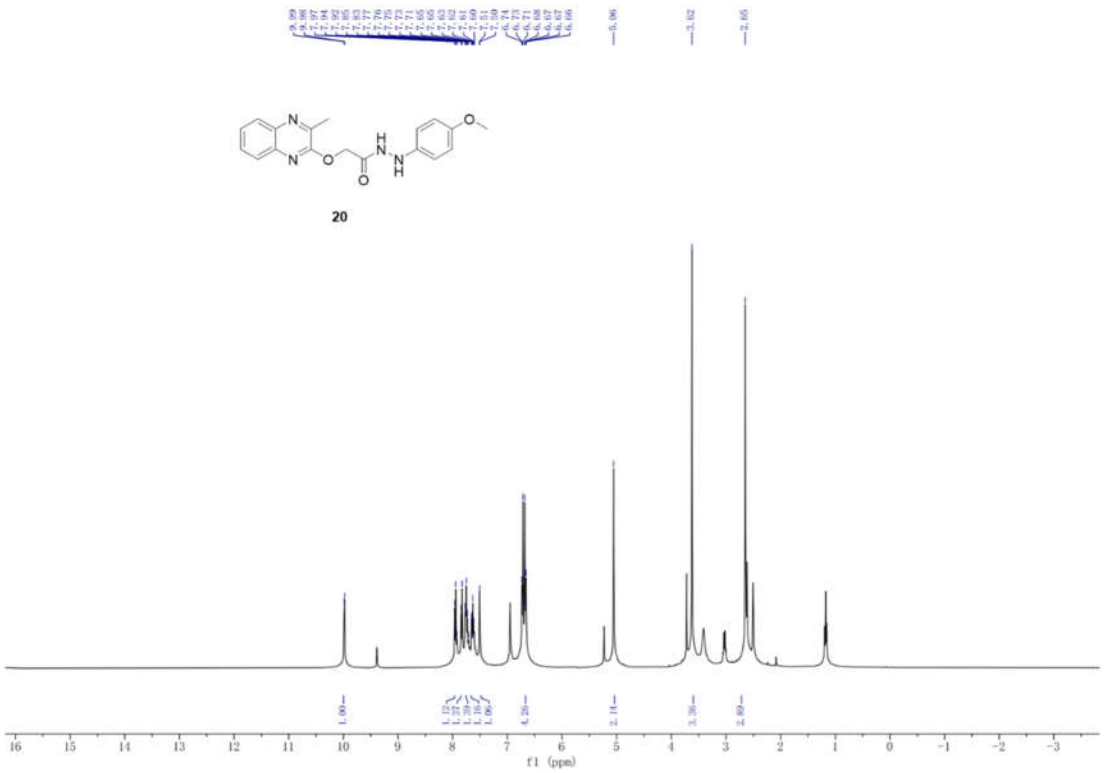

20

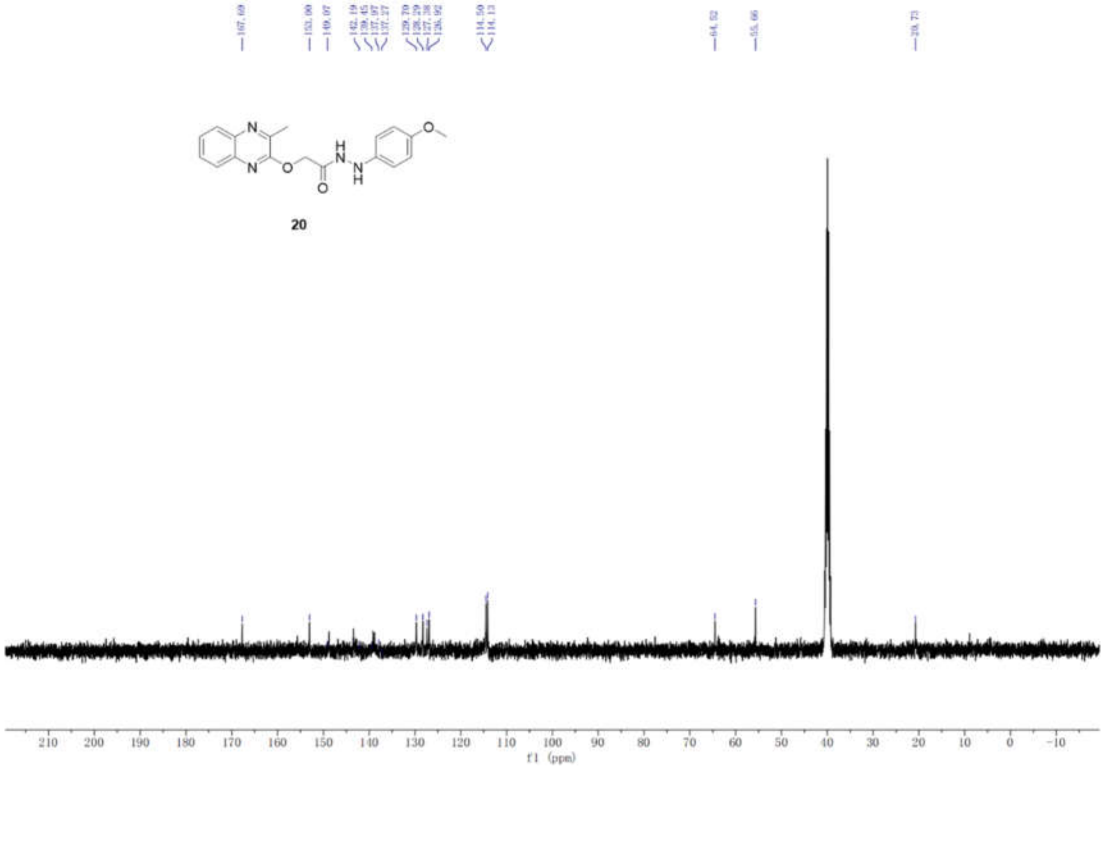

20

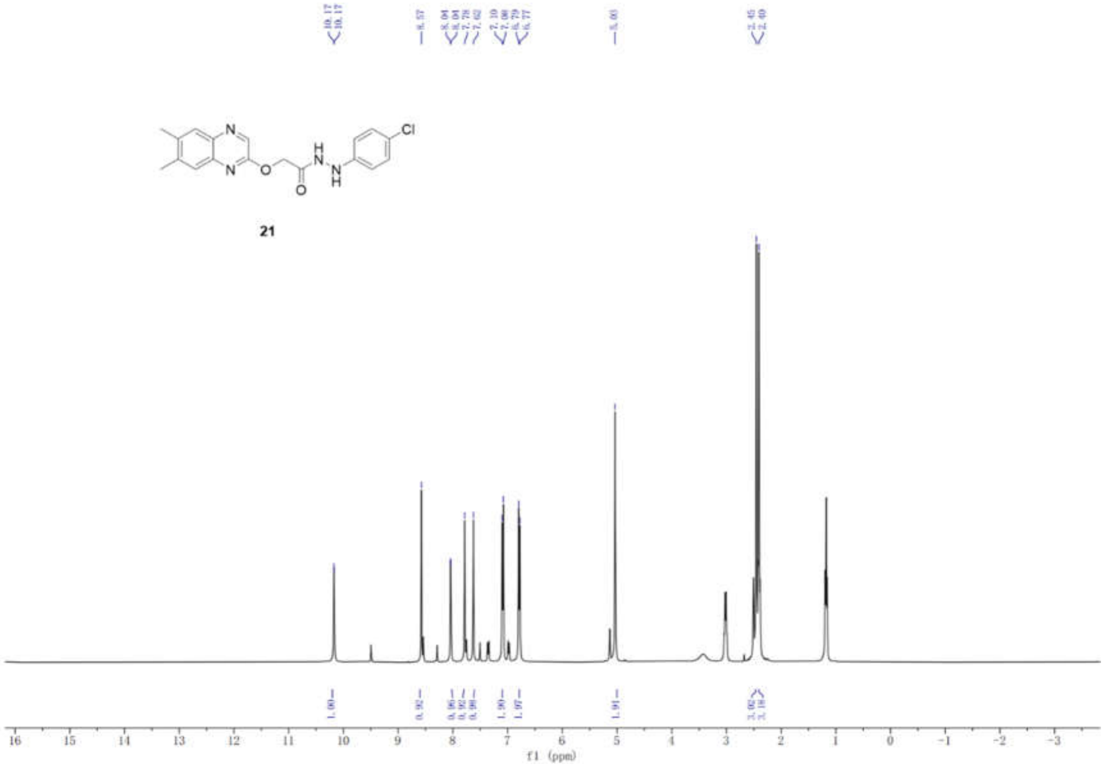
21
f1 (ppm)

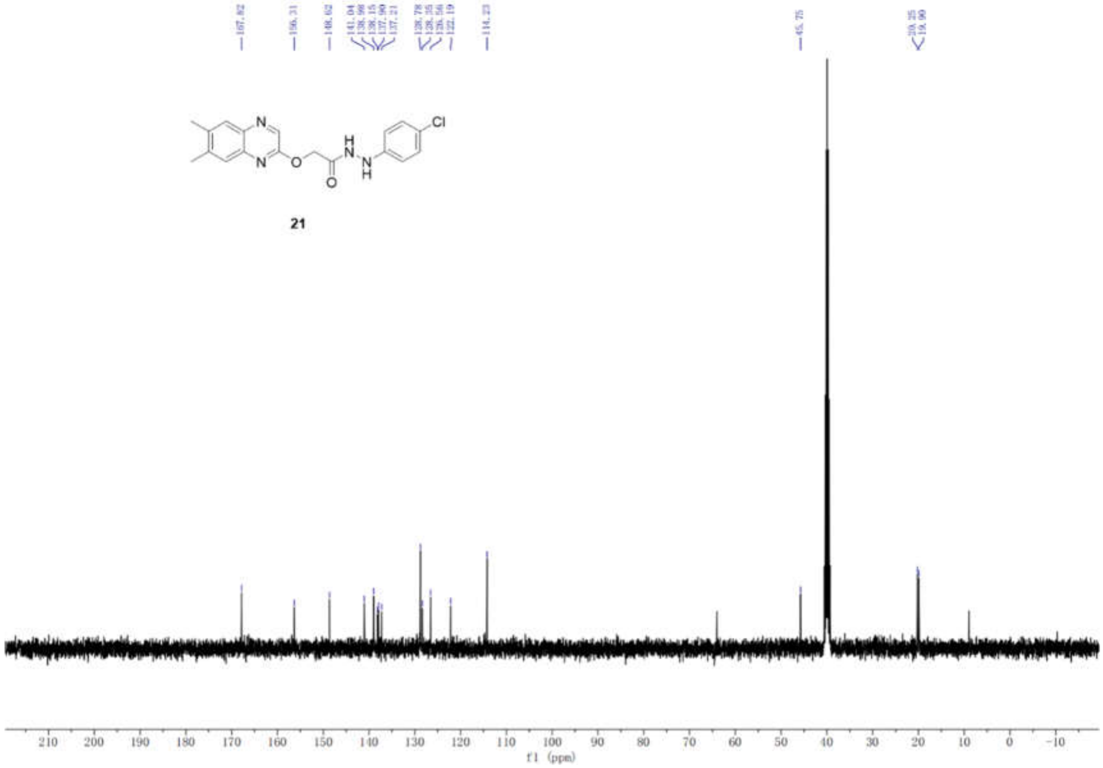
21
f1 (ppm)

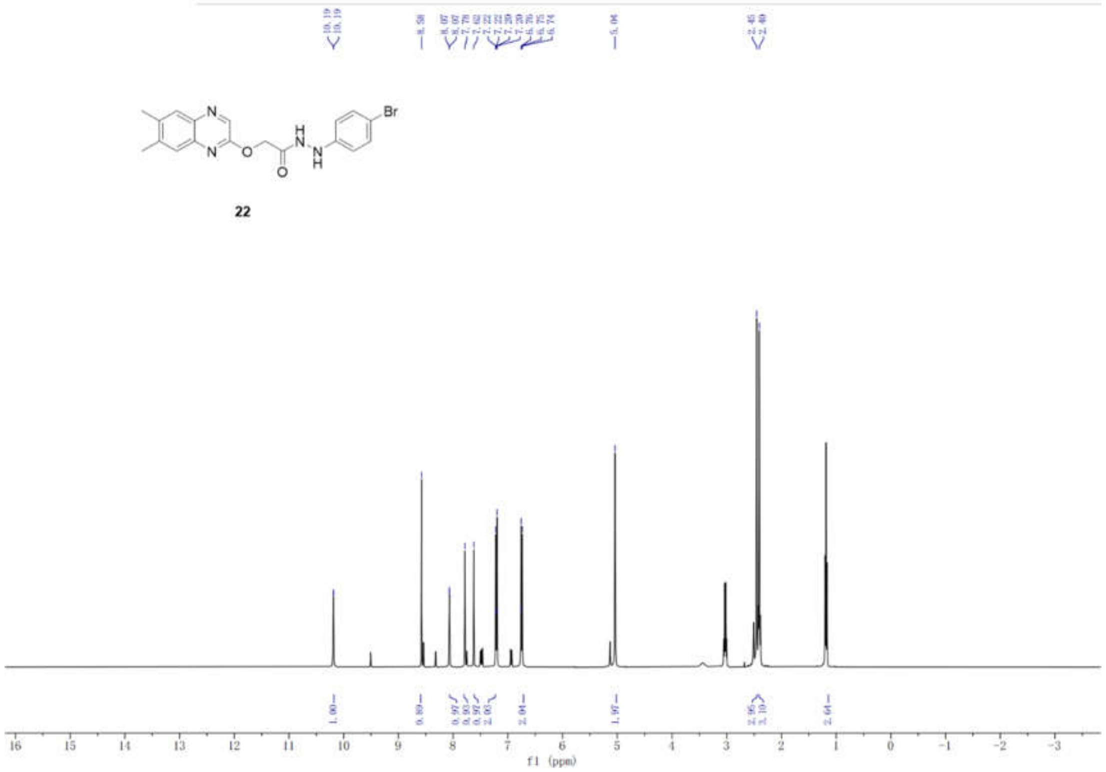

22

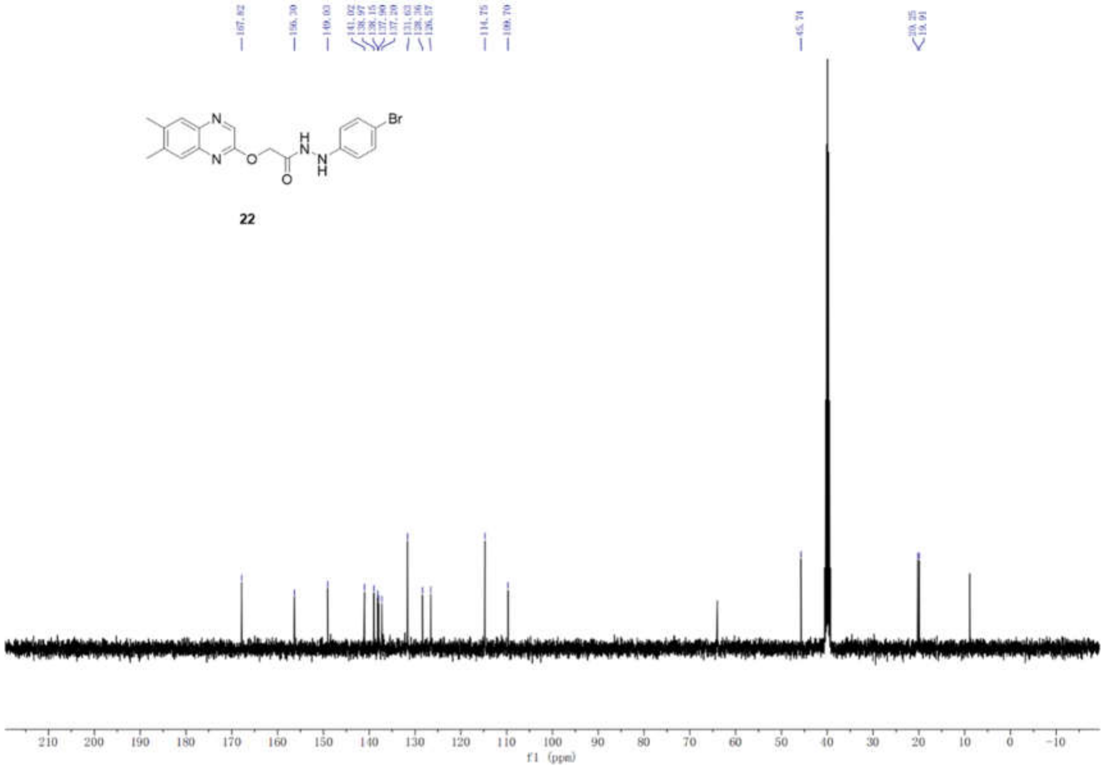

22

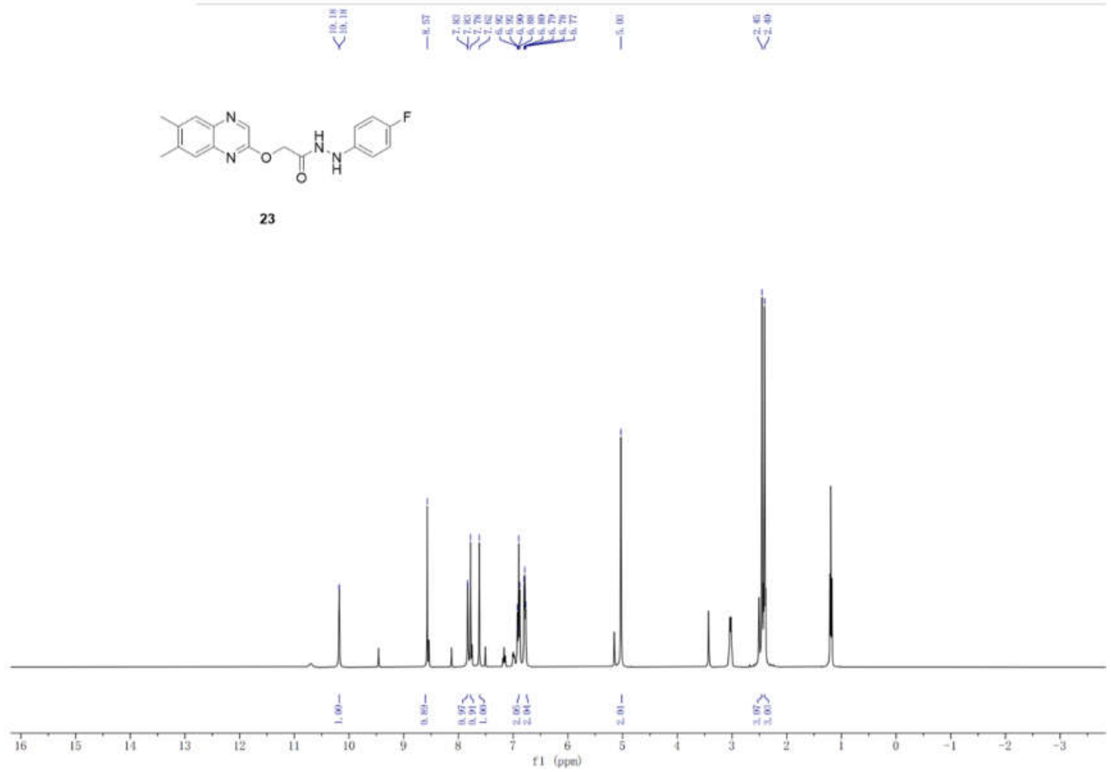

23
f1 (ppm)

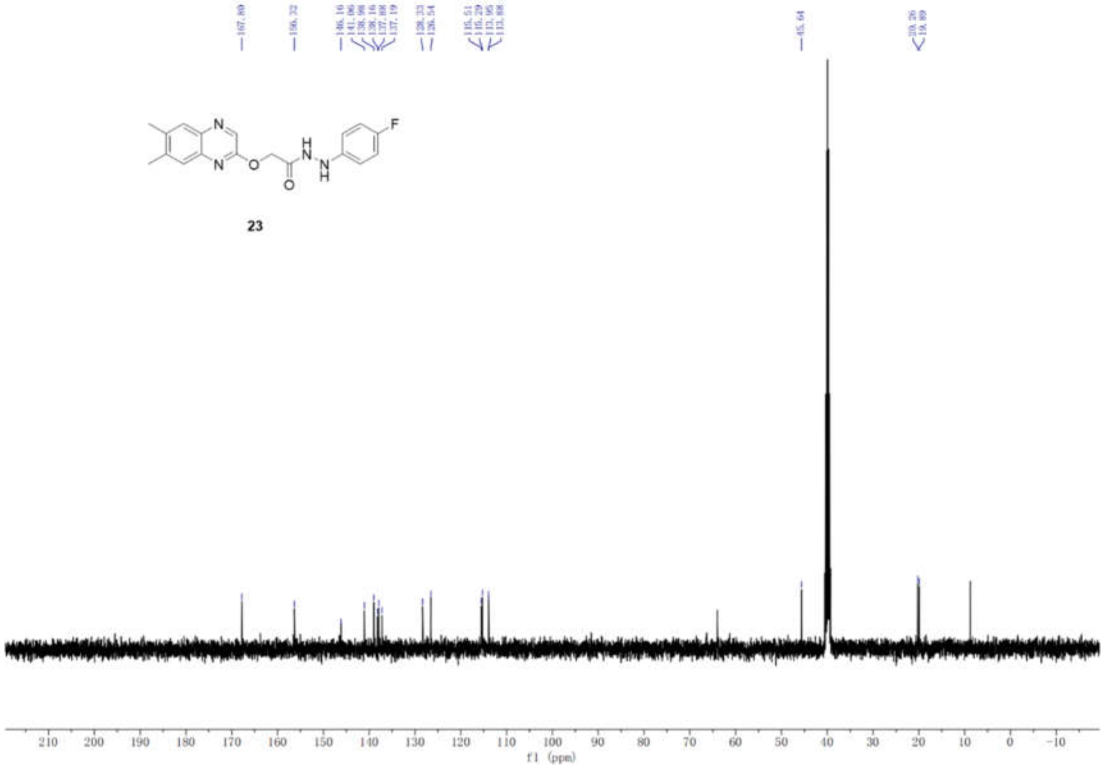

23
f1 (ppm)

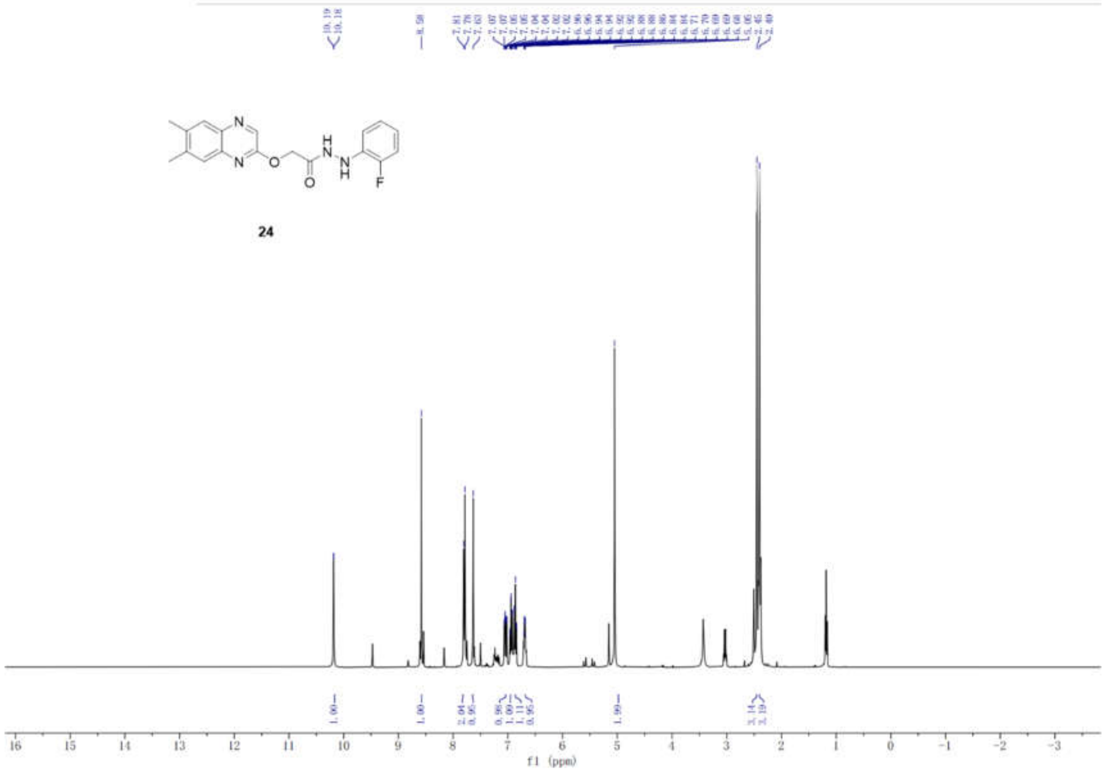

24

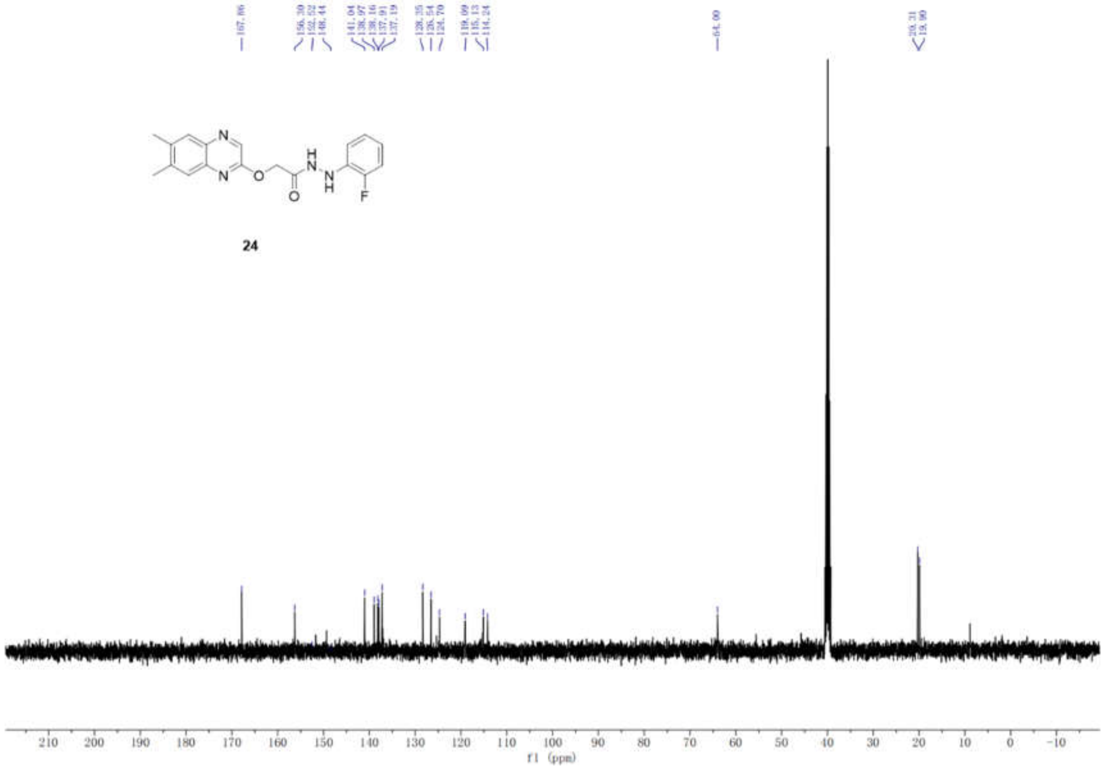

24

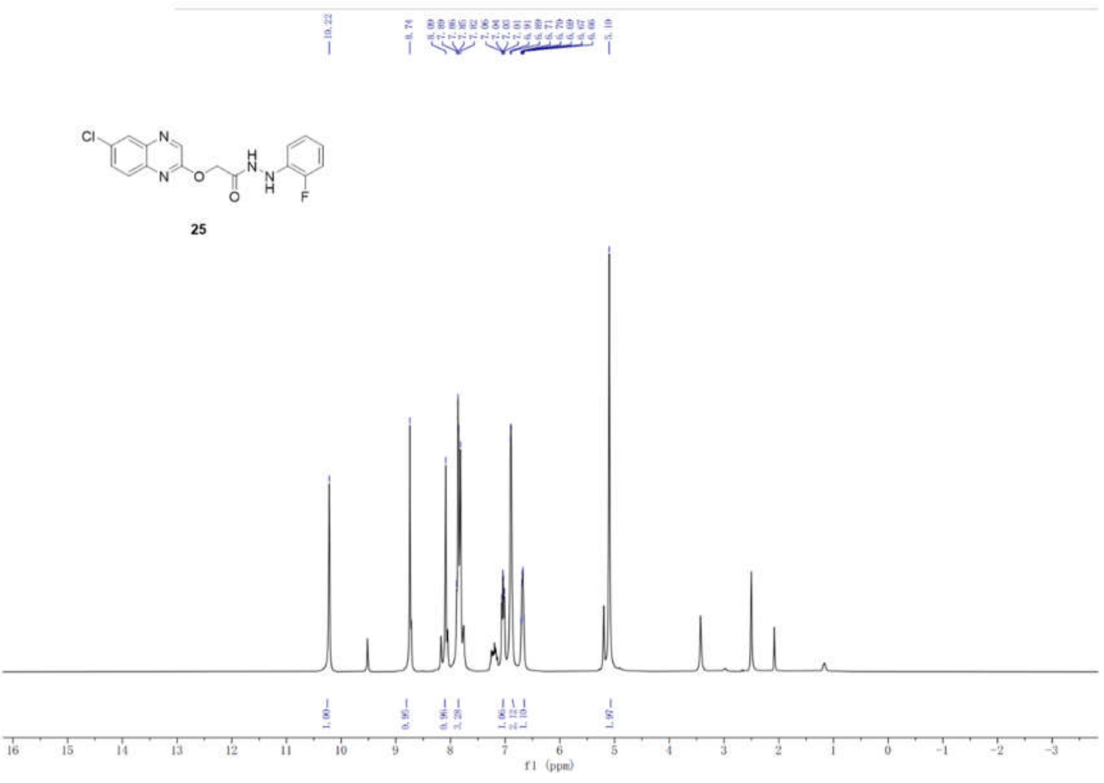

25

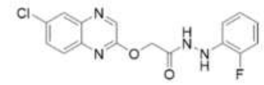

25

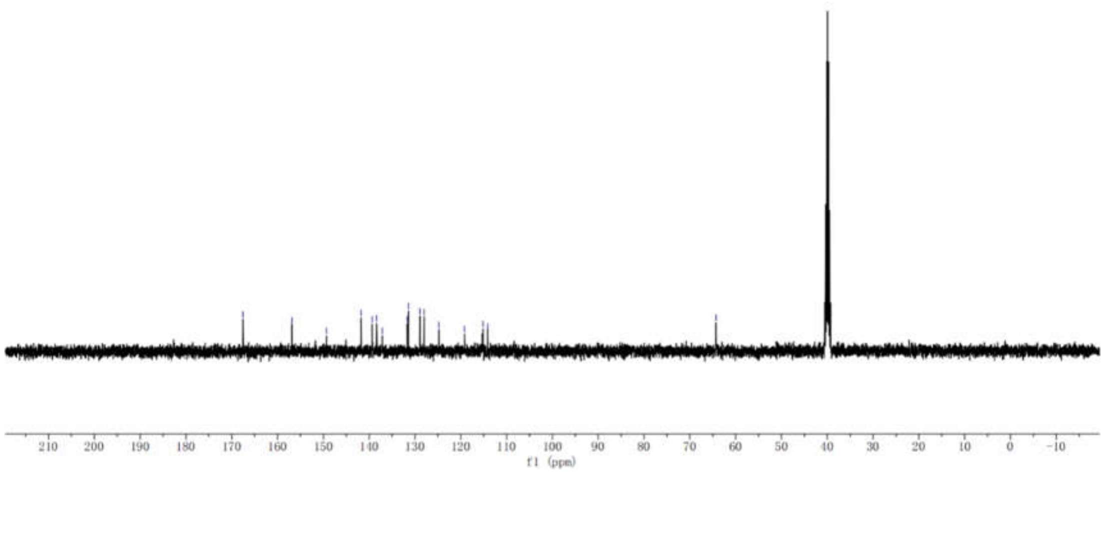

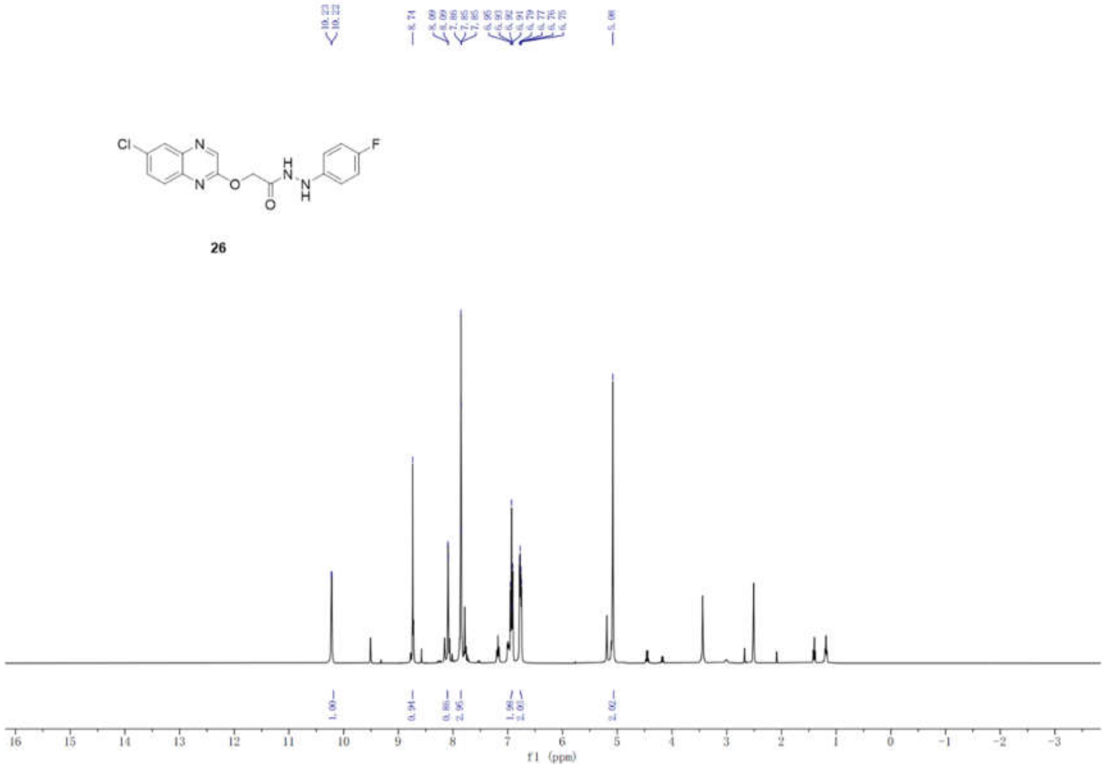

26

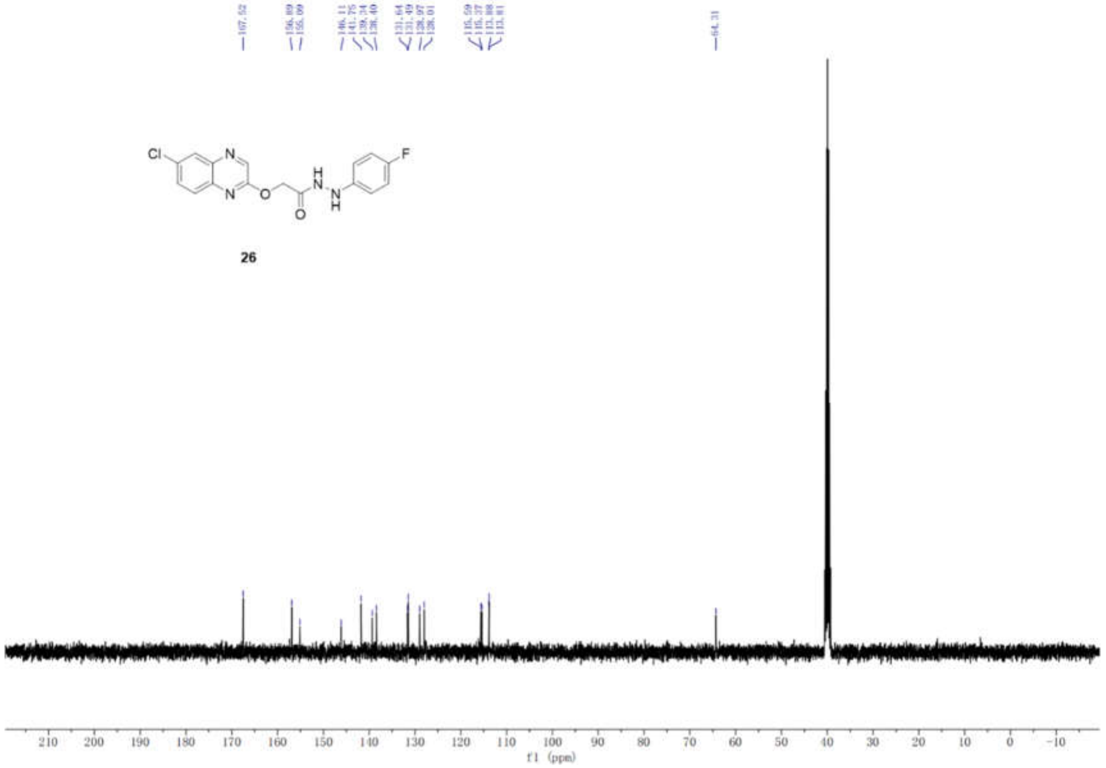

26

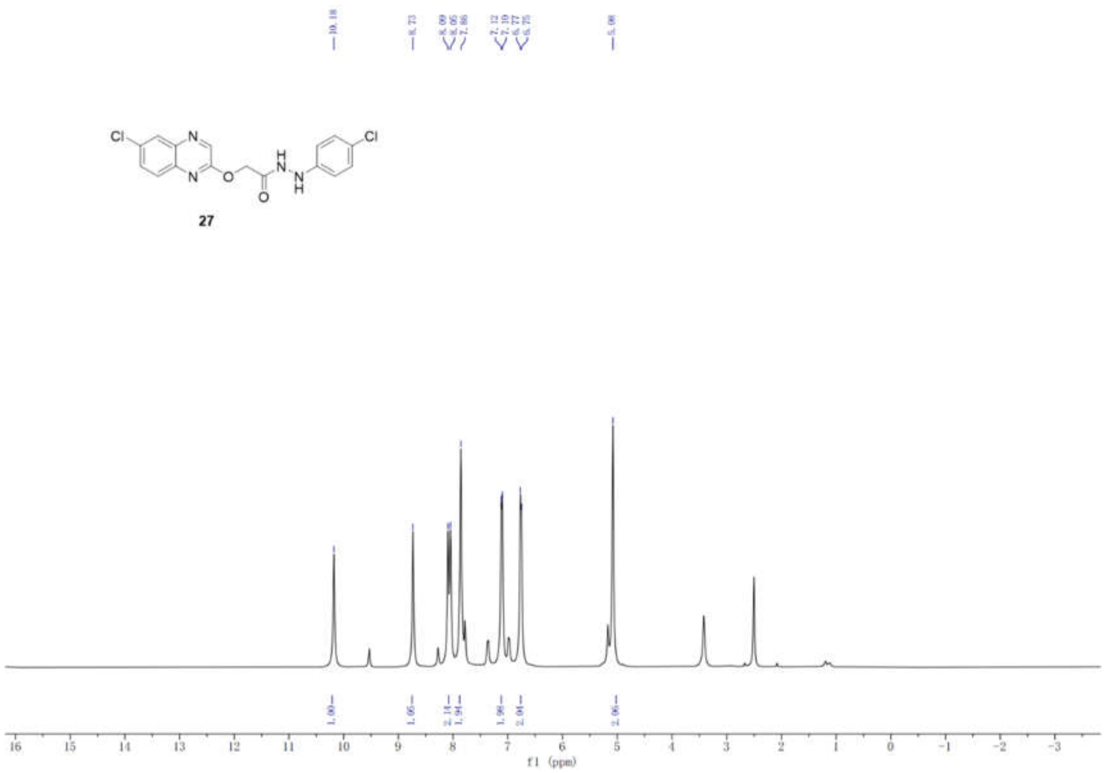
Cl
N
O
H
27
f1 (ppm)

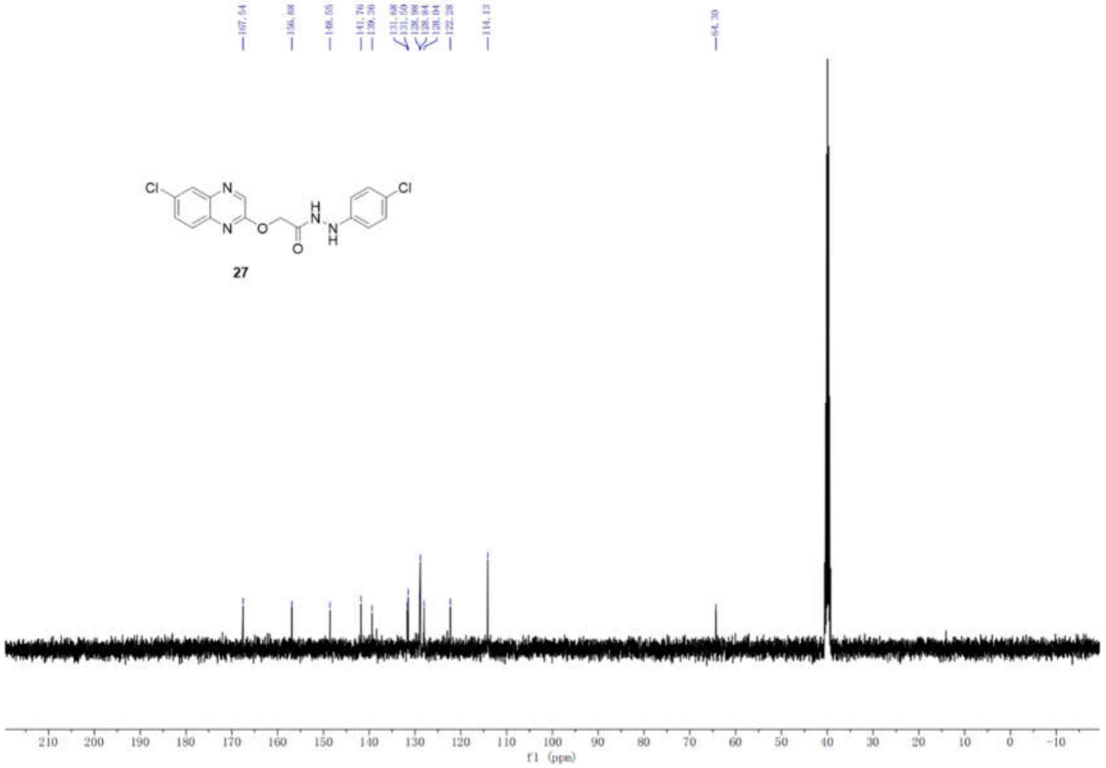
Cl
N
O
H
27
f1 (ppm)

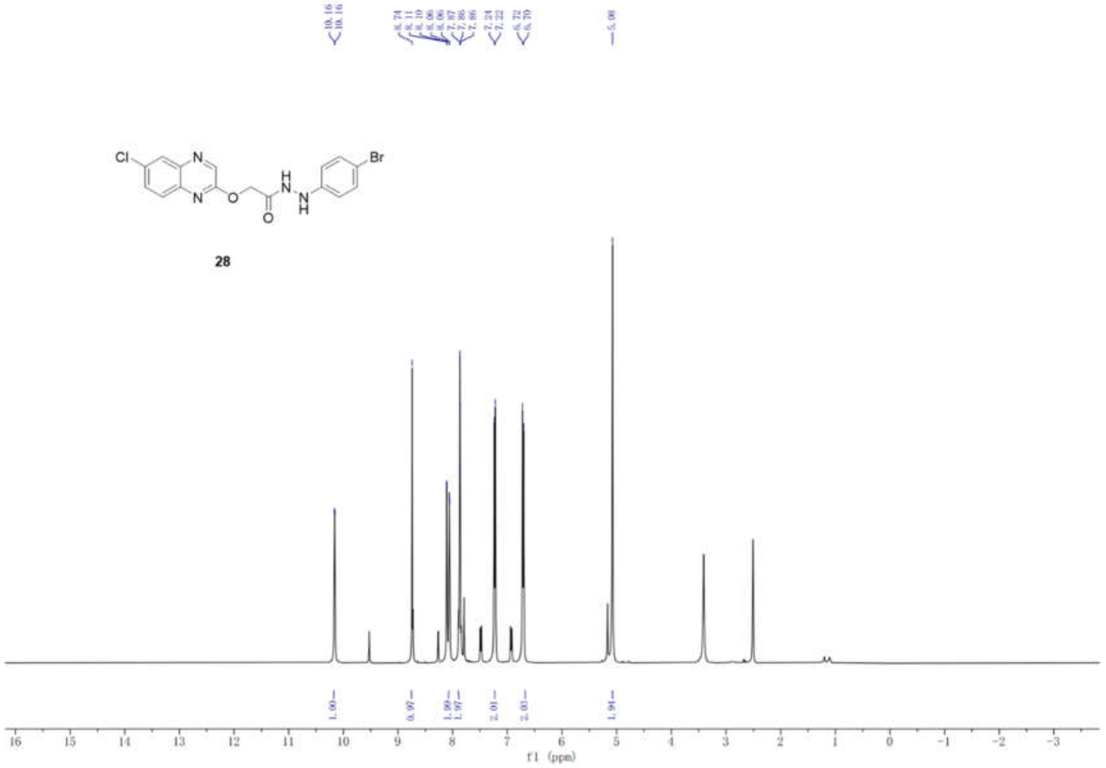
10.16
10.16
8.74
8.11
8.10
8.06
8.06
7.87
7.86
7.86
7.24
7.22
6.72
6.70
5.08
Cl
N
N
O
O
H
N
N
H
Br
28
1.00
0.97
1.99
1.97
2.01
2.03
1.94
f1 (ppm)

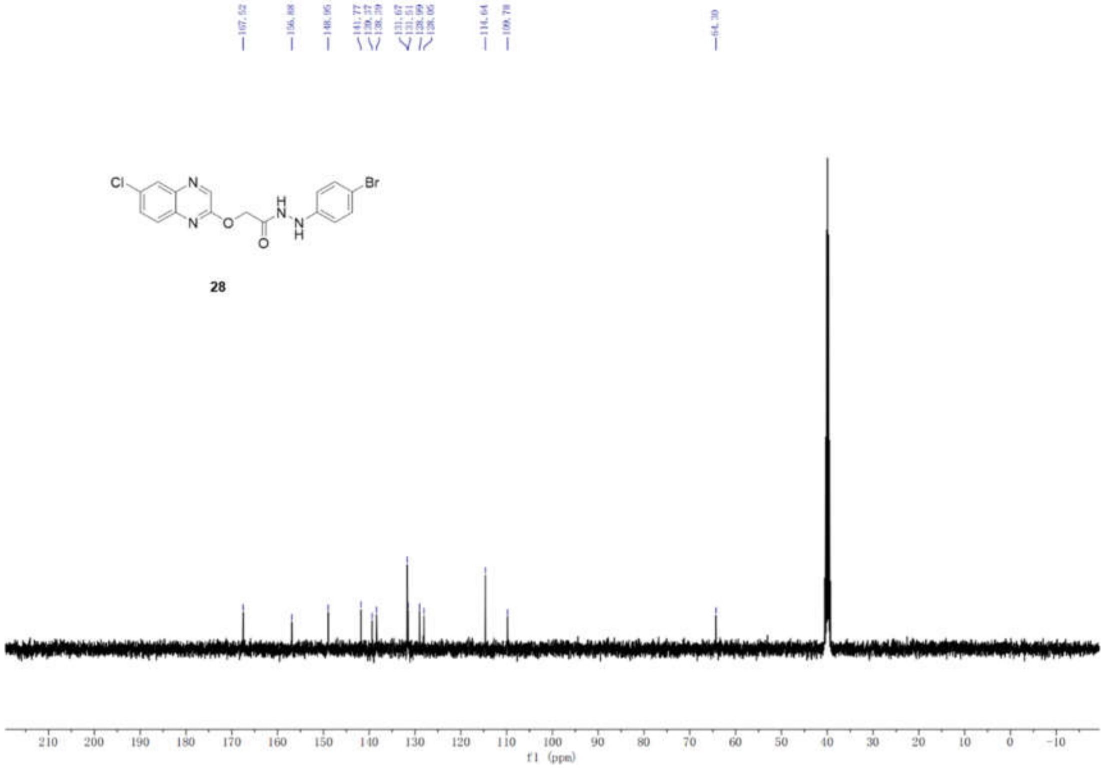
167.52
156.88
148.95
141.77
139.37
138.39
131.67
131.51
128.99
128.05
114.64
109.78
64.30
Cl
N
N
O
O
H
N
N
H
Br
28
f1 (ppm)

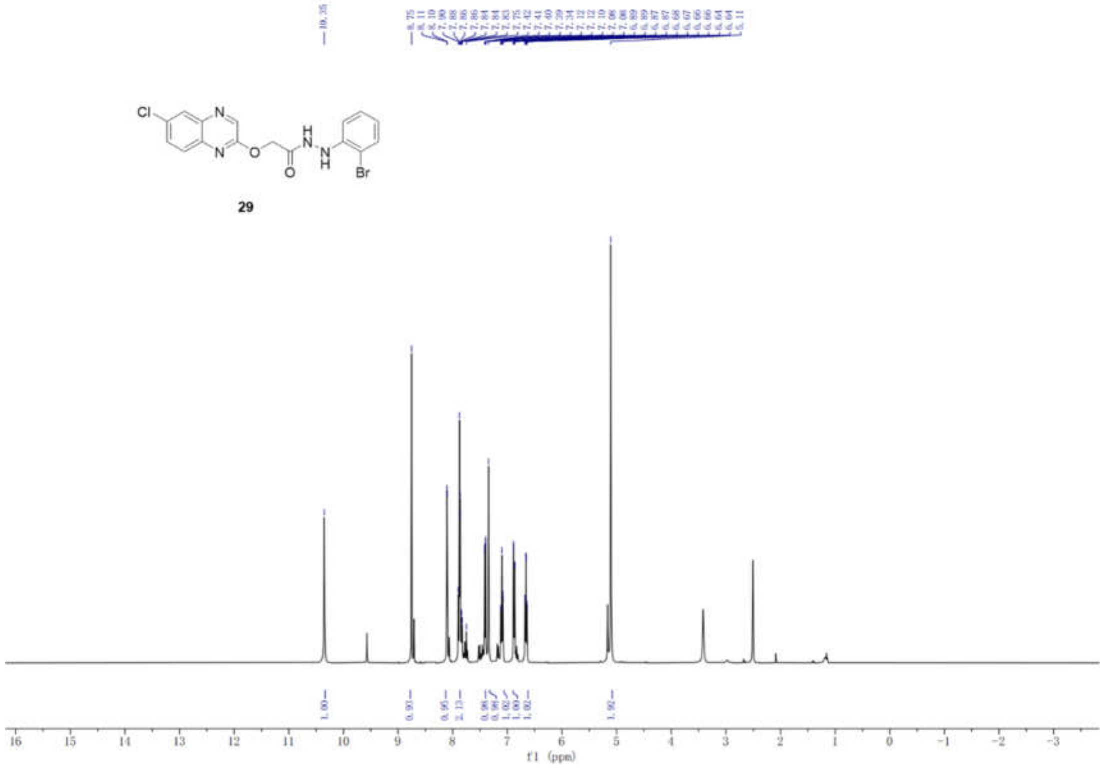
Cl
Br
29
f1 (ppm)

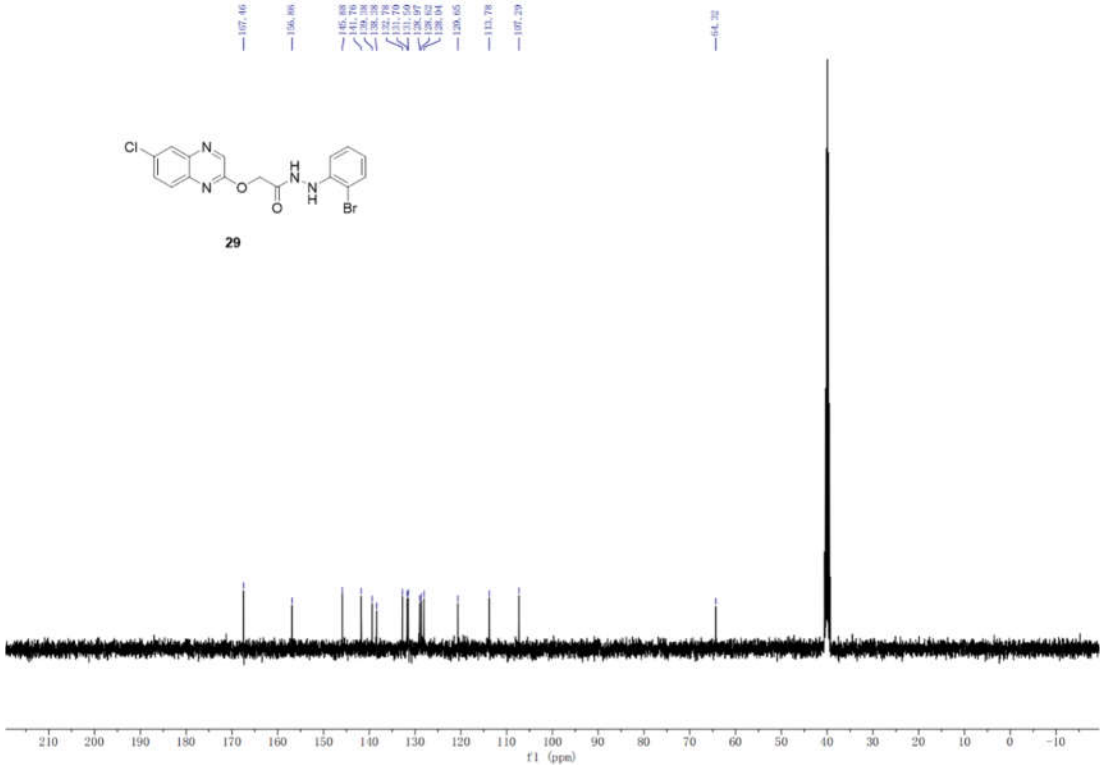
Cl
Br
29
f1 (ppm)

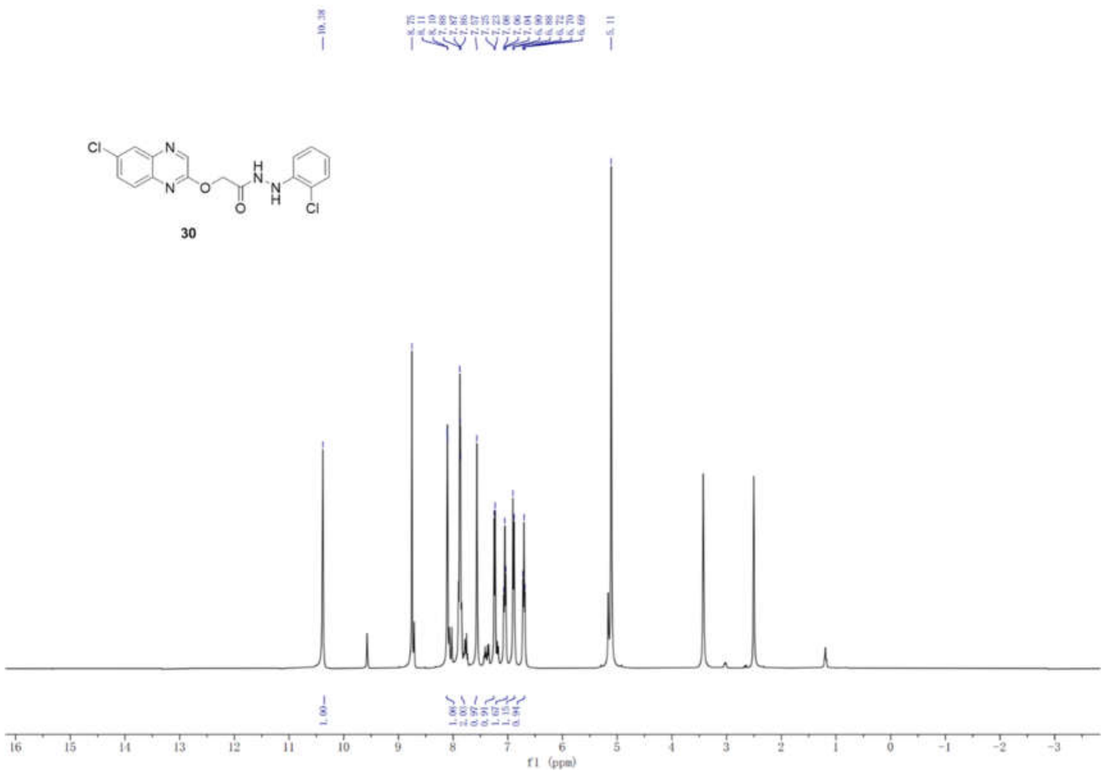
10.38
8.75
8.11
8.10
7.88
7.87
7.86
7.57
7.25
7.23
7.08
7.06
7.04
6.90
6.88
6.72
6.70
6.69
5.11
Cl
N
O
H
Cl
30
1.00
1.06
2.05
0.97
0.91
1.67
1.15
0.94
f1 (ppm)

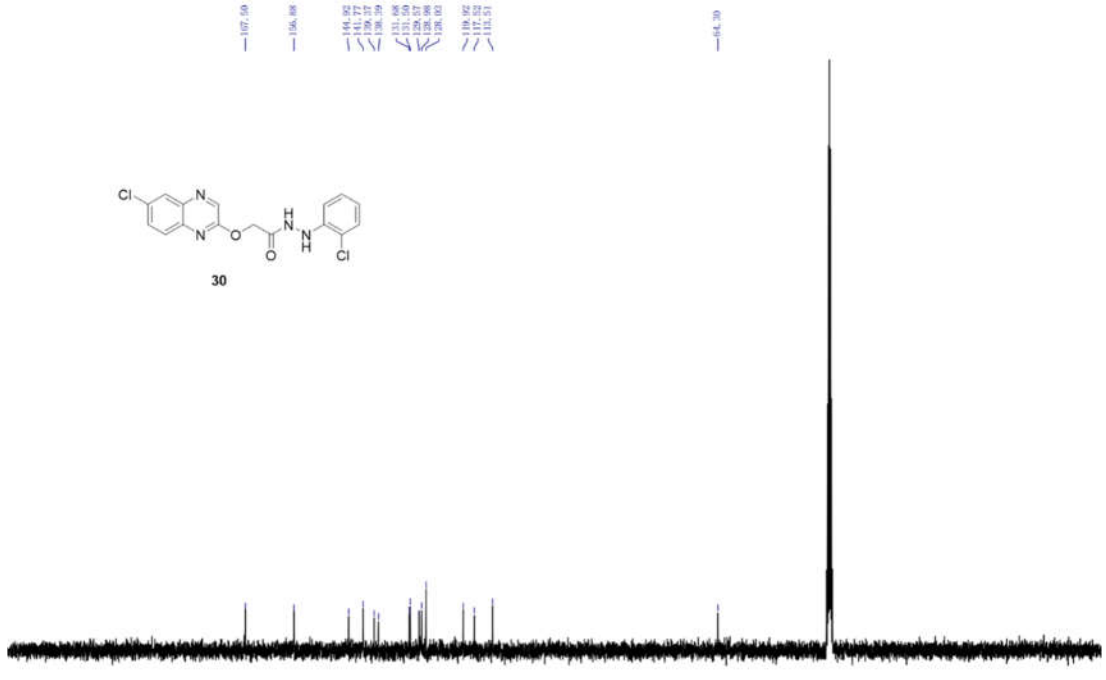
167.59
156.88
144.92
141.77
139.37
138.39
131.68
131.50
129.57
128.98
128.03
119.92
117.52
113.51
64.30
Cl
N
O
H
Cl
30
f1 (ppm)

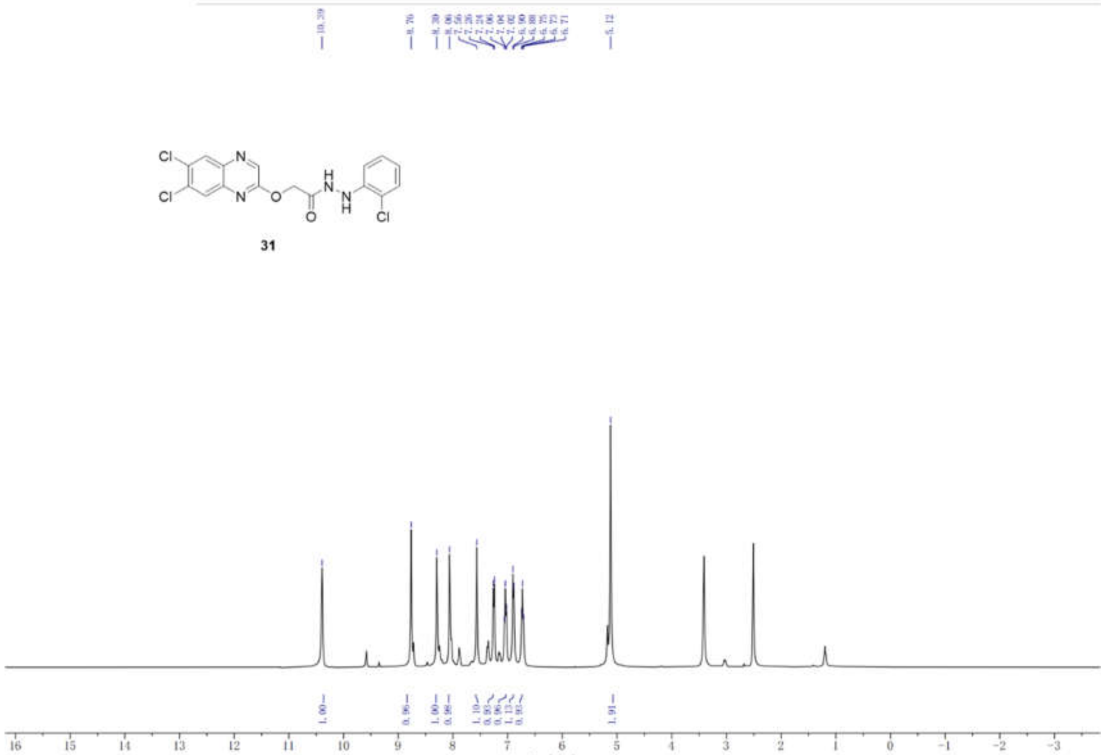
31
f1 (ppm)

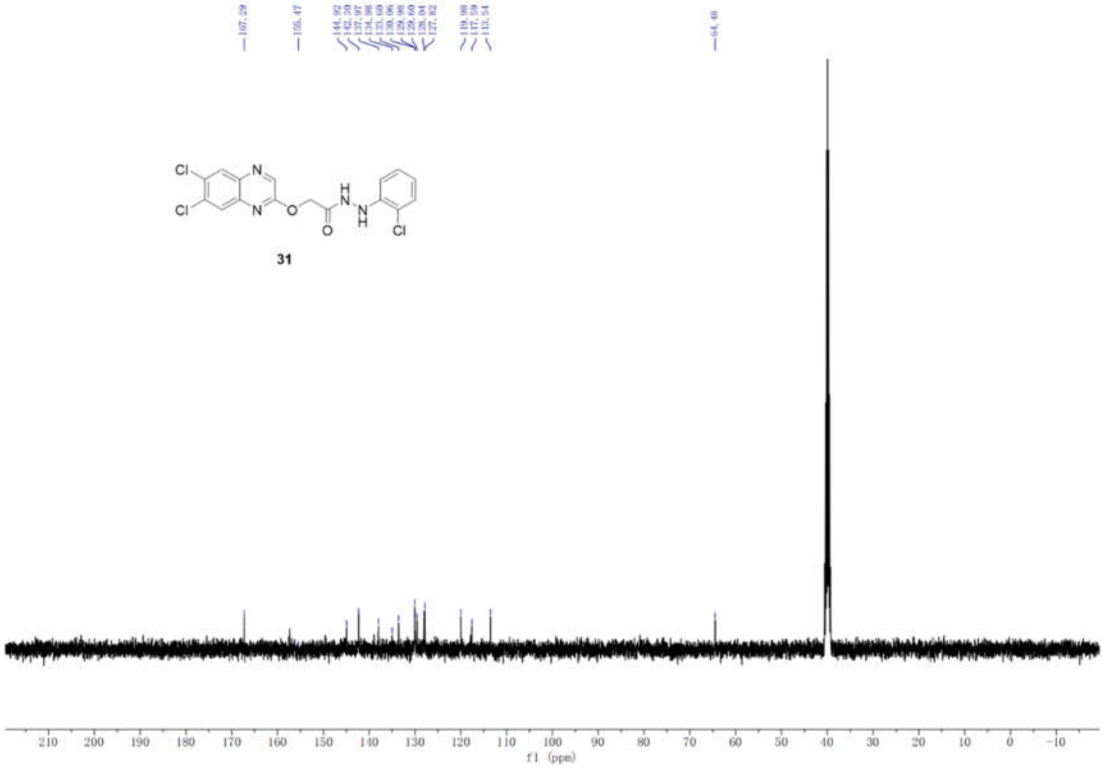
31
f1 (ppm)

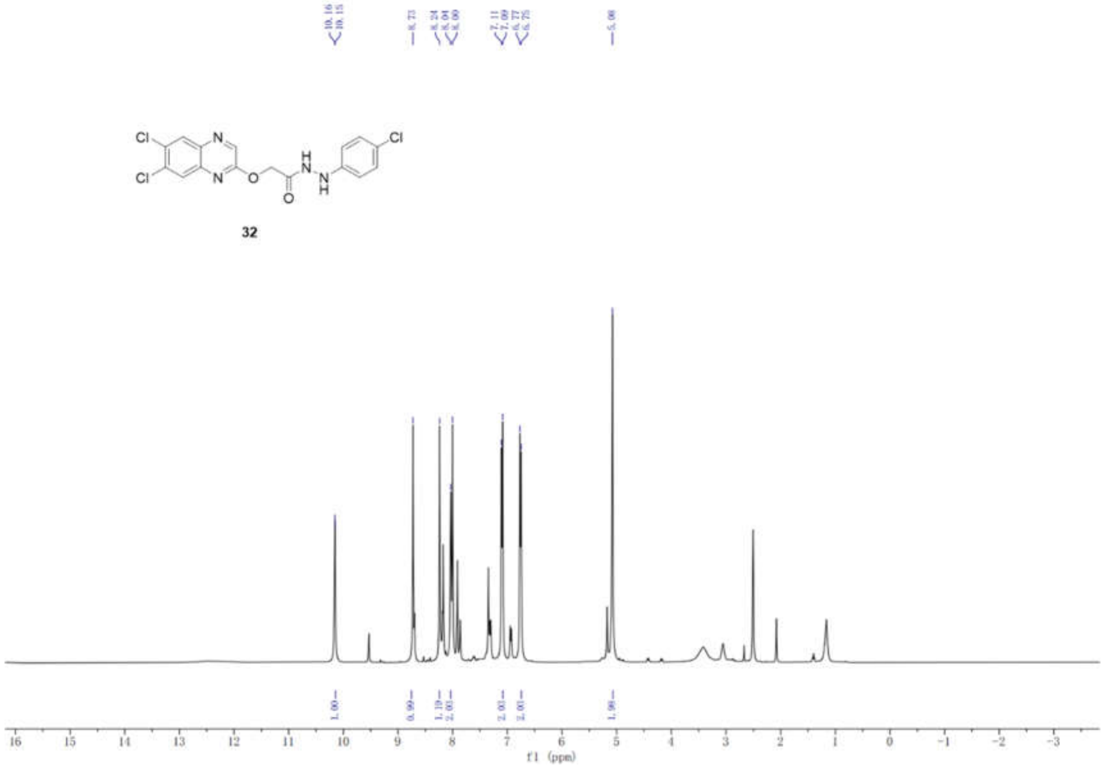
10.16
10.15
8.73
8.24
8.04
8.00
7.11
7.09
6.77
6.75
5.08
Cl
N
O
H
Cl
32
1.00
0.99
1.19
2.00
2.00
2.00
1.98
f1 (ppm)

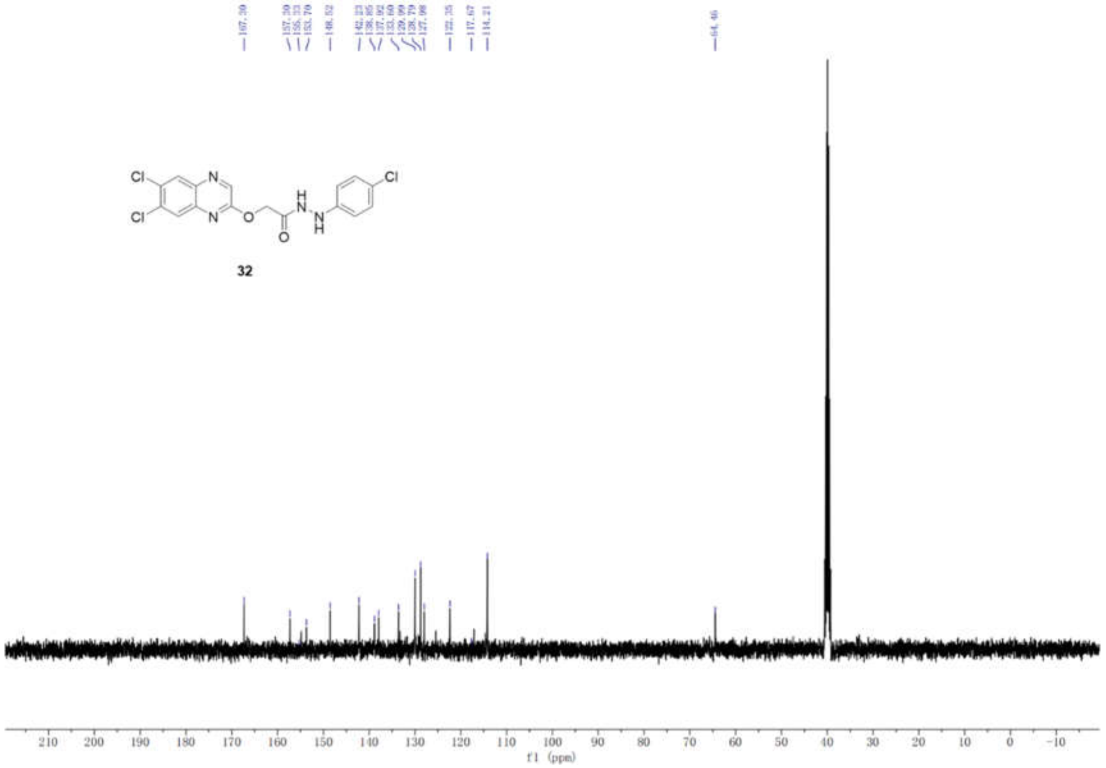
167.39
157.59
155.33
153.70
148.52
142.23
138.85
137.92
133.60
129.99
128.79
127.98
122.35
117.67
114.21
64.46
Cl
N
O
H
32
f1 (ppm)

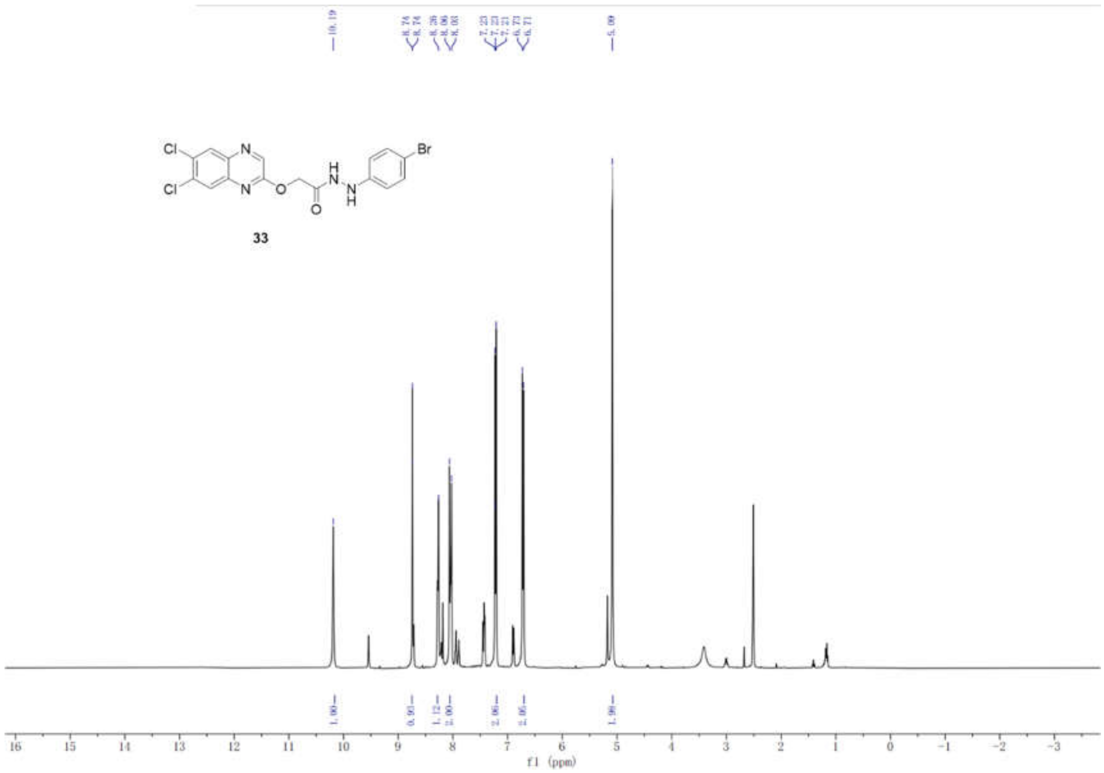
10.19
8.74
8.74
8.26
8.06
8.03
7.23
7.23
7.21
6.73
6.71
5.09
Cl
Cl
N
N
O
H
N
N
H
O
Br
33
1.00
0.95
1.12
2.00
2.06
2.05
1.98
f1 (ppm)

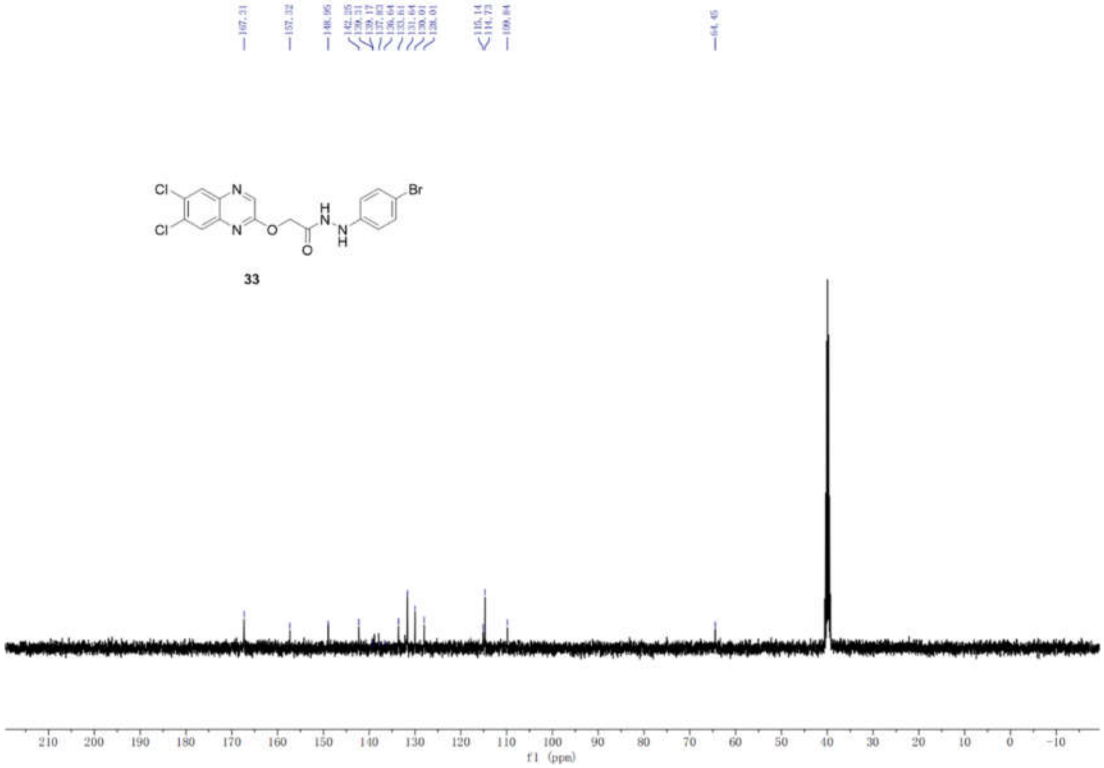
167.31
157.32
148.95
142.25
139.31
139.17
137.83
136.64
133.61
131.64
130.01
128.01
115.14
114.73
109.84
64.45
Cl
Cl
N
N
O
H
N
N
H
O
Br
33
f1 (ppm)

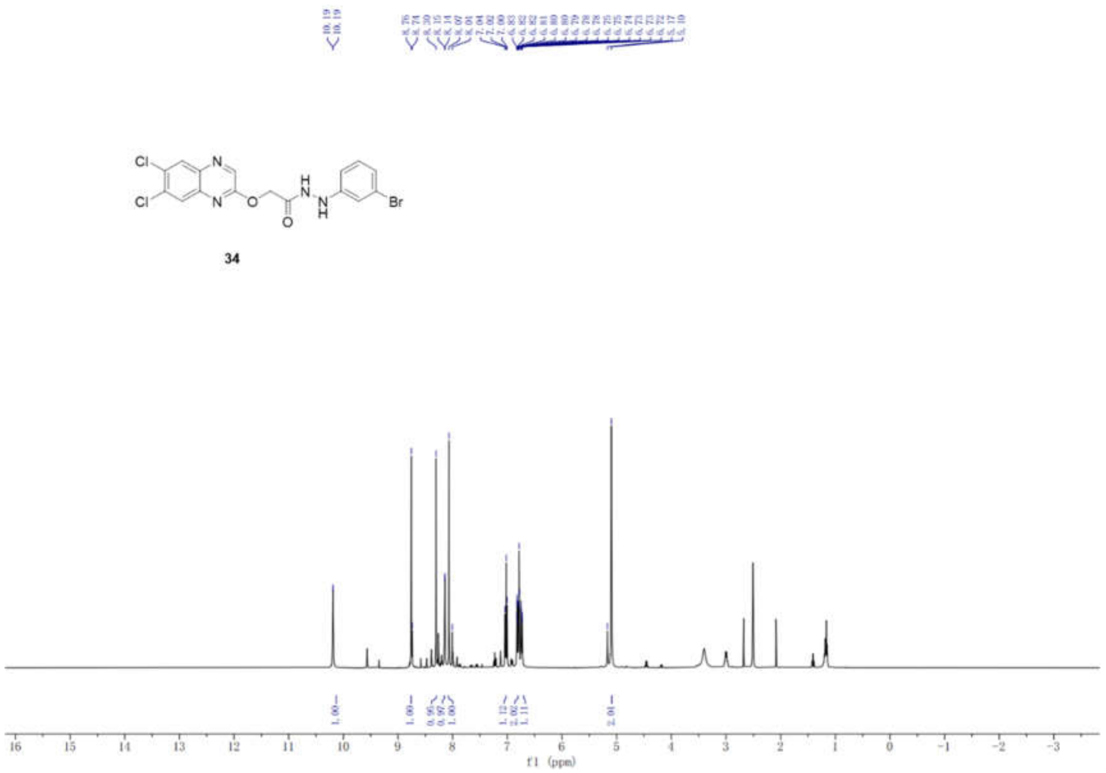
34
f1 (ppm)

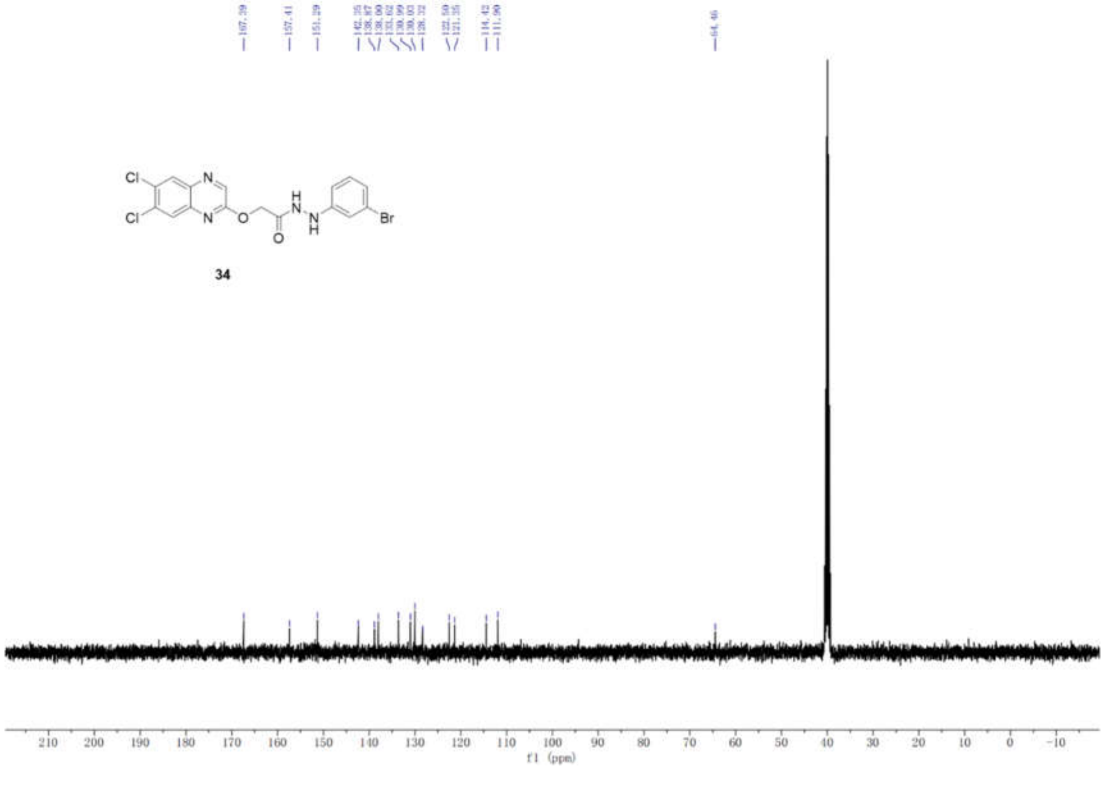
34
f1 (ppm)

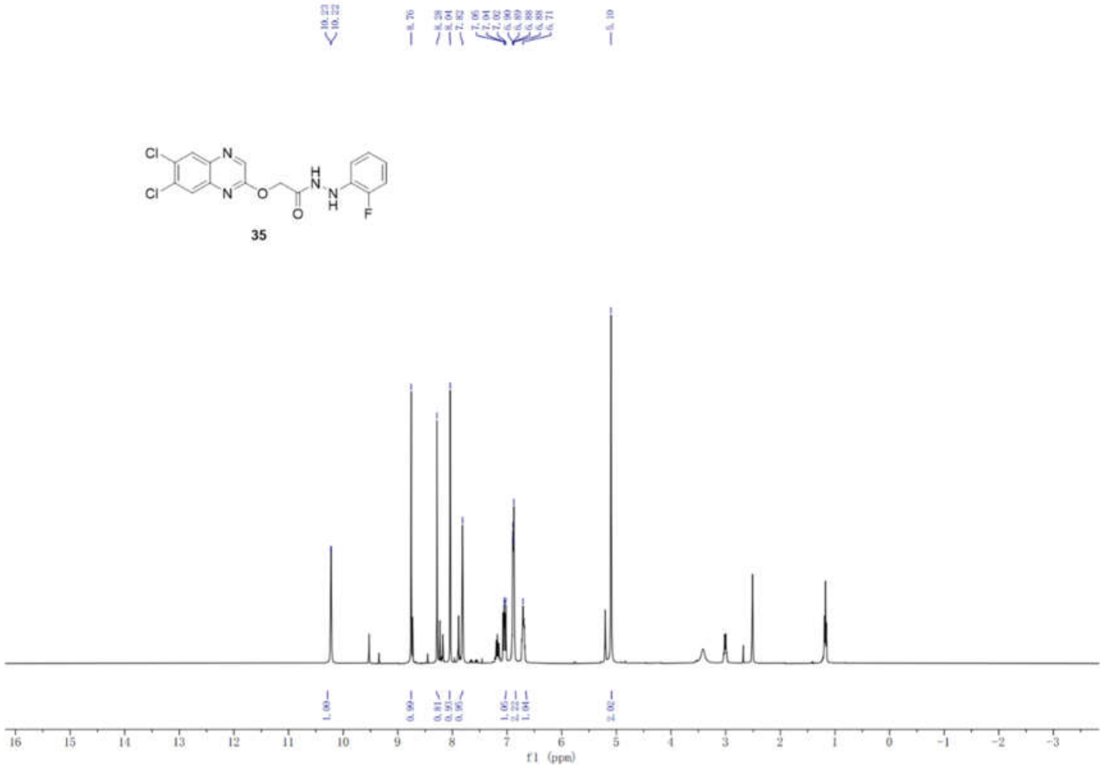
35
f1 (ppm)

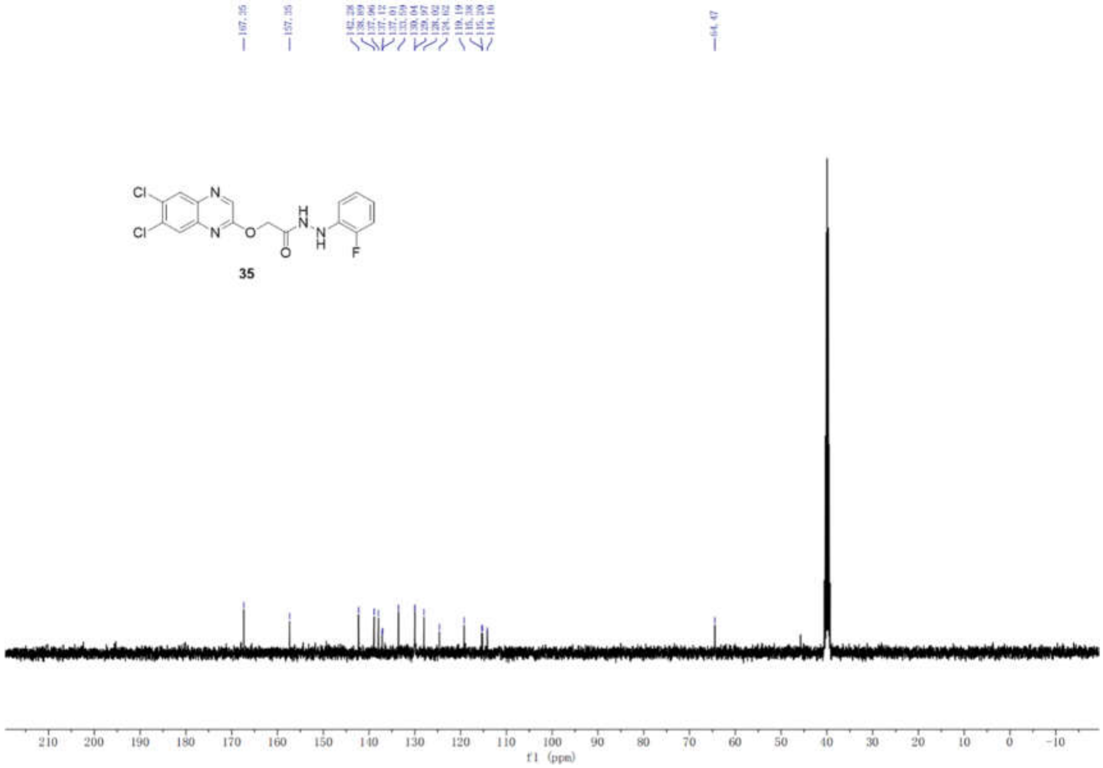
35
f1 (ppm)

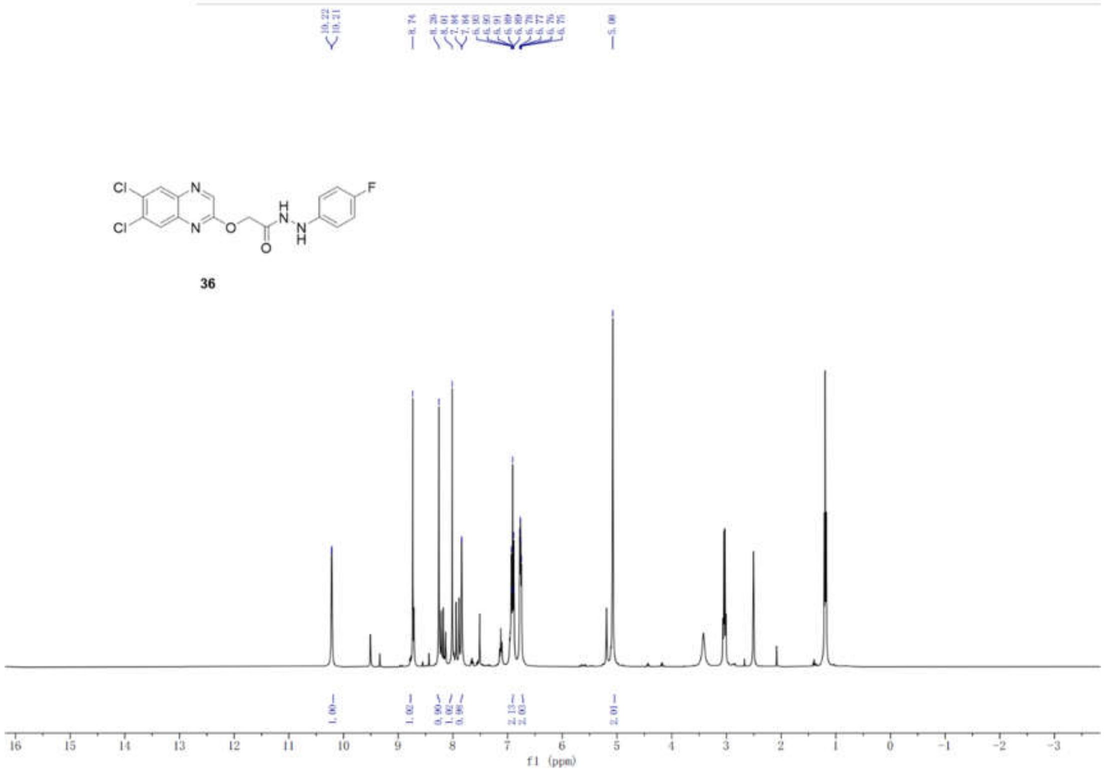

36
f1 (ppm)

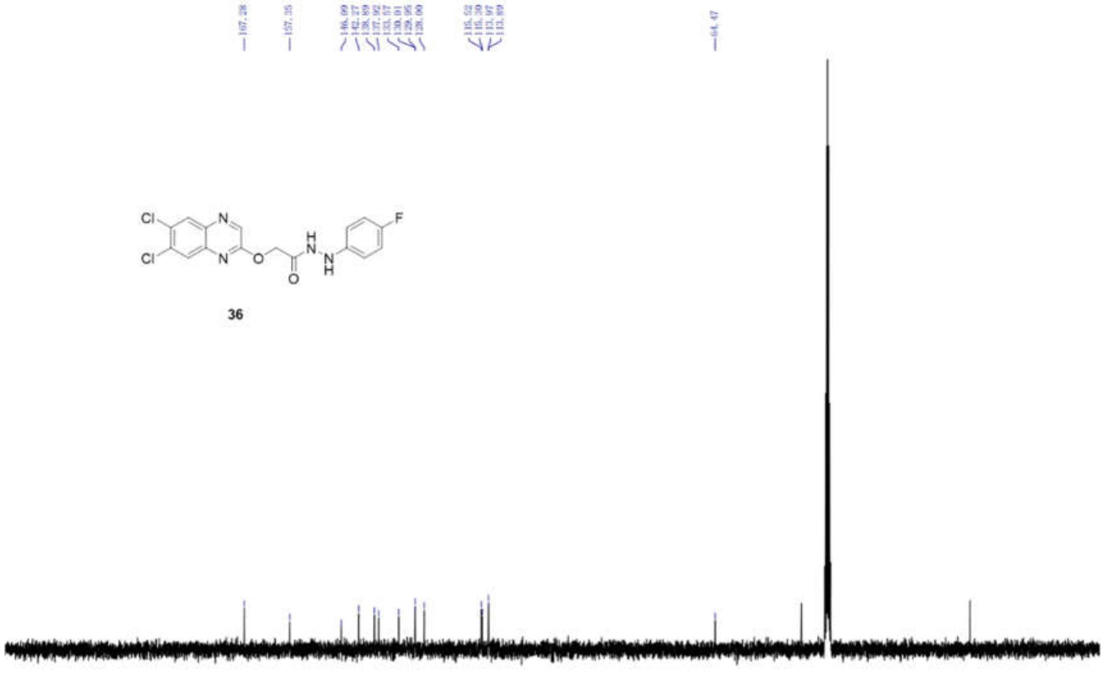

36
f1 (ppm)
